# Genome-wide association of polygenic risk extremes for Alzheimer's disease in the UK Biobank

**DOI:** 10.1038/s41598-022-12391-2

**Published:** 2022-05-19

**Authors:** Catarina Gouveia, Elizabeth Gibbons, Nadia Dehghani, James Eapen, Rita Guerreiro, Jose Bras

**Affiliations:** 1 Department of Neurodegenerative Science, Van Andel Research Institute, 333 Bostwick Ave. N.E., Grand Rapids, MI 49503-2518 USA; 2 Division of Psychiatry and Behavioral Medicine, Michigan State University College of Human Medicine, Grand Rapids, MI USA

**Keywords:** Computational biology and bioinformatics, Genetics, Neuroscience

## Abstract

In just over a decade, advances in genome-wide association studies (GWAS) have offered an approach to stratify individuals based on genetic risk for disease. Using recent Alzheimer's disease (AD) GWAS results as the base data, we determined each individual's polygenic risk score (PRS) in the UK Biobank dataset. Using individuals within the extreme risk distribution, we performed a GWAS that is agnostic of AD phenotype and is instead based on known genetic risk for disease. To interpret the functions of the new risk factors, we conducted phenotype analyses, including a phenome-wide association study. We identified 246 loci surpassing the significance threshold of which 229 were not reported in the base AD GWAS. These include loci that showed suggestive levels of association in the base GWAS and loci not previously suspected to be associated with AD. Among these, there are loci, such as *IL34* and *KANSL1*, that have since been shown to be associated with AD in recent studies. We also show highly significant genetic correlations with multiple health-related outcomes that provide insights into prodromal symptoms and comorbidities. This is the first study to utilize PRS as a phenotype-agnostic group classification in AD genetic studies. We identify potential new loci for AD and detail phenotypic analysis of these PRS extremes.

## Introduction

Alzheimer's disease (AD) is one of the most common, disabling neurodegenerative diseases faced by our society^1^. Heritability estimates from twin studies range from 60 to 80%^2^, suggesting a strong genetic component to the disease. However, a significant fraction of the phenotypic variance of the disease is unexplained by the currently known genome-wide significant loci^3^. Over the last decade, increasing sample sizes in AD genome-wide association studies (GWAS) have greatly improved the statistical power to detect novel genetic associations^4–8^. In addition, recent studies have characterized novel rare variability in the disease, furthering our understanding of genetic mechanisms underlying AD^9^.

Although increasing sample size is a tested approach to identify new loci in complex disease research, innovative approaches to further investigate these in large datasets may harbor further insights into the currently missing heritability.

Polygenic risk scores (PRS) have been used to understand the genetic liability of developing specific traits^10^. PRS are calculated from a set of independent variants associated with the disease or trait under study^11^, and a score is then assigned to each individual by considering the sum of weighted genetic effects previously associated with the phenotype. Studies applying PRS to clinically diagnosed AD patients have shown a predictive accuracy higher than 80%^12,13^, which suggests there is potential for PRS to be used as a future clinically valuable tool. PRS have also been utilized to prioritize individuals for screening of rare variants by identifying those with common diseases but low PRS^14^.

Here, we apply a PRS derived from a recent, large GWAS in AD^7^ to the UK Biobank (UKBB). We perform genetic association of common variants using individuals belonging to PRS extremes. We analyze these genetic associations alongside the extensive phenotypic and clinical information available in the UKBB.

## Results

### Polygenic risk scores

**Figure 1:**
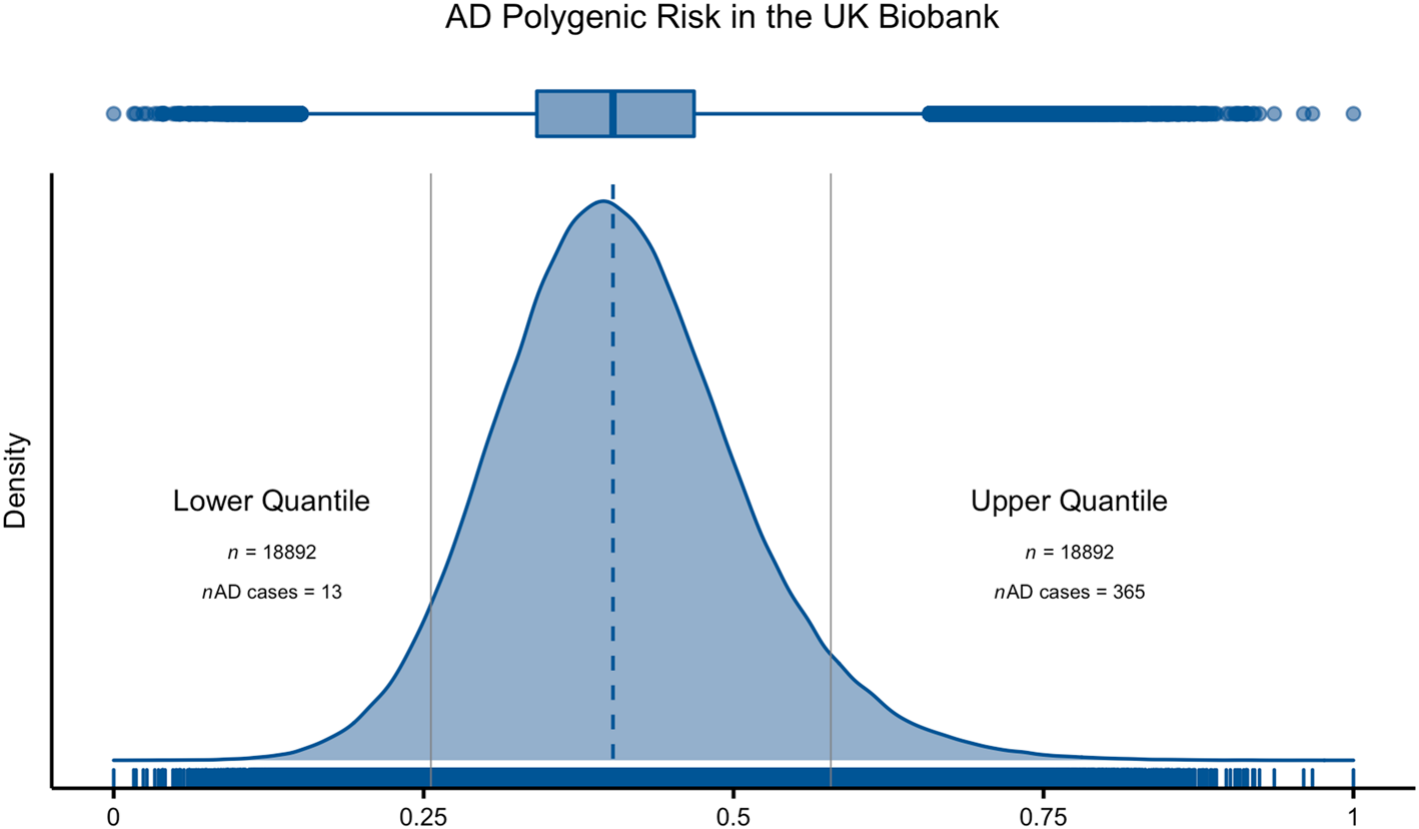
Density plot with the distribution of PRS when applied to samples from the UKBB. Solid grey vertical lines depict the 5% extremes of the PRS distribution. PRS were rescaled between zero and one.

We computed polygenic risk scores (PRS) for all unrelated genetically defined Caucasian individuals in the UKBB (*n* = 377,834), based on the summary statistics of a recent AD GWAS^7^. PRS were determined based on 176,316 variants remaining after clumping (Fig. 1).

### GWAS: genome-wide association study using AD PRS extremes

Using the individuals falling in each PRS extreme (Lower Quantile vs. Upper Quantile in Fig. 1), we performed a GWAS comparing these two groups. There was an inflation in the genomic inflation factor [λ = 2.442; see Supplementary Fig. 1 for a quantile–quantile (QQ) plot], which was expected given the approach of separating individuals based on their genetic risk.

We identified a total of 246 loci (473 lead SNPs) that met the genome-wide significance criteria (*p* < 5 × 10^–8^) (Supplementary Table 1). We refer to loci by the nearest gene where each lead SNP was annotated, as defined by FUMA.

**Figure 2:**
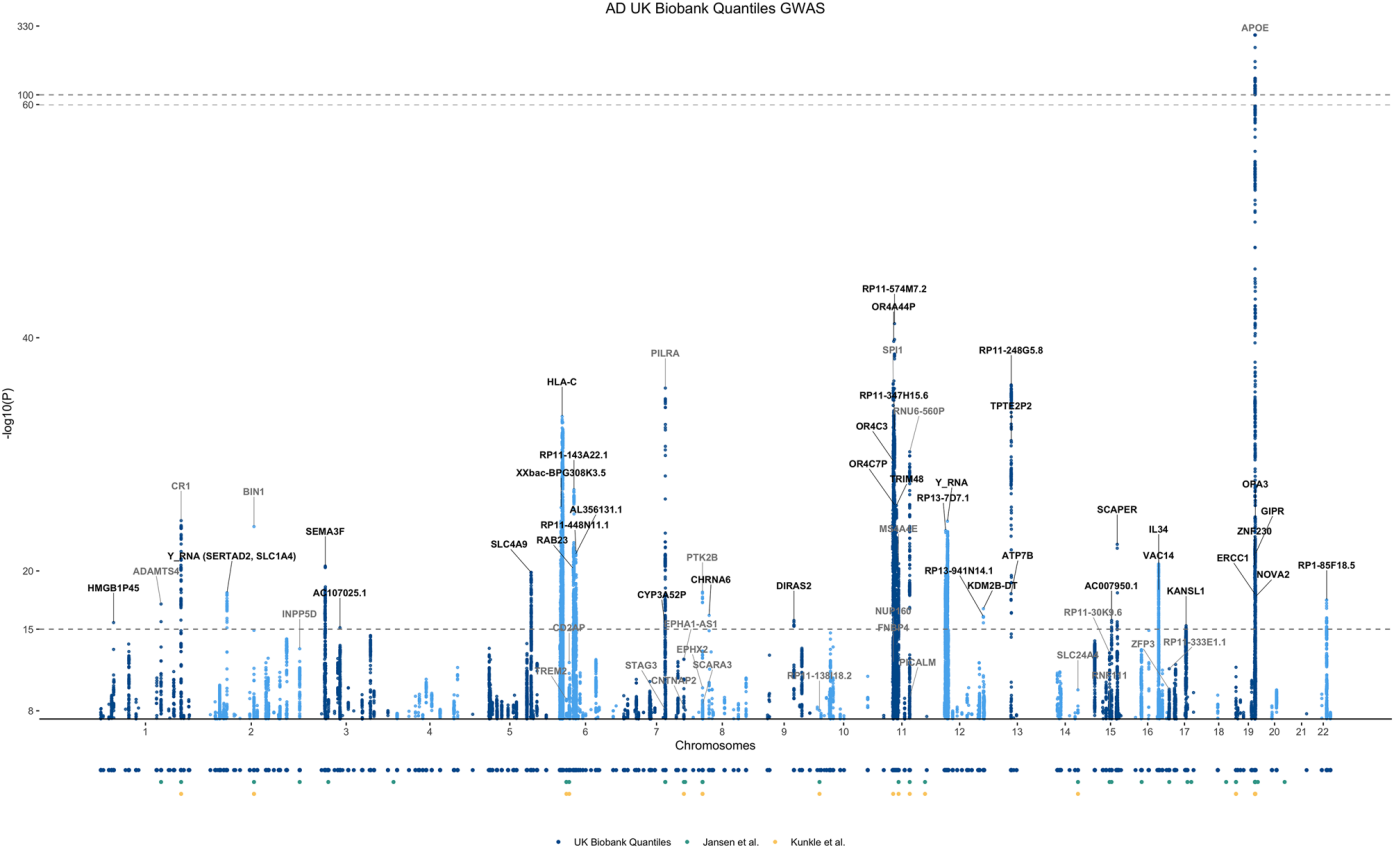
Manhattan plot of the GWAS performed in AD PRS extremes. We compared results with those from Refs.^7,8^. Genomic loci are labeled with the gene name that is nearest to that position. Black labels indicate novel signals; grey labels show previously replicated loci. The bottom panel shows the location of the significant variants depicted in the Manhattan plot that reached significance in the studies considered. Signals were annotated with FUMA^15^ and LocusZoom^16^.

We identified 23 loci below a *p* value = 1 × 10^–15^ that were not present in the base GWAS used for the PRS calculation. These are reported in Table 1 and highlighted in black in Fig. 2. Some of these new signals were mapped to genes not previously associated with AD, e.g.: *HMGB1P45, RAB23, DIRAS2, SCAPER* and *TRIM48*.

**Table 1:** Top SNPs with *p* values below 1E−15 in UKBB GWAS of AD PRS extremes and not associated with AD in the base GWAS.

CHR	Position	Genomic locus	SNP	Nearest gene	MAF	OR [95% CI]	*p* value
1	50861622	5	rs7553439	*HMGB1P45*	0.23	0.86 [0.84–0.9]	2.71E−16
2	65053188	31	rs62137344	*Y_RNA (SERTAD2, SLC1A4)*	0.12	0.82 [0.78–0.86]	7.43E−19
3	50204745	48	rs3774745	*SEMA3F*	0.47	0.87 [0.85–0.9]	3.85E−21
3	84464459	53	3:84464459_GA_G	*AC107025.1*	0.42	0.89 [0.86–0.91]	7.41E−16
5	139741370	89	rs717097	*SLC4A9*	0.47	0.87 [0.85–0.9]	1.33E−20
6	26907831	95	rs9379945	*GUSBP2*	0.15	1.2 [0.14–0.22]	5.19E−18
6	28750876	95	rs200690674	*NOL5BP*	0.1	1.26 [0.18–0.28]	8.67E−20
6	28790373	95	rs146924495	*LINC01623/XXbac-BPG308K3.5*	0.18	1.23 [0.17–0.25]	3.66E−26
6	29379304	95	rs3117190	*OR5V1*	0.84	0.84 [0.81–0.87]	3.58E−18
6	29604264	95	rs9461540	*SUMO2P1*	0.13	0.83 [0.79–0.86]	4.62E−18
6	31221299	95*	rs17197839	*HLA-C*	0.13	0.76 [0.73–0.79]	5.70E−34
6	57121684	103	rs6904307	*RAB23*	0.11	1.24 [1.19–1.3]	6.18E−21
6	57922673	103	rs6916215	*RBBP4P3*	0.28	1.15 [1.11–1.18]	3.28E−16
6	58677437	103	rs2693062	*RP11-143A22.1/AL445250.1*	0.33	0.84 [0.82–0.87]	1.08E−27
6	62101394	104	rs62425025	*AL356131.1*	0.42	1.16 [1.12–1.19]	3.86E−22
6	63162857	104	rs9360446	*RP11-448N11.1*	0.34	0.86 [0.83–0.89]	2.35E−22
6	63820771	104	6:63820771_AC_A	*RP11-184C23.1*	0.2	1.19 [1.14–1.23 ]	2.13E−20
7	99471072	128	rs2099446	*CYP3A52P*	0.46	0.89 [0.86–0.91]	6.95E−16
8	42667432	137	rs62515894	*CHRNA6*	0.06	1.31 [1.23–1.4]	6.51E−17
9	93391288	150	rs183428791	*DIRAS2*	0.06	1.28 [1.21–1.36]	1.79E−16
11	47197153	167	rs75290815	*ARFGAP2*	0.07	1.32 [1.24–1.4]	8.66E−21
11	48346996	167	rs12794960	*OR4C3*	0.16	0.79 [0.76–0.83]	3.93E−30
11	48472051	167	rs61915439	*OR4C9P*	0.07	1.29 [1.21–1.36]	7.62E−18
11	48709133	167	rs75184591	*OR4A44P*	0.36	1.23 [1.19–1.27]	2.03E−40
11	50189874	167	11:50189874_CAA_C	*RP11-347H15.6*	0.82	0.79 [0.76–0.82]	8.50E−33
11	50468801	167	rs1813937	*RP11-574M7.2*	0.78	1.28 [1.23–1.32]	6.15E−42
11	51253295	167	rs4312050	*AC110283.1*	0.16	0.82 [0.79–0.86]	7.15E−22
11	51476467	167	rs4515954	*OR4C7P*	0.17	1.23 [1.19–1.28]	2.35E−26
11	54892370	168	rs58904316	*TRIM48*	0.17	1.24 [1.19–1.29]	2.44E−26
11	55567256	168	rs72918199	*OR5D14*	0.06	0.73 [0.68–0.77]	2.45E−23
12	34454301	177	rs7314457	*RP13-7D7.1/AK6P1*	0.54	1.16 [1.13–1.2]	3.47E−24
12	38666013	178	rs10880819	*Y_RNA (ALG10B)*	0.46	0.86 [0.83–0.88]	5.48E−25
12	122028904	187	rs28507431	*RP13-941N14.1*	0.12	1.21 [1.16–1.26]	8.09E−17
13	52496060	190	rs7988558	*ATP7B*	0.04	1.27 [1.2–1.34]	8.99E−19
13	52862570	190	rs9535966	*TPTE2P2*	0.05	1.55 [1.45–1.65]	1.11E−36
15	63760569	205	rs4984289	*AC007950.1*	0.44	1.13 [1.1–1.16]	1.79E−16
15	76772062	208	rs2469249	*SCAPER*	0.27	0.85 [0.82–0.88]	5.26E−23
16	70676478	217	rs12598456	*IL34*	0.35	1.16 [1.12–1.19]	2.52E−21
16	70728477	217	rs3785425	*VAC14*	0.07	1.3 [1.22–1.37]	4.63E−19
17	44257788	229	rs2696697	*KANSL1*	0.23	0.87 [0.84–0.9]	5.08E−16
19	44522357	239	rs73035978	*ZNF230*	0.11	1.25 [1.19–1.31]	3.70E−21
19	45235700	239	rs74607435	*snoZ6*	0.05	0.74 [0.69–0.79]	2.23E−19
19	45938019	239	rs143008566	*ERCC1*	0.05	0.73 [0.68–0.78]	1.07E−18
19	46049982	239	rs10422253	*OPA3*	0.78	1.21 [1.16–1.25]	3.35E−25
19	46165082	239	rs112972879	*GIPR*	0.37	1.16 [1.13–1.2]	3.47E−22
19	46428653	239	rs9789319	*NOVA2*	0.5	1.14 [1.11–1.17]	1.63E−18
22	41587556	244	rs9607782	*EP300-AS1/RP1-85F18.5*	0.25	1.16 [1.12–1.2]	3.14E−18

Variants with *p* value ≤ 1 × 10^–15^ in the UKBB GWAS of PRS extremes not reported as associated in the base GWAS^7^. SNP positions are in GRCh37/hg19. Genomic locus is the index of the genomic risk loci defined by independent lead SNPs and maximum distance between their LD block (> 250 kb apart), defined according to FUMA.

**Figure 3:**
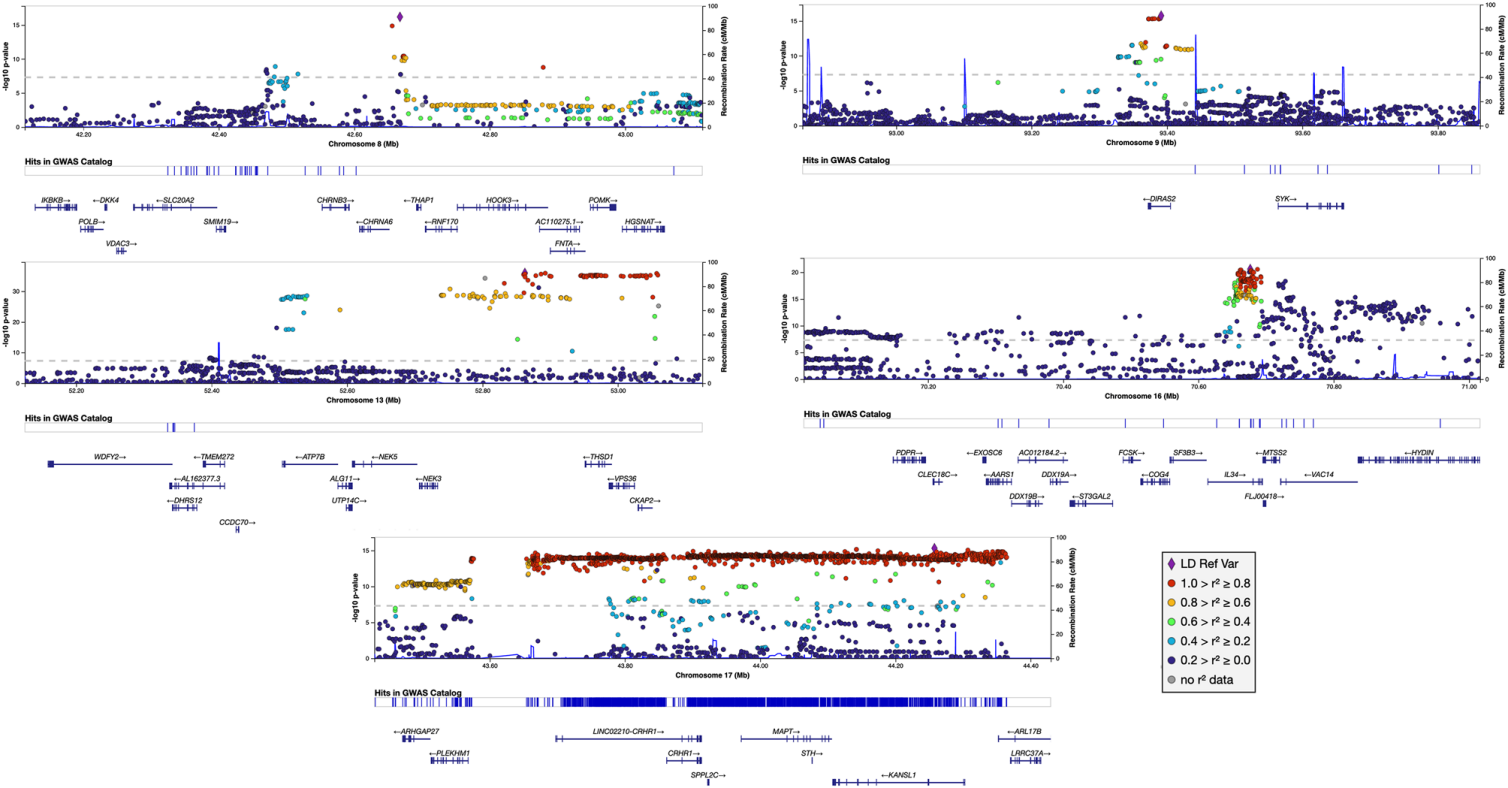
Regional association plots for significant loci in the GWAS performed in AD PRS extremes that had previously been implicated in AD in studies other than typical GWAS, but were not significant from the base AD GWAS.

We also show a comparison between these findings and the main findings reported in the base study^7^ in Table 2. Several significant results spanned genes that at the time of the base GWAS study, had only been implicated in AD in studies other than typical GWAS: *CHRNA6*^17^, *ATP7B*^18^, *IL34*^19^, *VAC14*^20^, *KANSL1*^21^, *NOVA2*^22^ (Fig. 3).

**Table 2:** Significant variants reported by Ref.^7^.

Locus						Base GWAS^7^		UKBB GWAS of AD PRS extremes			Stage 1^8^
CHR	Position	A2	A1	Nearest gene	SNP	OR	*p* value	OR	*p* value	Lowest *p* value variant at locus	Lowest *p* value variant at locus
1	161155392	G	A	*ADAMTS4*	rs4575098	1.02	2.05E−10	1.16	6.90E−18	rs4575098 (6.90E−18)	rs72702127 (3.03E−4)
1	207786828	G	A	*CR1*	rs2093760	1.02	1.10E−18	1.21	1.64E−23	rs4844610 (4.87E−25)	rs679515 (1.56E−16)
2	127891427	A	C	*BIN1*	rs4663105	1.03	3.38E−44	1.17	1.57E−24	rs4663105 (1.57E−24)	rs6733839 (4.02E−28)
2	233981912	C	G	*INPP5D*	rs10933431	0.98	8.92E−10	0.90	8.91E−10	rs36133610 (4.78E−14)	rs10933431 (2.55E−7)
3	57226150	C	T	*HESX1*	rs184384746	1.22	1.24E−08	–	–	rs79762933 (1.08E−9)	rs1565377 (4.67E−4)
4	11026028	G	A	*CLNK*	rs6448453	1.01	1.93E−09	1.09	4.75E−07	rs55706526 (3.87E−7)	rs4351014 (1.96E−5)
4	11723235	G	A	*HS3ST1*	rs7657553	1.01	0.051	1.02	0.19	rs55706526 (3.87E−7)	rs4351014 (1.96E−5)
6	32583357	A	T	*HLA-DRB1*	rs6931277	1.00	8.41E−11	0.82	3.73E−25	rs9274812 (1.87E−33)	rs201239900 (2.24E−9)
6	40942196	G	A	*TREM2*	rs187370608	1.26	1.45E−16	–	–	rs9394764 (1.01E−9)	rs75932628 (2.95E−12)
6	47432637	T	C	*CD2AP*	rs9381563	1.01	2.52E−10	1.11	6.13E−12	rs1385742 (7.24E−13)	rs1385742 (2.23E−8)
7	99971834	G	A	*ZCWPW1/PILRA*	rs1859788	0.98	2.22E−15	0.82	1.72E−35	rs2906657 (2.08E−36)	rs60738304 (1.15E−5)
7	143108158	C	T	*EPHA1*	rs7810606	0.99	3.59E−11	0.90	3.72E−13	rs7810606 (3.72E−13)	rs11767557 (1.56E−8)
7	145950029	C	T	*CNTNAP2*	rs114360492	1.20	2.10E−09	–	–	rs73742508 (4.24E−4)	rs62483962 (1.83E−4)
8	27464929	G	A	*CLU/PTK2B*	rs4236673	0.98	2.61E−19	0.93	8.46E−07	rs57735330 (6.52E−19)	rs867230 (3.49E−17)
10	11717397	T	C	*ECHDC3*	rs11257238	1.01	1.26E−08	1.08	2.14E−07	rs7912495 (9.20E−9)	rs12416487 (3.42E−8)
11	59958380	C	A	*MS4A6A*	rs2081545	0.98	1.55E−15	0.87	6.65E−21	rs367670643 (2.36E−21)	rs1582763 (1.19E−16)
11	85776544	A	G	*PICALM*	rs867611	0.98	2.19E−18	0.84	7.21E−29	rs10792832 (6.03E−31)	rs3851179 (5.81E−16)
11	121435587	T	C	*SORL1*	rs11218343	0.96	1.09E−11	–	–	rs1133174 (1.21E−5)	rs11218343 (2.63E−8)
14	92938855	G	A	*SLC24A4*	rs12590654	0.99	1.65E−10	0.91	1.29E−08	rs35627364 (1.59E−10)	rs12590654 (8.73E−9)
15	59022615	T	C	*ADAM10*	rs442495	0.99	1.31E−09	0.91	8.52E−10	rs602602 (1.04E−13)	rs383902 (3.81E−6)
15	63569902	C	T	*APH1B*	rs117618017	1.01	3.35E−08	1.10	4.60E−06	rs4984289 (1.79E−16)	rs12913805 (1.59E−5)
16	31133100	G	A	*KAT8*	rs59735493	0.99	3.98E−08	0.91	4.90E−09	rs7499339 (5.76E−14)	rs201827363 (2.06E−3)
17	5138980	G	A	*SCIMP*	rs113260531	1.02	9.16E−10	1.15	8.13E−10	rs78538460 (2.48E−12)	rs61182333 (2.18E−5)
17	47450775	G	A	*ABI3*	rs28394864	1.01	1.87E−08	1.07	1.40E−05	rs850522 (6.57E−10)	rs2960170 (1.52E−3)
17	56409089	G	C	*BZRAP1-AS1*	rs2632516	0.99	9.66E−07	0.93	3.68E−06	rs1985749 (1.15E−7)	rs2632516 (3.67E−7)
18	29088958	C	T	*SUZ12P1*	rs8093731	0.98	0.03	–	–	rs7240561 (9.92E−5)	rs189640326 (3.18E−4)
18	56189459	T	C	*ALPK2*	rs76726049	1.06	3.30E−08	–	–	rs35597325 (5.59E−5)	rs187113635 (1.08E−4)
19	1039323	C	G	*ABCA7*	rs111278892	1.02	7.93E−11	1.15	1.18E−11	rs3752231 (1.84E−12)	rs12151021 (2.56E−10)
19	45351516	C	G	*APOE*	rs41289512	1.23	5.79E−276	3.44	5.64E−212	rs814573 (1.62E−673)	rs429358 (1.17E−881)
19	46241841	C	T	*AC074212.3*	rs76320948	1.04	4.64E−08	–	–	rs123187 (8.72E−27)	rs181476525 (8.29E−13)
19	51727962	C	A	*CD33*	rs3865444	0.99	6.34E−09	0.94	4.74E−05	rs1354106 (1.13E−5)	rs3865444 (3.93E−7)
20	54998544	A	G	*CASS4*	rs6014724	0.98	6.56E−10	0.92	1.88E−03	rs1059768 (2.56E−7)	rs6014724 (3.65E−7)

Lowest *p* value columns are defined as 500 kb flanking the original reported variant. SNP positions are in GRCh37/hg19.

Of the 44 loci currently associated with AD and present in the GWAS Catalog (version e96_r2019-09-24) we directly replicate 28 (Supplementary Tables 2 and 3). The 16 loci not replicated in our study included 6 that were not assayed due to low MAF or genotyping call rate of the reported SNPs (*HESX1*, *MEF2C*, *SHARPIN*, *SORL1*, *SUZ12P1*/*DSG2*, *ALPK2*). The 10 loci that remained non-replicated included 3 that were borderline significant in our GWAS (*CLNK/HS3ST1*, *BZRAP1-AS1*, and *CASS4*) and 7 that were not close to genome-wide significance (*LCORL*, *ANKRD31*, *NME8*, *NDUFAF6*:*TP53INP1*, *PLCG2*, *CD33*, and *APP*). *LCORL*, *ANKRD31*, *NDUFAF6:TP53INP1* have only been seen in one GWAS each and do not have support from the most recent GWAS either^23^, most likely representing false positives. *PLCG2* is a well established locus that is not being replicated here. *APP* has been initially shown to be associated by GWAS by Ref.^24^ and reaches a significance level of *p* = 1.0 × 10^–12^ in Ref.^5^. *CD33* has been found to be significant and non-significant by several GWAS studies. The two most recent GWAS reflect this pattern with the locus showing a significance level of 2.21 × 10^−10^ in Ref.^23^ and not showing up in Ref.^5^ either as an established or new locus.

When comparing results with the base GWAS^7^, the most significant SNPs for *ADAMTS4, CR1, HLA-DRB1, CD2AP, ZCWPW1/PILRA, EPHA1, MS4A6A, PICALM, ADAM10, KAT8, SCIMP,* and *ABCA7* were all more significant in this study. Similarly, to the comparison with the loci present in the GWAS catalog, we could not replicate some of the initial findings due to the SNP frequency being lower than our inclusion threshold. Individual inspection of these variants revealed several of them had a higher frequency in the high PRS group than the low PRS, showing the same direction of effect (Table 2). For example, rs187370608, in TREM2, had a frequency twice as high in the high PRS group compared to the low PRS group (MAF: 4.9 × 10^–3^ vs. 2.2 × 10^–3^). Exceptions were rs11218343 in *SORL1* and the *SUZ12P1* locus that did not show significant differences between groups*.* In addition to SNPs that were below our MAF threshold, there were others that we did not replicate, and these were either borderline significant in our data, or were not further replicated by more recent AD GWAS. Conversely, some loci that were sub-significant in the base GWAS reached significance in this analysis*.* Some loci, such as *IL34*, that were not significant in the Jansen GWAS, have surpassed the significance threshold in our study and have also been independently shown to be associated with AD^5^.

### Phenotype-based gene set enrichment

**Figure 4:**
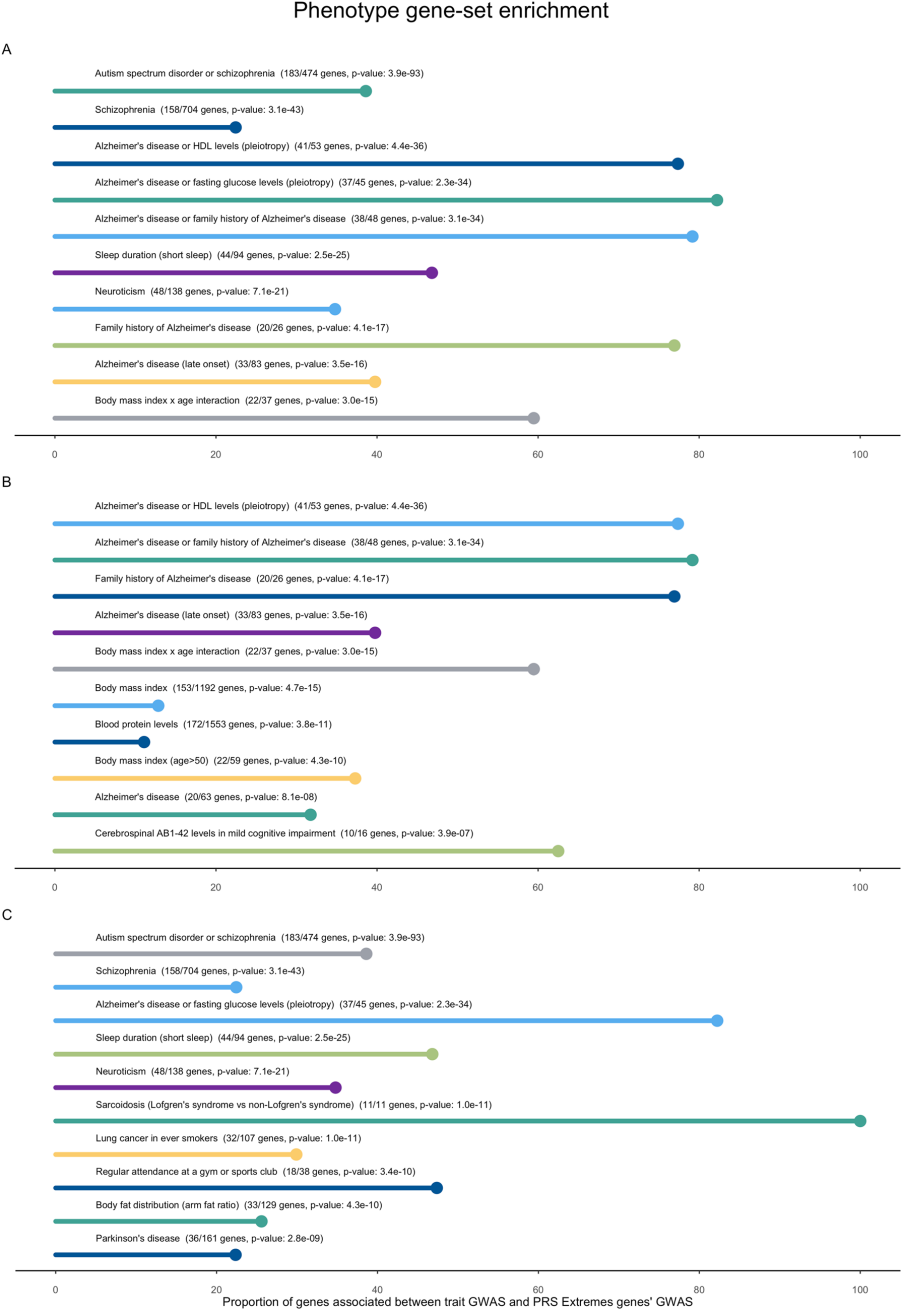
Phenotype-based gene set enrichment from GWAS Catalog. The top 10 most significant traits are shown in (**A**). The top 10 most significant traits where the overlapping genes include genes located in the *APOE* locus (chr19q13) are shown in (**B**) and the top 10 most significant traits where the overlapping genes do not include genes located in this locus are shown in (**C**).

To determine if there were sets of genes associated with other phenotypes enriched in AD PRS extremes, we performed a gene set enrichment analysis using FUMA (Supplementary Table 4). In Fig. 4 we report the top 10 most significant GWAS Catalog traits where genes overlap between the GWAS results for each trait and the GWAS results from the AD PRS extremes. To consider the strong effect of the *APOE* locus we separated results according to the presence (Fig. 4B) or absence (Fig. 4C) of genes located in this locus in the resulting overlapping gene sets.

#### Genetic correlation

**Figure 5:**
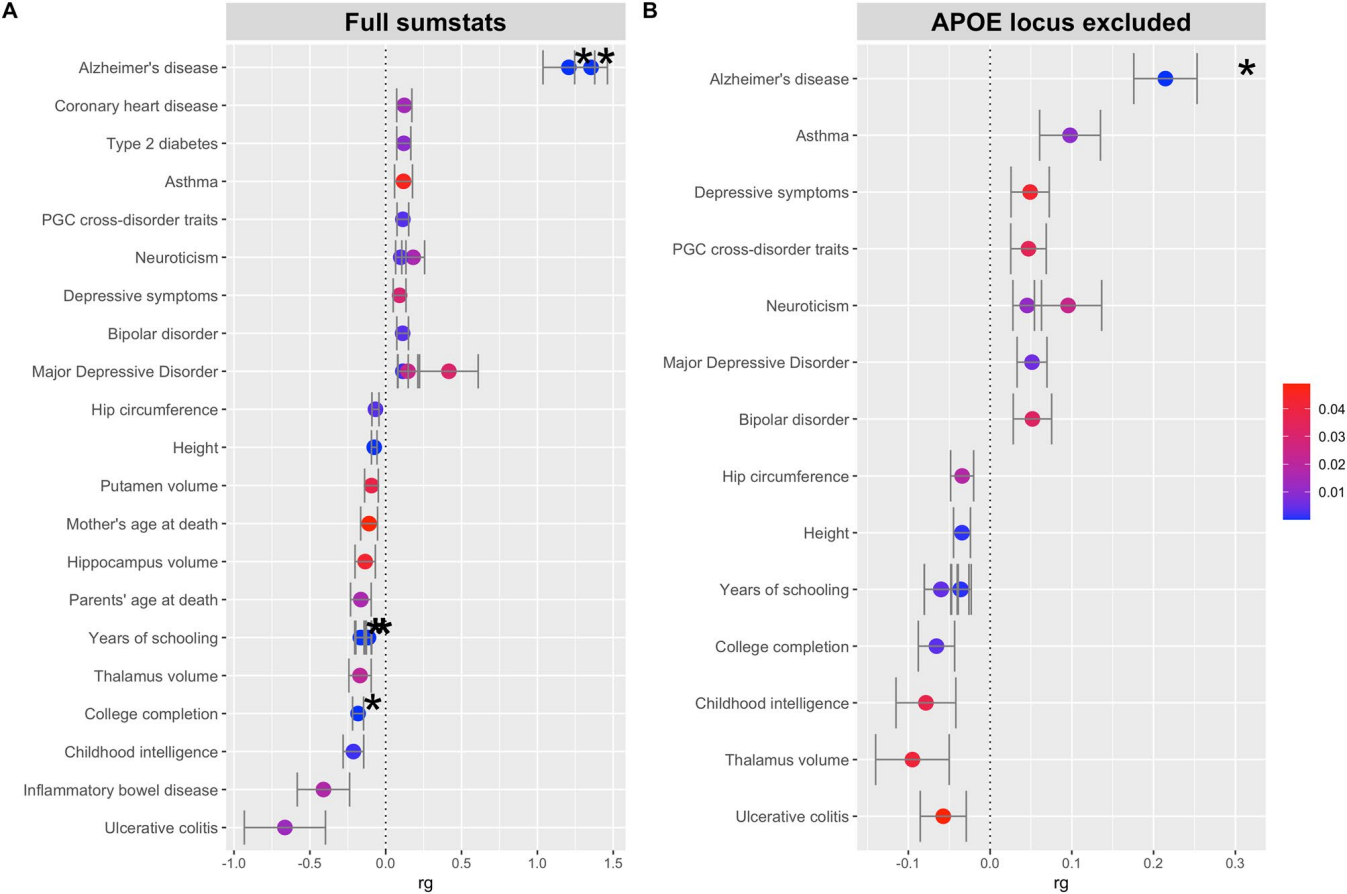
Genetic correlation of most significant MRC IEU traits. Most significant (p < 0.05) correlations from ldsc analysis of OpenGWAS ieu-a traits. Results are shown for the full sumstats (no loci excluded) (**A**) and in the absence of the APOE locus (**B**). Color of points denotes p value. Asterisk indicates p value below 1e−5.

We performed a genetic correlation to determine the relationship to other traits associated with these loci. In Fig. 5 we report the most significant correlated traits when analyzing all datasets available within the “ieu-a” batch available in the OpenGWAS project from the MRC Integrative Epidemiology Unit (IEU) (Fig. 5A). Again, to account for the strong effect of APOE we also conducted this analysis excluding the APOE locus (Fig. 5B). Results for all correlations performed are available in Supplementary Table 6.

#### PheWAS

To determine if any of the phenotypes reported in the UKBB dataset were associated with extreme genetic risk for AD and potentially find traits that could be prodromal in AD, we performed a PheWAS using 1424 traits. We also performed the same analysis to understand if the associations were being driven by *APOE*, by excluding the *APOE* locus from the underlying GWAS.

**Figure 6:**
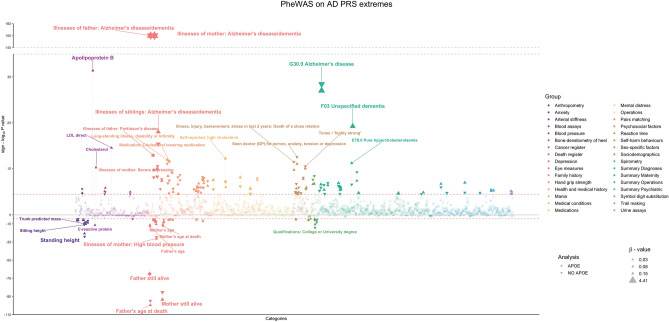
Phenome Scan results. The analysis was made with individuals in each extreme of the PRS distribution (including and excluding the *APOE* locus) using PHESANT. Each color represents a group of traits/diseases, according to the UKBB hierarchical tree. Downward triangles represent results from individuals in the extremes of the PRS calculated when excluding *APOE* (no APOE). The size of triangles represents the beta value for the association. Vertical dashed lines connect results for the same trait in the APOE and no APOE analyses. Y-axis is the logarithmic scale for the *p* value, multiplied by the beta value, to depict whether the trait is negatively or positively associated. Red dashed lines represent the adjusted Bonferroni *p* value threshold. *Note: "Illnesses of father/mother: Alzheimer's Disease/Dementia" had a software output *p* value of 0, to represent these we attributed a *p* value of 1 × 10^–150^.

We focused on associations that surpassed the adjusted *p* value threshold and had a *β* ≥ |0.5| (Fig. 6, Table 3).

**Table 3:** PheWAS results for AD PRS extremes.

UKBB trait	APOE		Excl. APOE	
	β (95% CI)	p value	β (95% CI)	p value
Illnesses of mother: Alzheimer's disease/dementia	4.41 (4.29, 4.54)	1 × 10^–150^*	4.37 (4.25, 4.50)	1 × 10^–150^*
Illnesses of father: Alzheimer's disease/dementia	3.96 (3.79, 4.13)	1 × 10^–150^*	3.95 (3.79, 4.12)	1 × 10^–150^*
Mother still alive	− 0.51 (− 0.55, − 0.46)	3.42 × 10^–94^	− 0.51 (− 0.55, − 0.46)	3.42 × 10^–94^
Father still alive	− 0.51 (− 0.57, − 0.45)	1.44 × 10^–65^	− 0.52 (− 0.57, − 0.46)	4.24 × 10^–66^
Alzheimer's disease, unspecified	3.22 (2.69, 3.86)	9.7 × 10^–28^	2.89 (2.42, 3.43)	3.74 × 10^–29^
Unspecified dementia**	2.00 (1.59, 2.45)	5.72 × 10^–20^	–	–
Illnesses of siblings: Alzheimer's disease/dementia	1.21 (0.95, 1.48)	9.85 × 10^–19^	1.10 (0.83, 1.36)	4.36 × 10^–16^
Parkinson’s disease in mother	0.51 (0.36, 0.66)	9.61 × 10^–11^	0.45 (0.30, 0.60)	5.69 × 10^–9^
Tendency to fall, not elsewhere classified	0.54 (0.32, 0.76)	1.12 × 10^–6^	0.54 (0.32, 0.76)	1.45 × 10^–6^
Ginkgo forte tablet	0.58 (0.34, 0.83)	2.24 × 10^–6^	0.50 (0.27, 0.74)	3.20 × 10^–5^
Unspecified disorientation**	0.55 (0.32, 0.78)	3.07 × 10^–6^	–	–
Faecal incontinence	0.58 (0.31, 0.85)	2.51 × 10^–5^	0.57 (0.30, 0.85)	4.67 × 10^–5^

Shown are results with adjusted *p* value ≤ 4.3 × 10^–5^ and *β* ≥ |0.5|. *APOE* indicates the PheWAS using individuals from the PRS extremes when *APOE* was included; Excl. *APOE* indicates PheWAS using individuals in the PRS extremes when the *APOE* locus was excluded from the PRS analysis (see “Methods”).

*p value was too significant, and software output was zero—to plot these values (Fig. 6), we attributed a *p* value of 1 × 10^–150^.

**Traits were excluded from PheWAS analysis using individuals from the PRS calculated without *APOE* due to lack of representation.

Family history of AD or dementia (represented by parents and by siblings) was significantly associated with the AD PRS extremes. Interestingly, a proxy to longevity in the parents (mother and father still alive) was also associated with AD PRS extremes but in the opposite direction. Other traits also significantly associated with AD PRS extremes included tendency to fall, fecal incontinence, Parkinson's disease in mother, and usage of Gingko forte as medication.

## Discussion

Given the difficulty in assembling ever larger cohorts of well characterized AD cases and the fact that other genetic loci contributing to disease risk are still to be identified, it is important to find new analytical methods to fully characterize the genetic architecture of Alzheimer’s disease. Here we used an alternative approach to the typical GWAS by performing an association study on genetic risk extremes for AD. The rationale for this approach is based on the hypothesis that by taking a population of individuals and enriching those that carry variants that are of risk for a phenotype, one is also enriching for other variants associated with that same phenotype. Thus, we are using the extreme polygenic risk of AD as a surrogate for disease status and the variants identified here are not necessarily associated with AD itself but with the polygenic risk of AD. In fact, of the 18,892 individuals included in the high PRS extreme, only 365 were reported to have a diagnosis of AD. It is important to note that we are not using the PRS to predict AD status; this would suffer from overfitting since the UKBB samples were used in the study in our base summary statistics. In short, we are separating individuals in the UKBB population solely by their PRS for AD, selecting the extremes of this distribution, and then testing their genetic and phenotypic differences.

In this approach, we used 38,000 individuals selected from the PRS extremes obtained for almost 400,000 individuals in the UKBB. By comparing these genetic risk extremes, we were able to identify 23 loci with *p* values of association below 1 × 10^–15^ that were not shown as associated with AD in the base GWAS study. Among these, the identification of genome-wide significant signals at the *IL34, ACE* and *KANSL1* loci—loci that were not significant in the base GWAS but were subsequently identified to be associated with AD in independent studies—shows the validity of this approach. These results also offer the possibility of auditing the many loci now associated with AD risk. Comparing the results from the recent GWAS studying over 1 million individuals^23^ and the previous results by the same group (base GWAS), using largely data on the same samples it is interesting to note that 8 loci that were significant in the base GWAS have not been found to be associated with AD in the larger, more recent study: *ADAMTS4*, *HESX1*, *HS3ST1*, *CNTNAP2*, *KAT8*, *SUZ12P1 (DSG2)*, *ALPK2* and *AC074212.3*. Four of these loci are significant in this GWAS of AD PRS extremes: *ADAMTS4*, *CNTNAP2*, *KAT8* and *AC074212*.3. *ADAMTS4* is an interesting locus as it has not been associated with AD by any other GWAS but shows a very significant association in this GWAS of AD PRS extremes (*p* = 6.9 × 10^–18^) and functionally is very relevant for the beta-amyloid pathway^25,26^. On the other hand, three of the four loci that were not replicated in this study (*HESX1, SUZ12P1* and *ALPK2*) all have top SNPs that were not included in this study due to low MAF. Still, there is no hint of association in any of these loci and they have not been associated with disease in more recent GWAS, indicating these may be false positives. Also interesting to note is the *CD33* locus that has repeatedly been found to be significantly associated (including in the base GWAS), or to not associate with AD risk in the main GWAS for the disease. These inconsistent results may reflect an association that is stronger, or only present in some populations, but can also represent a false positive. The most significant variant in this locus in this GWAS of AD PRS extremes reached a *p* value of 10^–5^, supporting the latter. Still, the several studies showing different effects of *CD33* in AD, such as an elevated expression in AD brain associated with amyloid pathology, disease progression, and microglial activation may reflect the important role of CD33 biological pathway in AD, most likely dependent on TREM2^27^.

Given the design of this study, it is not possible to perform a formal replication stage to confirm the novel loci identified that potentially associate with AD risk. Still, some of the loci have now been identified in other GWAS (e.g. *WDR12* and *DOC2A*)^5^ and many of the genes nominated in the loci have features suggesting a potential role in AD. This recent GWAS also identified *GRN* and *TMEM106B* as novel loci for AD and suggested a continuum between AD and FTD. Interestingly, our results identified several loci that have been previously associated with the risk of Parkinson’s disease (e.g.: *LRRK2*, *ITKB, CCDC62*), but no loci overlapping with frontotemporal dementia in addition to the *MAPT* locus. This indicates that instead of a continuum between AD and FTD at the GWAS risk level, the identification of FTD loci most likely reflected the misclassification in the diagnoses of clinical AD and proxy-AD. Misdiagnoses have always been part of GWAS, and these should be more apparent as the sample sizes increase with the inclusion of not-so-well characterized samples.

Both genetic correlation and gene set enrichment analyses identified interesting overlaps. Particularly, gene set overlap and genetic correlation can be observed with psychiatric traits such as schizophrenia, neuroticism, and depression. A previous association study of the shared genetics of autism spectrum disorder, attention deficit-hyperactivity disorder, bipolar disorder, major depressive disorder, and schizophrenia by the Psychiatric Genomics Consortium was one of the most significant correlations in this analysis. Etiologically and clinically, these psychiatric traits and AD are different diseases. Still, in many cases, they have similarities in the patterns of regional brain and biochemical dysfunctions, as well as in symptomatology^28^. Psychotic events are experienced by up to 50% of AD patients over the course of their illness^29^ and, when compared with the general population, individuals with schizophrenia have a significantly higher risk (2–4 times) of developing AD and other dementias^30^. It is interesting to note that the base GWAS reported a nominally significant genetic correlation between schizophrenia and AD^7^. More recently, by applying a schizophrenia PRS to AD with psychosis, it was shown that psychosis in AD shares some genetic liability with schizophrenia^31^.

It is also interesting that this approach using AD PRS extremes identified enrichment and correlation of genes overlapping with phenotypes such as sleep duration and neuroticism. A robust association of sleep duration in middle and old age with the incidence of dementia has recently been established, using the 25-year follow-up Whitehall II study^32^. It should also be noted that sleep duration is anticorrelated with risk of AD in our study. Similarly, neuroticism has been associated with the risk of AD but also with disease pathology and progression both in sporadic and autosomal dominant disease^33–35^.

When examining the presence of *APOE* in these gene sets, the most enriched phenotypes with prior association to *APOE* included body mass index, AD CSF biomarkers and HDL/LDL levels, and several of these enrichments are further corroborated with evidence for correlation of anthropometric traits in the genetic correlation analyses. This is indicative of the strong effect that *APOE* has on these phenotypes, but also that the approach to separate individuals based on their AD PRS captures an enrichment of genes directly associated with AD, but also AD-related phenotypes, such as CSF Abeta and tau levels. Excluding the *APOE* locus from the overlapping genes also showed an enrichment of AD-related phenotypes, but also of other diseases, such as sarcoidosis and Parkinson’s disease.

To explore the phenotypes associated with each quantile and potentially find new phenotypes and traits that could be seen as either comorbidity or predicting factors for AD, we also performed a PheWAS, using more than 1000 traits available in the UKBB dataset.

As expected, AD diagnosis and AD in the family were the most significant phenotypes associated with the AD PRS extremes comparison. Tendency to fall has been previously shown to be significantly higher in a small cohort of 140 AD patients versus 137 controls^36^, a result that we replicate in this study. The use of Ginkgo Forte was also significantly associated in these results, which could be a result driven by individuals with a family history of dementia searching for pharmaceutical options to improve or maintain memory. Parental longevity is inversely associated with AD PRS since individuals in the high PRS group seemed to have higher mortality in their parents. Previous studies have also reported that individuals with parents who live longer tend to have a more preserved brain structure and lower evidence of AD^37,38^.

There are a few features of the approach taken in this study that need to be kept in mind when interpreting these results: as previously mentioned, an extreme PRS for AD does not equate to a clinical diagnosis of AD. The associations described here are not with AD itself but rather with the genetic risk for AD. Related to this, high risk individuals may never develop AD, but they are still genetically predisposed to it. It is not possible to easily replicate the results obtained here, given the absence of a similar, independent dataset. Like most GWAS, this study also focuses on individuals of European ancestry—a feature of our method that utilizes the largest available "genetically homogenous" publicly available dataset, but an important aspect that is necessary to address in future studies^39^.

Using publicly available data from a previous GWAS on Alzheimer's Disease^7^ we computed polygenic risk scores for all genetically unrelated Caucasians in the UK Biobank cohort. To our knowledge, this is the first study using an AD PRS to separate individuals purely based on genetic risk, agnostic to disease status. We identified the two extremes of AD risk from the polygenic risk distribution and analyzed genetic and phenotypic differences between these groups.

The power of this unique approach allowed us to identify novel associations, not only at loci that were sub-significant in the base study but also at loci that were not suggestively significant. Some of the loci identified here have been recently and independently associated with AD by typical GWAS, validating this approach. Our findings indicate the urgent need of a systematic and comprehensive audit of all loci currently associated with AD risk. The inclusion of loosely characterized samples and the use of the same samples and/or data by different GWAS contributes to the difficulty in assessing true loci for the disease.

In summary, this is the first time PRS are used as the only defining characteristic to differentiate groups of individuals to identify novel loci associated with the underlying phenotype. Furthermore, we used phenotype analyses to identify comorbidities, traits, and diseases that can point towards new prodromal characteristics of high genetic risk for AD.

## Methods

### Dataset

We used the UKBB cohort, containing 487,409 whole-genome genotyped individuals (version 3)^40^, with about 200,000 of which also whole-exome sequenced (released in October 2020)^41^. This work was conducted as part of UK Biobank application number 11036 and follows all applicable guidelines and regulations. Individuals are from the United Kingdom and aged between 40 and 69 at recruitment^40^. We included individuals identified in the UKBB documentation as genetically defined "Caucasian" and removed individuals with greater than 3rd-degree relatedness to any other sample in the dataset, by applying a KING cutoff of 0.0884 as implemented in the ukbtools package (v0.11.3).

### Polygenic risk score

To derive polygenic risk scores, we applied PRSice-2^42^ to the summary statistics of one of the recent GWAS for AD^7^. Variants with *p* values below 0.05 in the AD GWAS were selected from the UKBB dataset and filtered to keep only variants with a Hardy–Weinberg equilibrium exact test *p* value above 1 × 10^–15^, missing call rates less than 1% and a minor allele frequency of at least 0.1% in the UKBB dataset. We used the following covariates throughout the analysis: sex, year of birth, Townsend deprivation index at recruitment, genotype measurement batch, and the first ten principal components provided by the UKBB. We defined quantiles from the PRS distribution with individuals in the 5% lowest PRS (18,892 individuals; 53.8% females, 46.2% males) and the 5% highest PRS (18,892 individuals; 54.6% females, 45.4% males). Individuals in the upper quantile will be referred to as "high PRS" individuals, while individuals in the lower quantile will be referred to as "low PRS" individuals. Additionally, to determine how much of the PRS was dependent on the *APOE* locus, we calculated PRSs using the complete set of markers and excluding SNPs within 1 Mb of the most significant variant in this locus, while using APOE genotype as a covariate.

### Genome-wide association study

We determine which individuals fall in the highest and lowest 5% of the PRS distribution and perform a GWAS using these PRS extremes as the classification of groups in an agnostic approach to the clinically defined phenotype. We adjusted this analysis with the same covariates used in the PRS analysis described above. We filtered out all variants with a minimum allele frequency below 5%, Hardy–Weinberg equilibrium exact test *p* value below 1 × 10^–6^, and missing call rates above 1%. Association analyses were performed using the logistic regression function in PLINK1.9^43^. We then used FUMA package v1.3.6a^15^ to annotate, analyze and interpret the results using the SNP2GENE function. All SNPs prioritized as the lead had a *p* value of less than or equal to 5 × 10^–8^.

Additionally, candidate SNPs were included in the annotation if they had a maximum *p* value of 0.05. Significant SNPs were considered as independent if they had a clumping R^2^ threshold of at least 0.6 while lead SNPs were prioritized from independent SNPs and only considered as such if they had an R^2^ threshold for the second clumping step of at least 0.1 (or if it was the same as the first clumping). We used Phase 3 of 1000 Genomes (European samples only) as a reference panel to assess linkage disequilibrium.

Genomic inflation was calculated for lambda (λ) in the QCEWAS package in R.

### Phenotype-based gene set enrichment

Using results from the GWAS applied to individuals in the extreme PRS, we performed gene set enrichment analyses through GENE2FUNC in the FUMA package v1.3.6a^15^. Positional gene mapping aligned significant SNPs (*p* value < 5 × 10^−8^) by their location within or immediately up/downstream [± 10 kilobases (kb)] of known gene boundaries. We report gene sets that had an overlap of at least two genes between the input list of genes (from SNP2GENE) and the gene sets that were significantly enriched at a maximum adjusted *p* value threshold of 0.05. Multiple test correction for gene-set enrichment was performed using the Benjamini–Hochberg (FDR) method^44^.

### Genetic correlation

A genetic correlation analysis was performed using LD score regression^45^. We analyzed traits available through the OpenGWAS platform^46^, specifically using the ieu-a batch, which has been well described elsewhere^47^. These summary statistics were filtered to only include datasets with more than 2000 male and female samples, and only those reported in European ancestry groups, yielding 149 datasets. These correlations were also performed in the absence of the APOE locus. All SNPs within the region of 19:45236729–45618959 (hg19) were excluded in this analysis.

### Phenome-wide association analysis (PheWAS)

We used PHESANT—PHEnome Scan ANalysis Tool^48^ to perform an automated phenome scan in the UKBB, using the PRS extremes GWAS. This analysis was performed including and excluding the *APOE* locus in the GWAS. Phenotypes with more than 20% missing answers were filtered out. We adjusted for sex, age at recruitment, Townsend deprivation index at recruitment, genotype measurement batch, and the first ten principal components. In addition, we considered phenotype categories with a minimum size of 200 answers and converted fields with multiple instances to categorical (multiple) fields as implemented in PHESANT. In total, 1424 traits were analyzed. *p* values were adjusted for multiple testing correction using Bonferroni.

## Supplementary Information

Supplementary Information 1.

Supplementary Information 2.

## References

[1] Chouraki V, Seshadri S (2014). Genetics of Alzheimer’s disease. Adv. Genet..

[2] Gatz M (2006). Role of genes and environments for explaining Alzheimer disease. Arch. Gen. Psychiatry.

[3] Nazarian A, Kulminski AM (2019). Evaluation of the genetic variance of Alzheimer’s disease explained by the disease-associated chromosomal regions. J. Alzheimers. Dis..

[4] Schwartzentruber J (2021). Author Correction: Genome-wide meta-analysis, fine-mapping and integrative prioritization implicate new Alzheimer’s disease risk genes. Nat. Genet..

[5] Bellenguez, C. *et al.* New insights on the genetic etiology of Alzheimer’s and related dementia. *medRxiv* (2020).

[6] Wightman DP (2020). Largest GWAS (N=1,126,563) of Alzheimer’s disease implicates microglia and immune cells. bioRxiv.

[7] Jansen IE (2019). Genome-wide meta-analysis identifies new loci and functional pathways influencing Alzheimer’s disease risk. Nat. Genet..

[8] Kunkle BW (2019). Genetic meta-analysis of diagnosed Alzheimer’s disease identifies new risk loci and implicates Aβ, tau, immunity and lipid processing. Nat. Genet..

[9] Perrone F, Cacace R, van der Zee J, Van Broeckhoven C (2021). Emerging genetic complexity and rare genetic variants in neurodegenerative brain diseases. Genome Med..

[10] Torkamani A, Wineinger NE, Topol EJ (2018). The personal and clinical utility of polygenic risk scores. Nat. Rev. Genet..

[11] Lewis CM, Vassos E (2020). Polygenic risk scores: From research tools to clinical instruments. Genome Med..

[12] Escott-Price V, Myers A, Huentelman M, Shoai M, Hardy J (2019). Polygenic risk score analysis of Alzheimer’s disease in cases without APOE4 or APOE2 Alleles. J. Prev. Alzheimers Dis..

[13] Chaudhury S (2019). Alzheimer’s disease polygenic risk score as a predictor of conversion from mild-cognitive impairment. Transl. Psychiatry.

[14] Lu T (2020). Individuals with common diseases but with a low polygenic risk score could be prioritized for rare variant screening. Genet. Med..

[15] Watanabe K, Taskesen E, van Bochoven A, Posthuma D (2017). Functional mapping and annotation of genetic associations with FUMA. Nat. Commun..

[16] Pruim RJ (2010). LocusZoom: Regional visualization of genome-wide association scan results. Bioinformatics.

[17] Altimiras F (2017). Brain transcriptome sequencing of a natural model of Alzheimer’s disease. Front. Aging Neurosci..

[18] Squitti R, Siotto M, Arciello M, Rossi L (2016). Non-ceruloplasmin bound copper and ATP7B gene variants in Alzheimer’s disease. Metallomics.

[19] Walker DG, Tang TM, Lue L-F (2017). Studies on colony stimulating factor receptor-1 and ligands colony stimulating factor-1 and interleukin-34 in Alzheimer’s disease brains and human microglia. Front. Aging Neurosci..

[20] Drange OK (2019). Genetic overlap between Alzheimer’s disease and bipolar disorder implicates the MARK2 and VAC14 genes. Front. Neurosci..

[21] Logue MW (2018). Targeted sequencing of Alzheimer disease genes in African Americans implicates novel risk variants. Front. Neurosci..

[22] Tollervey JR (2011). Analysis of alternative splicing associated with aging and neurodegeneration in the human brain. Genome Res..

[23] Wightman DP (2021). A genome-wide association study with 1,126,563 individuals identifies new risk loci for Alzheimer’s disease. Nat. Genet..

[24] Moreno-Grau S (2019). Genome-wide association analysis of dementia and its clinical endophenotypes reveal novel loci associated with Alzheimer’s disease and three causality networks: The GR@ACE project. Alzheimers. Dement..

[25] Satoh K, Suzuki N, Yokota H (2000). ADAMTS-4 (a disintegrin and metalloproteinase with thrombospondin motifs) is transcriptionally induced in beta-amyloid treated rat astrocytes. Neurosci. Lett..

[26] Tomita T (2020). Identification of ADAMTS4 as an APP-cleaving enzyme at 669 site in the APP669-711 production pathway. Alzheimers. Dement..

[27] Griciuc A (2019). TREM2 acts downstream of CD33 in modulating microglial pathology in Alzheimer’s disease. Neuron.

[28] White KE, Cummings JL (1996). Schizophrenia and Alzheimer’s disease: Clinical and pathophysiologic analogies. Compr. Psychiatry.

[29] Ropacki SA, Jeste DV (2005). Epidemiology of and risk factors for psychosis of Alzheimer’s disease: A review of 55 studies published from 1990 to 2003. Am. J. Psychiatry.

[30] Ribe AR (2015). Long-term risk of dementia in persons with schizophrenia: A Danish population-based cohort study. JAMA Psychiat..

[31] Creese B (2019). Examining the association between genetic liability for schizophrenia and psychotic symptoms in Alzheimer’s disease. Transl. Psychiatry.

[32] Sabia S (2021). Association of sleep duration in middle and old age with incidence of dementia. Nat. Commun..

[33] Schultz SA (2020). Association between personality and tau-PET binding in cognitively normal older adults. Brain Imaging Behav..

[34] Duberstein PR (2011). Personality and risk for Alzheimer’s disease in adults 72 years of age and older: A 6-year follow-up. Psychol. Aging.

[35] Aschenbrenner AJ (2020). Relationships between big-five personality factors and Alzheimer’s disease pathology in autosomal dominant Alzheimer's disease. Alzheimers. Dement..

[36] Dev K (2021). Prevalence of falls and fractures in Alzheimer’s patients compared to general population. Cureus.

[37] Murabito JM (2014). Parental longevity is associated with cognition and brain ageing in middle-aged offspring. Age Ageing.

[38] Lipton RB (2010). Exceptional parental longevity associated with lower risk of Alzheimer’s disease and memory decline. J. Am. Geriatr. Soc..

[39] Dehghani N, Bras J, Guerreiro R (2021). How understudied populations have contributed to our understanding of Alzheimer’s disease genetics. Brain.

[40] Bycroft C (2018). The UK Biobank resource with deep phenotyping and genomic data. Nature.

[41] Szustakowski JD (2020). Advancing human genetics research and drug discovery through exome sequencing of the UK Biobank. bioRxiv.

[42] Choi SW, O’Reilly PF (2019). PRSice-2: Polygenic risk score software for biobank-scale data. Gigascience.

[43] Chang CC (2015). Second-generation PLINK: Rising to the challenge of larger and richer datasets. Gigascience.

[44] Benjamini Y, Hochberg Y (1995). Controlling the false discovery rate: A practical and powerful approach to multiple testing. J. R. Stat. Soc..

[45] Bulik-Sullivan B (2015). An atlas of genetic correlations across human diseases and traits. Nat. Genet..

[46] Elsworth B (2020). The MRC IEU OpenGWAS data infrastructure. bioRxiv.

[47] Hemani G (2018). The MR-Base platform supports systematic causal inference across the human phenome. Elife.

[48] Millard LAC, Davies NM, Gaunt TR, Smith GD, Tilling K (2017). PHESANT: A tool for performing automated phenome scans in UK Biobank. Cold Spring Harb. Lab..

