# Supplementary material for: Genome-wide association of polygenic risk extremes for Alzheimer's disease in the UK Biobank
Source: Sci Rep. 2022 May 19;12:8404. doi: 10.1038/s41598-022-12391-2 (PMC9120074; doi:10.1038/s41598-022-12391-2)
Supplement: Supplementary file 1 — Supplementary Information 1. [file 41598_2022_12391_MOESM1_ESM.docx]

**Supplementary Materials**

**Genome-wide association of polygenic risk extremes for Alzheimer's disease in the UK Biobank**

**Table S1. Total 473 lead SNPs from PRS Extremes GWAS.**

| **Number** | **CHR** | **Position** | **Genomic Locus** | **Unique ID** | **RsID** | ***P*-value** | **# Independent Significant SNPs in LD** | **Independent Significant SNPs in LD** | **Nearest Gene** |
| --- | --- | --- | --- | --- | --- | --- | --- | --- | --- |
| **1** | 1 | 21122517 | 1 | 1:21122517:A:G | rs3927683 | 7.14E-09 | 1 | rs3927683 | *HP1BP3* |
| **2** | 1 | 21823292 | 1 | 1:21823292:C:T | rs1697421 | 6.42E-09 | 1 | rs1697421 | *NBPF3* |
| **3** | 1 | 26770054 | 2 | 1:26770054:C:CA | rs760794442 | 2.66E-08 | 1 | rs760794442 | *DHDDS* |
| **4** | 1 | 40087904 | 3 | 1:40087904:C:T | rs112751018 | 2.72E-09 | 2 | rs112751018; rs61781377 | *HEYL* |
| **5** | 1 | 46418900 | 4 | 1:46418900:T:TA | rs35599360 | 4.61E-10 | 3 | rs35599360; rs2993263; rs71062708 | *MAST2* |
| **6** | 1 | 50861622 | 5 | 1:50861622:C:T | rs7553439 | 2.71E-16 | 6 | rs7553439; rs72898937; rs12120719; rs35072907; rs35934575; rs12076343 | *HMGB1P45* |
| **7** | 1 | 78688673 | 6 | 1:78688673:G:T | rs11162433 | 7.27E-09 | 1 | rs11162433 | *RP11-183M13.1* |
| **8** | 1 | 85958322 | 7 | 1:85958322:C:T | rs551379 | 1.88E-14 | 4 | rs551379; rs511373; rs10493768; rs1146394 | *DDAH1* |
| **9** | 1 | 86460094 | 8 | 1:86460094:G:GAA | 1:86460094_GAA_G | 8.30E-14 | 3 | 1:86460094_GAA_G; rs2390015; rs12134342 | *COL24A1* |
| **10** | 1 | 103264228 | 9 | 1:103264228:C:T | rs61813880 | 1.04E-09 | 2 | rs61813880; rs560092711 | *RP5-936J12.1* |
| **11** | 1 | 108769358 | 10 | 1:108769358:C:T | rs371824699 | 5.71E-09 | 1 | rs371824699 | *NBPF4* |
| **12** | 1 | 150608653 | 11 | 1:150608653:C:G | rs72700870 | 8.31E-10 | 3 | rs72700870; rs6661135; rs28730724 | *ENSA* |
| **13** | 1 | 151382215 | 11 | 1:151382215:T:TTC | 1:151382215_TTC_T | 1.51E-11 | 2 | 1:151382215_TTC_T; rs28730724 | *POGZ* |
| **14** | 1 | 161155392 | 12 | 1:161155392:A:G | rs4575098 | 6.90E-18 | 7 | rs4575098; rs12094497; rs4379692; rs2070902; rs4489574; rs11579514; rs112810680 | *ADAMTS4* |
| **15** | 1 | 161500130 | 13 | 1:161500130:A:G | rs2099684 | 7.78E-09 | 1 | rs2099684 | *RP11-25K21.6* |
| **16** | 1 | 178997073 | 14 | 1:178997073:C:T | rs4652345 | 7.08E-09 | 1 | rs4652345 | *FAM20B* |
| **17** | 1 | 181597203 | 15 | 1:181597203:C:T | rs35821839 | 4.22E-08 | 1 | rs35821839 | *CACNA1E* |
| **18** | 1 | 190603308 | 16 | 1:190603308:A:T | rs35089183 | 4.33E-12 | 3 | rs35089183; rs1170914; rs12072609 | *RP11-463J7.2* |
| **19** | 1 | 191662234 | 17 | 1:191662234:C:G | rs76403827 | 1.28E-10 | 3 | rs76403827; rs79575666; rs72744340 | *RP11-541F9.1* |
| **20** | 1 | 207402224 | 18 | 1:207402224:C:T | rs6696130 | 3.30E-11 | 4 | rs6696130; rs11576522; rs61821016; rs34911917 | *C4BPAP2* |
| **21** | 1 | 207551690 | 18 | 1:207551690:A:G | rs77333453 | 1.59E-08 | 6 | rs77333453; rs4317805; rs17617; rs6690215; rs9429945; rs34911917 | *CD55* |
| **22** | 1 | 207773902 | 18 | 1:207773902:A:G | rs17259038 | 3.31E-10 | 5 | rs17259038; rs11576522; rs12073783; rs34626131; rs6690215 | *CR1:RP11-78B10.2* |
| **23** | 1 | 207802552 | 18 | 1:207802552:A:C | rs4844610 | 4.87E-25 | 11 | rs4844610; 1:207821519_AACAC_A; rs11118328; rs7537669; rs2761424; rs34911917; rs4317805; rs17617; rs6690215; rs9429945; rs11576522 | *CR1* |
| **24** | 1 | 207998783 | 18 | 1:207998783:C:G | rs7522307 | 7.55E-09 | 6 | rs7522307; rs1009897; rs11118328; rs7537669; rs2761424; rs575551889 | *C1orf132* |
| **25** | 1 | 208071561 | 18 | 1:208071561:G:T | rs2466570 | 2.57E-08 | 1 | rs2466570 | *CD34* |
| **26** | 1 | 213789584 | 19 | 1:213789584:A:G | rs77303682 | 2.46E-08 | 1 | rs77303682 | *AL592063.1* |
| **27** | 1 | 225549012 | 20 | 1:225549012:C:G | rs6673285 | 1.13E-09 | 2 | rs6673285; rs114562040 | *DNAH14* |
| **28** | 1 | 225737356 | 20 | 1:225737356:T:TTATG | 1:225737356_TTATG_T | 4.54E-08 | 2 | 1:225737356_TTATG_T; rs114562040 | *ENAH* |
| **29** | 1 | 226838508 | 21 | 1:226838508:G:T | rs16846346 | 2.24E-08 | 1 | rs16846346 | *ITPKB* |
| **30** | 2 | 26451699 | 22 | 2:26451699:C:T | rs7423500 | 2.14E-08 | 1 | rs7423500 | *HADHA* |
| **31** | 2 | 37543371 | 23 | 2:37543371:A:G | rs6544066 | 2.88E-08 | 1 | rs6544066 | *PRKD3* |
| **32** | 2 | 38269475 | 24 | 2:38269475:C:T | rs2121259 | 1.29E-08 | 1 | rs2121259 | *RMDN2:RMDN2-AS1* |
| **33** | 2 | 42200405 | 25 | 2:42200405:C:G | rs55981605 | 4.97E-08 | 1 | rs55981605 | *C2orf91* |
| **34** | 2 | 45497088 | 26 | 2:45497088:A:C | rs11884531 | 1.48E-08 | 1 | rs11884531 | *LINC01121* |
| **35** | 2 | 48107079 | 27 | 2:48107079:C:T | rs77969729 | 6.23E-11 | 6 | rs77969729; rs4583515; rs13422058; rs77134676; rs111588601; rs74533980 | *FBXO11* |
| **36** | 2 | 48476659 | 27 | 2:48476659:C:T | rs2221714 | 2.51E-08 | 4 | rs2221714; rs77134676; rs4583515; rs13422058 | *AC079807.4* |
| **37** | 2 | 50353870 | 28 | 2:50353870:G:T | rs115961570 | 1.28E-08 | 2 | rs115961570; rs1915222 | *NRXN1* |
| **38** | 2 | 57669304 | 29 | 2:57669304:C:T | rs17048826 | 9.66E-09 | 1 | rs17048826 | *RP11-127H13.1* |
| **39** | 2 | 57757195 | 29 | 2:57757195:C:G | rs75939231 | 8.00E-09 | 4 | rs75939231; rs13386150; rs147804554; rs77272075 | *SNORD78* |
| **40** | 2 | 57891748 | 29 | 2:57891748:C:CT | rs59296552 | 2.28E-09 | 4 | rs59296552; rs77272075; rs13386150; rs1460259 | *CTD-2026C7.1* |
| **41** | 2 | 63676024 | 30 | 2:63676024:C:T | rs72821619 | 3.82E-09 | 3 | rs72821619; rs11684093; rs4671451 | *WDPCP* |
| **42** | 2 | 65019859 | 31 | 2:65019859:A:T | rs13033563 | 2.72E-09 | 1 | rs13033563 | *AC007365.2* |
| **43** | 2 | 65053188 | 31 | 2:65053188:A:G | rs62137344 | 7.43E-19 | 7 | rs62137344; rs12373586; rs12475560; rs116224672; rs62649819; rs55819441; rs62137339 | *Y_RNA* |
| **44** | 2 | 81644012 | 32 | 2:81644012:C:CAT | 2:81644012_CAT_C | 1.94E-08 | 2 | 2:81644012_CAT_C; rs114161609 | *AC012075.1* |
| **45** | 2 | 84478792 | 33 | 2:84478792:A:G | rs77470225 | 2.30E-08 | 1 | rs77470225 | *FUNDC2P2* |
| **46** | 2 | 95406277 | 34 | 2:95406277:A:C | rs144417606 | 4.47E-08 | 1 | rs144417606 | *CNN2P8* |
| **47** | 2 | 118182617 | 35 | 2:118182617:A:T | rs12711879 | 2.67E-10 | 1 | rs12711879 | *AC092170.1* |
| **48** | 2 | 127830219 | 36 | 2:127830219:A:C | rs17014873 | 3.50E-10 | 2 | rs17014873; rs1060743 | *BIN1* |
| **49** | 2 | 127838767 | 36 | 2:127838767:C:T | rs12329015 | 1.20E-08 | 4 | rs12329015; rs10194375; rs13032148; rs140982764 | *BIN1* |
| **50** | 2 | 127891427 | 36 | 2:127891427:A:C | rs4663105 | 1.57E-24 | 8 | rs4663105; rs744373; rs1060743; rs10194375; rs1530047; rs13032148; rs140982764; rs35103166 | *BIN1* |
| **51** | 2 | 135372951 | 37 | 2:135372951:A:G | rs35564151 | 3.51E-09 | 2 | rs35564151; rs6760237 | *TMEM163* |
| **52** | 2 | 155668076 | 38 | 2:155668076:C:T | rs34412678 | 7.48E-13 | 7 | rs34412678; rs70983746; rs138190655; rs12616498; rs78838860; rs4464226; rs1445658 | *KCNJ3* |
| **53** | 2 | 156469012 | 38 | 2:156469012:A:T | rs707122 | 1.39E-10 | 7 | rs707122; rs4464226; rs67354756; rs1445658; rs138190655; rs12616498; rs78838860 | *RNU6-546P* |
| **54** | 2 | 160693784 | 39 | 2:160693784:A:G | rs75630252 | 3.09E-13 | 3 | rs75630252; 2:160760628_CG_C; rs56345947 | *LY75:LY75-CD302* |
| **55** | 2 | 160870601 | 39 | 2:160870601:C:T | rs1039931 | 1.30E-08 | 2 | rs1039931; 2:160760628_CG_C | *PLA2R1* |
| **56** | 2 | 161777866 | 40 | 2:161777866:G:T | rs13002188 | 2.17E-09 | 2 | rs13002188; rs75041611 | *RN7SL423P* |
| **57** | 2 | 161894663 | 40 | 2:161894663:A:G | rs197273 | 1.64E-10 | 2 | rs197273; rs75041611 | *AC009313.2* |
| **58** | 2 | 161931112 | 40 | 2:161931112:A:G | rs13013777 | 4.36E-09 | 1 | rs13013777 | *AC009313.1* |
| **59** | 2 | 174104705 | 41 | 2:174104705:C:G | rs16861417 | 1.29E-08 | 1 | rs16861417 | *MLTK:MLK7-AS1* |
| **60** | 2 | 185412942 | 42 | 2:185412942:A:ATGTT | rs745394265 | 2.09E-08 | 1 | rs745394265 | *ZNF804A* |
| **61** | 2 | 186746683 | 43 | 2:186746683:C:G | rs114793336 | 2.54E-08 | 1 | rs114793336 | *FSIP2* |
| **62** | 2 | 187798788 | 44 | 2:187798788:G:T | rs16828514 | 5.06E-12 | 1 | rs16828514 | *AC018735.1* |
| **63** | 2 | 188162128 | 44 | 2:188162128:C:T | rs17705798 | 2.53E-11 | 4 | rs17705798; rs10931283; rs2192824; rs2349653 | *AC007319.1* |
| **64** | 2 | 203649501 | 45 | 2:203649501:C:G | rs72932727 | 6.95E-15 | 9 | rs72932727; rs539997380; rs377724024; 2:204298556_AT_A; rs62182788; rs139644567; rs6435128; rs7581542; rs11675462 | *ICA1L* |
| **65** | 2 | 234067884 | 46 | 2:234067884:A:G | rs36133610 | 4.78E-14 | 7 | rs36133610; rs6741388; rs28615641; rs7599330; rs10933431; rs10193128; rs36134067 | *INPP5D* |
| **66** | 3 | 41678906 | 47 | 3:41678906:C:T | rs60659132 | 1.81E-08 | 1 | rs60659132 | *ULK4* |
| **67** | 3 | 48710739 | 48 | 3:48710739:C:T | rs75654367 | 3.53E-09 | 2 | rs75654367; rs188146975 | *NCKIPSD* |
| **68** | 3 | 48854758 | 48 | 3:48854758:C:CAATAAATAAATA | 3:48854758_CAATAAATAAATA_C | 3.73E-13 | 1 | 3:48854758_CAATAAATAAATA_C | *PRKAR2A* |
| **69** | 3 | 49738816 | 48 | 3:49738816:A:G | rs79581555 | 1.26E-12 | 5 | rs79581555; rs10865959; rs111873781; rs201175105; rs188146975 | *RNF123* |
| **70** | 3 | 50204745 | 48 | 3:50204745:C:T | rs3774745 | 3.85E-21 | 15 | rs3774745; rs2236941; rs56048629; rs4547694; rs12637870; rs6446296; rs2247036; rs10865959; rs2280405; rs11712056; rs139738301; rs2014830; rs2624847; rs148149389; rs6800021 | *SEMA3F* |
| **71** | 3 | 50444044 | 48 | 3:50444044:C:CCAT | 3:50444044_CCAT_C | 1.28E-13 | 15 | 3:50444044_CCAT_C; rs12637870; rs916288; rs73084740; rs665912; rs73072483; rs62261860; rs532467707; rs6446296; rs11712056; rs2014830; rs148149389; rs2236941; rs56048629; rs4547694 | *CACNA2D2* |
| **72** | 3 | 50960634 | 48 | 3:50960634:A:G | rs116771349 | 1.51E-10 | 5 | rs116771349; rs17606778; rs916288; rs665912; rs62261860 | *DOCK3* |
| **73** | 3 | 51821181 | 48 | 3:51821181:C:T | rs113896921 | 4.84E-08 | 2 | rs113896921; rs62261860 | *IQCF6* |
| **74** | 3 | 51904546 | 48 | 3:51904546:A:G | rs13079734 | 6.05E-12 | 2 | rs13079734; rs73080683 | *RN7SL504P* |
| **75** | 3 | 55952415 | 49 | 3:55952415:G:T | rs1379725 | 1.97E-08 | 1 | rs1379725 | *ERC2* |
| **76** | 3 | 57735817 | 50 | 3:57735817:C:CT | rs79762933 | 1.08E-09 | 2 | rs79762933; rs9815266 | *SLMAP* |
| **77** | 3 | 71849696 | 51 | 3:71849696:A:G | rs9310221 | 1.11E-08 | 2 | rs9310221; rs13080898 | *PROK2* |
| **78** | 3 | 79406645 | 52 | 3:79406645:A:T | rs12639058 | 3.29E-13 | 2 | rs12639058; rs58082488 | *ROBO1* |
| **79** | 3 | 84203218 | 53 | 3:84203218:C:T | rs62258410 | 4.24E-11 | 8 | rs62258410; rs4856493; rs6788354; rs57622049; rs62255666; rs9875852; rs7622223; rs4475054 | *AC108696.1* |
| **80** | 3 | 84464459 | 53 | 3:84464459:G:GA | 3:84464459_GA_G | 7.41E-16 | 13 | 3:84464459_GA_G; rs62255755; rs62255756; rs7622685; rs2924660; rs9844514; rs7622223; rs4475054; rs4856493; rs6788354; rs57622049; rs62255666; rs9875852AC107025.1 |  |
| **81** | 3 | 84765690 | 53 | 3:84765690:C:G | rs62255507 | 1.09E-08 | 1 | rs62255507 | LINC00971 |
| **82** | 3 | 85142871 | 53 | 3:85142871:A:G | rs6798922 | 1.70E-08 | 1 | rs6798922 | CADM2 |
| **83** | 3 | 90296337 | 54 | 3:90296337:A:T | rs34803068 | 2.53E-08 | 1 | rs34803068 | PROSP |
| **84** | 3 | 103783616 | 55 | 3:103783616:C:T | rs9870095 | 1.04E-10 | 2 | rs9870095; rs9849114 | RP11-40M23.1 |
| **85** | 3 | 116515533 | 56 | 3:116515533:A:G | rs13063609 | 1.01E-08 | 1 | rs13063609 | LSAMP |
| **86** | 3 | 135798396 | 57 | 3:135798396:A:G | rs6771887 | 4.11E-10 | 4 | rs6771887; 3:136397692_CA_C; rs111919234; rs12632781 | PPP2R3A |
| **87** | 3 | 153207195 | 58 | 3:153207195:A:C | rs11716593 | 2.26E-09 | 1 | rs11716593 | RP11-23D24.2:C3orf79 |
| **88** | 3 | 155356743 | 59 | 3:155356743:C:T | rs9844708 | 3.54E-15 | 10 | rs9844708; rs6769807; rs358908; rs358726; rs62276955; rs9826383; rs359542; rs763699141; rs201534447; rs201534447 | PLCH1 |
| **89** | 3 | 162802753 | 60 | 3:162802753:A:G | rs7427962 | 2.01E-08 | 1 | rs7427962 | RP11-10O22.1 |
| **90** | 3 | 163546423 | 61 | 3:163546423:A:G | rs1907860 | 1.98E-10 | 2 | rs1907860; rs6774925 | RP11-208P4.1 |
| **91** | 3 | 195937316 | 62 | 3:195937316:A:G | rs555882585 | 9.67E-09 | 1 | rs555882585 | ZDHHC19 |
| **92** | 4 | 19395137 | 63 | 4:19395137:C:T | rs968264 | 1.66E-08 | 1 | rs968264 | RP11-3J1.1 |
| **93** | 4 | 46916149 | 64 | 4:46916149:A:C | rs62303695 | 1.09E-08 | 2 | rs62303695; rs62305175 | GABRA4 |
| **94** | 4 | 61925972 | 65 | 4:61925972:A:G | rs17828156 | 5.61E-10 | 2 | rs17828156; rs55950739 | RP11-16N2.1 |
| **95** | 4 | 68808754 | 66 | 4:68808754:A:AG | 4:68808754_AG_A | 1.41E-08 | 1 | 4:68808754_AG_A | UBA6-AS1:TMPRSS11A |
| **96** | 4 | 71113479 | 67 | 4:71113479:C:CT | 4:71113479_CT_C | 3.78E-10 | 1 | 4:71113479_CT_C | CSN3 |
| **97** | 4 | 78944944 | 68 | 4:78944944:A:G | rs10002506 | 2.12E-08 | 1 | rs10002506 | RP11-777B9.5 |
| **98** | 4 | 86877952 | 69 | 4:86877952:A:C | rs3943730 | 1.95E-11 | 1 | rs3943730 | ARHGAP24 |
| **99** | 4 | 99813832 | 70 | 4:99813832:C:CAA | 4:99813832_CAA_C | 3.11E-10 | 1 | 4:99813832_CAA_C | EIF4E |
| **100** | 4 | 119227259 | 71 | 4:119227259:C:T | rs71608363 | 7.44E-09 | 1 | rs71608363 | PRSS12 |
| **101** | 4 | 151761599 | 72 | 4:151761599:A:G | rs6846361 | 4.29E-10 | 2 | rs6846361; 4:151428647_TA_T | LRBA |
| **102** | 4 | 152273295 | 73 | 4:152273295:C:T | rs2897617 | 1.81E-09 | 1 | rs2897617 | RP11-731D1.4 |
| **103** | 4 | 159857539 | 74 | 4:159857539:A:G | rs11100203 | 1.92E-12 | 1 | rs11100203 | C4orf45 |
| **104** | 4 | 160043026 | 74 | 4:160043026:C:CA | rs368123758 | 3.66E-08 | 1 | rs368123758 | RAPGEF2 |
| **105** | 4 | 160218812 | 74 | 4:160218812:A:AC | rs111559755 | 2.50E-08 | 1 | rs111559755 | RAPGEF2 |
| **106** | 5 | 4096918 | 75 | 5:4096918:C:T | rs62334698 | 1.98E-08 | 1 | rs62334698 | CTD-2008N3.1 |
| **107** | 5 | 41428484 | 76 | 5:41428484:G:T | rs7701537 | 1.25E-08 | 1 | rs7701537 | PLCXD3 |
| **108** | 5 | 42576185 | 77 | 5:42576185:A:G | rs144429807 | 4.38E-14 | 4 | rs144429807; rs13173465; rs35948016; rs75796515 | GHR |
| **109** | 5 | 43097074 | 77 | 5:43097074:A:G | rs316411 | 3.14E-10 | 3 | rs316411; rs315262; 5:43096083_TG_T | ZNF131 |
| **110** | 5 | 43537902 | 78 | 5:43537902:C:T | rs139849934 | 3.18E-10 | 2 | rs139849934; rs62368104 | PAIP1 |
| **111** | 5 | 45333860 | 79 | 5:45333860:C:T | rs55821517 | 4.02E-13 | 8 | rs55821517; rs3028840; rs11948152; rs10043248; rs994793; rs16902086; rs11740651; rs148415520 | HCN1 |
| **112** | 5 | 49903952 | 80 | 5:49903952:A:G | rs75570187 | 1.89E-09 | 3 | rs75570187; rs533835034; rs11950897 | RP11-269M20.2 |
| **113** | 5 | 60098267 | 81 | 5:60098267:A:G | rs4700393 | 5.78E-10 | 3 | rs4700393; rs112375441; rs12188710 | ELOVL7 |
| **114** | 5 | 60795927 | 81 | 5:60795927:T:TTTATTA | rs372083232 | 3.78E-08 | 3 | rs372083232; rs12188710; rs112375441 | ZSWIM6 |
| **115** | 5 | 71657414 | 82 | 5:71657414:A:G | rs62364788 | 1.60E-09 | 1 | rs62364788 | CTC-365E16.1 |
| **116** | 5 | 71751379 | 82 | 5:71751379:C:T | rs10073367 | 5.01E-09 | 2 | rs10073367; rs9293820 | ZNF366 |
| **117** | 5 | 86295135 | 83 | 5:86295135:A:G | rs72779757 | 2.28E-08 | 1 | rs72779757 | RP11-72L22.1 |
| **118** | 5 | 86298531 | 83 | 5:86298531:C:T | rs62375397 | 3.22E-09 | 1 | rs62375397 | RP11-72L22.1 |
| **119** | 5 | 98447717 | 84 | 5:98447717:C:T | rs12054753 | 1.12E-08 | 1 | rs12054753 | CTD-2007H13.3 |
| **120** | 5 | 98928283 | 84 | 5:98928283:C:CTAT | 5:98928283_CTAT_C | 3.21E-09 | 1 | 5:98928283_CTAT_C | CTD-2151A2.2 |
| **121** | 5 | 101208708 | 85 | 5:101208708:C:T | rs36054210 | 4.58E-10 | 1 | rs36054210 | OR7H2P |
| **122** | 5 | 105786697 | 86 | 5:105786697:A:ATTTAT | rs376016059 | 1.73E-09 | 1 | rs376016059 | CTC-278L1.1 |
| **123** | 5 | 129637007 | 87 | 5:129637007:A:G | rs76826150 | 2.38E-08 | 5 | rs76826150; rs80352688; rs7728037; rs139065728; 5:130219075_CT_C | AC004769.1 |
| **124** | 5 | 129873800 | 87 | 5:129873800:G:T | rs10077003 | 1.71E-14 | 12 | rs10077003; rs139065728; 5:130143319_AT_A; rs9687513; rs6867080; rs4492106; 5:130219075_CT_C; rs4705998; rs7356548; rs80352688; rs7728037; rs11242026 | ARL2BPP4 |
| **125** | 5 | 138055308 | 88 | 5:138055308:C:T | rs17286171 | 2.79E-09 | 1 | rs17286171 | CTNNA1 |
| **126** | 5 | 138199901 | 88 | 5:138199901:A:G | rs114901155 | 3.26E-08 | 1 | rs114901155 | CTNNA1 |
| **127** | 5 | 139665181 | 89 | 5:139665181:C:T | rs7715538 | 1.00E-11 | 3 | rs7715538; rs11168036; rs11445186 | PFDN1 |
| **128** | 5 | 139741370 | 89 | 5:139741370:A:C | rs717097 | 1.33E-20 | 14 | rs717097; rs111998037; rs62385221; rs1962649; rs6899256; rs13168533; rs13174119; rs782706309; rs246069; rs246039; rs31871; rs72796764; rs11445186; rs11168036 | SLC4A9 |
| **129** | 5 | 140739874 | 89 | 5:140739874:A:G | rs13171859 | 4.11E-10 | 3 | rs13171859; rs13168533; rs116483435 | PCDHGA1:PCDHGA2:PCDHGA3:PCDHGB1:PCDHGA4:PCDHGB2 |
| **130** | 5 | 144884016 | 90 | 5:144884016:A:T | rs4912696 | 1.03E-08 | 1 | rs4912696 | PRELID2 |
| **131** | 5 | 152606214 | 91 | 5:152606214:A:G | rs72793631 | 3.71E-09 | 1 | rs72793631 | AC091969.1 |
| **132** | 5 | 153246661 | 92 | 5:153246661:C:T | rs7712317 | 1.54E-09 | 3 | rs7712317; 5:153271974_CA_C; rs74798625 | AC091962.3 |
| **133** | 5 | 153687608 | 93 | 5:153687608:C:CT | 5:153687608_CT_C | 8.65E-13 | 2 | 5:153687608_CT_C; rs11740474 | GALNT10 |
| **134** | 5 | 174940236 | 94 | 5:174940236:A:G | rs2644669 | 2.30E-08 | 1 | rs2644669 | SFXN1 |
| **135** | 6 | 25789199 | 95 | 6:25789199:A:ATAGG | rs139537655 | 1.20E-10 | 13 | rs139537655; rs13210399; rs9393686; rs1417998; rs35162296; 6:26318745_CA_C; rs9467711; rs9467714; rs9393711; rs9348729; rs4236040; rs112613250; rs9467596 | SLC17A1 |
| **136** | 6 | 26122933 | 95 | 6:26122933:G:T | rs198823 | 4.66E-09 | 3 | rs198823; rs626824; rs169219 | HIST1H2BC |
| **137** | 6 | 26572165 | 95 | 6:26572165:G:T | rs6925895 | 1.17E-08 | 12 | rs6925895; rs9348729; rs147951948; rs138369630; rs10946859; rs10946899; 6:27033595_TTATATATATATATATA_T; rs7764984; rs911186; rs2295603; rs9393801; rs34565965 | ABT1 |
| **138** | 6 | 26907831 | 95 | 6:26907831:C:T | rs9379945 | 5.19E-18 | 29 | rs9379945; rs10946899; 6:27033595_TTATATATATATATATA_T; rs7764984; rs911186; rs2295603; rs9393801; 6:27276194_GA_G; rs6923811; rs6903160; rs35984974; rs113039233; rs2294480; rs12179134; rs573179; rs200972; rs13210399; rs9393686; rs1417998; rs35162296; 6:26318745_CA_C; rs9467711; rs9467714; rs34565965; rs9393711; rs9348729; rs147951948; rs138369630; rs10946859 | GUSBP2 |
| **139** | 6 | 27189171 | 95 | 6:27189171:T:TG | rs148051045 | 7.39E-11 | 3 | rs148051045; rs11754427; rs1150697 | RP11-209A2.1 |
| **140** | 6 | 27303745 | 95 | 6:27303745:A:T | rs9368503 | 9.23E-11 | 16 | rs9368503; rs6903160; rs113039233; 6:27468192_GGA_G; rs2294480; rs12179134; rs573179; rs200972; 6:28110307_GT_G; rs34565965; rs147951948; rs10946899; rs7764984; rs550539952; rs2295603; 6:27276194_GA_G | VN1R10P |
| **141** | 6 | 27633772 | 95 | 6:27633772:T:TAC | rs35959996 | 2.19E-12 | 2 | rs35959996; rs2294480 | RP1-15D7.1 |
| **142** | 6 | 27886830 | 95 | 6:27886830:C:T | rs2130357 | 3.93E-09 | 18 | rs2130357; rs9295753; 6:28110307_GT_G; rs1150697; rs11431027; rs12000; rs1778482; rs200096835; rs7772827; rs13201753; rs2531805; rs442439; rs9393921; 6:28680539_TTA_T; rs112892668; rs2294480; rs573179; rs200972 | OR2B2 |
| **143** | 6 | 28750876 | 95 | 6:28750876:T:TCAAAA | rs200690674 | 8.67E-20 | 18 | rs200690674; rs150983563; rs60569033; rs9468477; rs374588508; rs9257703; rs720831; rs1052477; 6:29533581_AATAG_A; rs9295753; rs1150697; rs12000; rs200096835; rs9393921; 6:28680539_TTA_T; rs373203454; rs1233597; rs112892668 | NOL5BP |
| **144** | 6 | 28790373 | 95 | 6:28790373:C:CT | rs146924495 | 3.66E-26 | 33 | rs146924495; rs3132379; rs60569033; rs3118361; rs9468477; rs377436390; rs374588508; rs9257703; rs720831; rs2021078; rs1052477; rs926552; rs3095268; rs573179; rs200972; 6:28110307_GT_G; rs11431027; rs9393909; rs1778482; rs17312661; rs7772827; rs13201753; rs2531805; rs6902687; rs442439; 6:28640498_CG_C; rs9393921; rs780222578; rs7775835; 6:28680539_TTA_T; rs373203454; rs1233597; rs150983563 | XXbac-BPG308K3.5 |
| **145** | 6 | 29379304 | 95 | 6:29379304:C:G | rs3117190 | 3.58E-18 | 7 | rs3117190; rs3094574; 6:29535335_TA_T; rs118158289; 6:28680539_TTA_T; rs9257270; rs3094549 | OR5V1 |
| **146** | 6 | 29545208 | 95 | 6:29545208:C:T | rs362522 | 8.70E-09 | 1 | rs362522 | GABBR1 |
| **147** | 6 | 29559046 | 95 | 6:29559046:A:AATAATC | rs148032752 | 1.74E-12 | 10 | rs148032752; rs3095268; rs373203454; rs112892668; rs150983563; rs60569033; rs9468477; rs374588508; rs1052477; 6:29533581_AATAG_A | GABBR1 |
| **148** | 6 | 29604264 | 95 | 6:29604264:G:T | rs9461540 | 4.62E-18 | 3 | rs9461540; 6:29584476_AAG_A; rs118158289 | SUMO2P1 |
| **149** | 6 | 29610435 | 95 | 6:29610435:C:T | rs3129077 | 5.56E-11 | 4 | rs3129077; rs9257703; rs926552; rs3095268 | SUMO2P1 |
| **150** | 6 | 34651306 | 96 | 6:34651306:C:T | rs111868030 | 4.19E-08 | 2 | rs111868030; rs59136961 | C6orf106 |
| **151** | 6 | 41140984 | 97 | 6:41140984:C:G | rs9394764 | 1.01E-09 | 2 | rs9394764; rs3857580 | TREM2 |
| **152** | 6 | 41222926 | 97 | 6:41222926:C:T | rs1872245 | 1.62E-08 | 2 | rs1872245; rs3857580 | TREML5P |
| **153** | 6 | 45706189 | 98 | 6:45706189:A:G | rs74960871 | 1.44E-08 | 1 | rs74960871 | RUNX2 |
| **154** | 6 | 47595155 | 99 | 6:47595155:A:T | rs1385742 | 7.24E-13 | 3 | rs1385742; rs9395288; rs1928464 | CD2AP |
| **155** | 6 | 49158542 | 100 | 6:49158542:A:G | rs2516118 | 1.46E-11 | 3 | rs2516118; rs9369814; rs6940183 | RP1-142O9.2 |
| **156** | 6 | 51178405 | 101 | 6:51178405:C:T | rs2504672 | 7.93E-10 | 1 | rs2504672 | RP3-437C15.2 |
| **157** | 6 | 56311121 | 102 | 6:56311121:A:C | rs2397225 | 8.89E-10 | 1 | rs2397225 | DST |
| **158** | 6 | 57121684 | 103 | 6:57121684:A:G | rs6904307 | 6.18E-21 | 8 | rs6904307; rs34593037; rs9475820; rs34633842; rs572834430; rs143346424; rs77450031; rs6459181 | RAB23 |
| **159** | 6 | 57546745 | 103 | 6:57546745:A:G | rs2498215 | 4.76E-08 | 5 | rs2498215; rs10155788; rs10949211; rs12202889; rs9370723 | PRIM2 |
| **160** | 6 | 57603337 | 103 | 6:57603337:A:G | rs115274881 | 7.82E-15 | 5 | rs115274881; rs10155788; rs573809; rs1557219; rs139543045 | GAPDHP41 |
| **161** | 6 | 57922673 | 103 | 6:57922673:A:T | rs6916215 | 3.28E-16 | 12 | rs6916215; rs10949211; rs12202889; rs572834430; rs9370723; rs143346424; 6:58222820_ATTGATTAAAG_A; rs60186309; rs62464139; rs115150765; rs28621940; rs10155788 | RBBP4P3 |
| **162** | 6 | 58247096 | 103 | 6:58247096:C:G | rs2982041 | 9.31E-12 | 6 | rs2982041; rs573809; rs60186309; rs1557219; rs757597048; rs1570080 | GUSBP4 |
| **163** | 6 | 58677437 | 103 | 6:58677437:A:T | rs2693062 | 1.08E-27 | 10 | rs2693062; rs4928486; rs10155788; rs12202889; 6:58222820_ATTGATTAAAG_A; rs573809; rs60186309; rs1557219; rs62464139; rs757597048 | RP11-143A22.1 |
| **164** | 6 | 62101394 | 104 | 6:62101394:A:C | rs62425025 | 3.86E-22 | 17 | rs62425025; rs369158727; rs2880772; rs113702905; rs9453572; rs201982583; rs71552589; rs62416677; rs1192425; rs1553392; rs9445769; rs12202047; rs176623; rs79923126; rs12190096; rs1577635; rs9500740 | AL356131.1 |
| **165** | 6 | 62578251 | 104 | 6:62578251:A:C | rs1575241 | 9.79E-10 | 8 | rs1575241; rs201982583; rs71552589; rs1192425; rs12190096; rs1577635; rs9500740; rs113702905 | KHDRBS2 |
| **166** | 6 | 62789334 | 104 | 6:62789334:G:T | rs77744132 | 3.93E-10 | 10 | rs77744132; rs12202047; rs10943188; rs504893; rs201361160; rs10455336; rs79923126; rs2880772; rs62416677; rs9445769 | KHDRBS2 |
| **167** | 6 | 63162857 | 104 | 6:63162857:C:T | rs9360446 | 2.35E-22 | 20 | rs9360446; rs2007007; rs9446709; rs66880871; rs2474879; rs504893; rs4084782; rs4452620; rs12205136; rs4710399; rs789942; rs113702905; rs1553392; rs9445769; rs772554998; rs9354628; rs176623; rs563889625; rs4710680; rs17380739 | RP11-448N11.1 |
| **168** | 6 | 63303929 | 104 | 6:63303929:C:T | rs56201047 | 8.57E-09 | 6 | rs56201047; rs66880871; rs12205136; rs9354628; rs17380739; rs2007007 | RP11-448N11.3 |
| **169** | 6 | 63820771 | 104 | 6:63820771:A:AC | 6:63820771_AC_A | 2.13E-20 | 19 | 6:63820771_AC_A; rs34010334; rs9361410; rs9352692; rs9341766; rs9352774; rs12202047; rs4710680; rs2007007; rs9446709; rs2474879; rs10943188; rs504893; rs4452620; rs12194799; rs4710399; rs201361160; rs10455336; rs789942 | RP11-184C23.1 |
| **170** | 6 | 64353020 | 104 | 6:64353020:A:G | rs1681939 | 9.56E-11 | 4 | rs1681939; rs201361160; rs2500459; rs9352774 | PHF3 |
| **171** | 6 | 67689107 | 105 | 6:67689107:A:G | rs6916381 | 1.01E-08 | 1 | rs6916381 | AL590874.1 |
| **172** | 6 | 68282499 | 106 | 6:68282499:A:T | rs187831418 | 5.56E-09 | 1 | rs187831418 | RNU6-280P |
| **173** | 6 | 68542224 | 106 | 6:68542224:A:G | rs34964601 | 2.66E-08 | 1 | rs34964601 | RP11-301G19.1 |
| **174** | 6 | 68874442 | 107 | 6:68874442:A:G | rs72904115 | 1.21E-09 | 1 | rs72904115 | RP11-406O16.1 |
| **175** | 6 | 73079004 | 108 | 6:73079004:A:G | rs12528135 | 5.58E-12 | 2 | rs12528135; rs12526116 | RIMS1 |
| **176** | 6 | 79289552 | 109 | 6:79289552:C:T | rs603964 | 1.49E-08 | 1 | rs603964 | RP3-390M24.1 |
| **177** | 6 | 79486518 | 109 | 6:79486518:C:T | rs9448565 | 3.54E-08 | 1 | rs9448565 | RP11-173D14.3 |
| **178** | 6 | 85997747 | 110 | 6:85997747:A:G | rs72918351 | 1.83E-08 | 1 | rs72918351 | RP11-30P6.1:KRT18P64 |
| **179** | 6 | 98199541 | 111 | 6:98199541:C:G | rs9401134 | 7.88E-09 | 1 | rs9401134 | RP1-104O17.2 |
| **180** | 6 | 109736614 | 112 | 6:109736614:A:G | rs36035949 | 4.02E-13 | 2 | rs36035949; rs7451512 | PPIL6 |
| **181** | 6 | 114033026 | 113 | 6:114033026:A:G | rs114464971 | 6.72E-09 | 1 | rs114464971 | AL357519.1 |
| **182** | 6 | 117240980 | 114 | 6:117240980:C:T | rs1040775 | 4.91E-09 | 1 | rs1040775 | RFX6 |
| **183** | 6 | 149891670 | 115 | 6:149891670:C:CAA | 6:149891670_CAA_C | 9.83E-09 | 1 | 6:149891670_CAA_C | GINM1 |
| **184** | 6 | 156618923 | 116 | 6:156618923:C:T | rs11759982 | 3.78E-09 | 1 | rs11759982 | SNORD28 |
| **185** | 7 | 3511493 | 117 | 7:3511493:A:G | rs73671840 | 5.05E-10 | 3 | rs73671840; rs73673633; rs201048917 | SDK1 |
| **186** | 7 | 6423066 | 118 | 7:6423066:T:TTAAAG | rs10631987 | 7.86E-09 | 1 | rs10631987 | RAC1 |
| **187** | 7 | 12579007 | 119 | 7:12579007:A:G | rs10224286 | 1.01E-09 | 2 | rs10224286; rs11773705 | AC005281.1 |
| **188** | 7 | 29536292 | 120 | 7:29536292:A:T | rs10268281 | 8.59E-09 | 1 | rs10268281 | CHN2 |
| **189** | 7 | 32586201 | 121 | 7:32586201:A:G | rs28434291 | 2.01E-11 | 2 | rs28434291; 7:32764412_AGACAGG_A | AVL9 |
| **190** | 7 | 37619144 | 122 | 7:37619144:C:T | rs10270490 | 1.38E-08 | 1 | rs10270490 | NECAP1P1 |
| **191** | 7 | 49366759 | 123 | 7:49366759:G:T | rs2116020 | 1.33E-08 | 1 | rs2116020 | RP11-122G11.1 |
| **192** | 7 | 63953995 | 124 | 7:63953995:T:TTA | rs71055224 | 3.08E-11 | 4 | rs71055224; 7:63611742_AT_A; rs6460131; rs547177516 | HNRNPCP7 |
| **193** | 7 | 63973141 | 124 | 7:63973141:C:CT | 7:63973141_CT_C | 2.05E-10 | 6 | 7:63973141_CT_C; rs62455850; rs74622139; rs12539512; 7:64424542_TA_T; rs112928221 | ZNF680 |
| **194** | 7 | 64478008 | 124 | 7:64478008:G:T | rs561635564 | 2.14E-08 | 1 | rs561635564 | RNU6-1229P |
| **195** | 7 | 65016198 | 124 | 7:65016198:A:G | rs201110880 | 1.43E-09 | 6 | rs201110880; rs62455850; rs74622139; rs12539512; 7:64424542_TA_T; rs112928221 | RP11-667F9.1 |
| **196** | 7 | 66130139 | 124 | 7:66130139:G:GT | 7:66130139_GT_G | 1.12E-09 | 4 | 7:66130139_GT_G; rs7782418; 7:66563942_AT_A; rs28446205 | KCTD7:RP4-756H11.3 |
| **197** | 7 | 66482730 | 124 | 7:66482730:C:T | rs117207241 | 3.74E-09 | 1 | rs117207241 | TYW1 |
| **198** | 7 | 66838377 | 124 | 7:66838377:C:T | rs6957404 | 7.85E-09 | 1 | rs6957404 | AC006480.1 |
| **199** | 7 | 69104712 | 125 | 7:69104712:C:T | rs73167509 | 1.43E-08 | 1 | rs73167509 | AUTS2 |
| **200** | 7 | 75766804 | 126 | 7:75766804:A:G | rs10255318 | 5.62E-09 | 1 | rs10255318 | AC005077.7 |
| **201** | 7 | 95228460 | 127 | 7:95228460:C:T | rs12671532 | 3.75E-08 | 1 | rs12671532 | AC002451.3 |
| **202** | 7 | 98817797 | 128 | 7:98817797:A:T | rs9886136 | 1.87E-08 | 1 | rs9886136 | KPNA7 |
| **203** | 7 | 99471072 | 128 | 7:99471072:C:T | rs2099446 | 6.95E-16 | 13 | rs2099446; rs35609229; rs1981550; rs2525548; rs2527884; rs10274982; rs6465759; rs71569528; rs11771331; rs12531809; rs2950520; rs34857299; rs35305377 | CYP3A52P |
| **204** | 7 | 99739219 | 128 | 7:99739219:C:T | rs76483787 | 4.01E-14 | 9 | rs76483787; rs2950520; rs34857299; rs2906644; rs2037595; rs35609229; rs1981550; rs71569528; rs11771331 | RPL7P60 |
| **205** | 7 | 99777869 | 129 | 7:99777869:A:C | rs7457787 | 8.21E-09 | 4 | rs7457787; rs858502; rs35305377; rs6465759 | STAG3 |
| **206** | 7 | 99950006 | 129 | 7:99950006:C:T | rs13246354 | 1.07E-18 | 13 | rs13246354; rs113027130; rs73405382; rs221773; rs77220350; rs10274982; rs12531809; rs111255152; rs113683483; rs12705074; rs35887778; rs858502; rs35305377 | STAG3L5P-PVRIG2P-PILRB:PILRB:PVRIG2P |
| **207** | 7 | 99984089 | 129 | 7:99984089:C:T | rs2906657 | 2.08E-36 | 15 | rs2906657; rs113027130; rs2734897; rs35061193; rs35609229; rs1981550; rs2527884; rs10274982; rs11771331; rs62482172; rs2950520; rs858502; rs34857299; rs35305377; rs2906644 | PILRA |
| **208** | 7 | 100280896 | 129 | 7:100280896:A:C | rs221793 | 2.20E-08 | 3 | rs221793; rs34857299; rs2906644 | GIGYF1 |
| **209** | 7 | 100371100 | 129 | 7:100371100:A:C | rs2734880 | 9.16E-11 | 4 | rs2734880; rs2553022; rs2230585; rs2734897 | ZAN |
| **210** | 7 | 100408386 | 129 | 7:100408386:C:T | rs2466165 | 9.10E-14 | 4 | rs2466165; rs113683483; rs73405382; rs221773 | EPHB4 |
| **211** | 7 | 125532656 | 130 | 7:125532656:G:T | rs2158214 | 2.42E-08 | 1 | rs2158214 | AC005276.1 |
| **212** | 7 | 129256285 | 131 | 7:129256285:C:T | rs10270813 | 6.07E-13 | 4 | rs10270813; rs10254366; rs10954250; 7:129226638_CA_C | NRF1 |
| **213** | 7 | 143108158 | 132 | 7:143108158:C:T | rs7810606 | 3.72E-13 | 8 | rs7810606; rs11767557; 7:143111202_CCA_C; rs55697090; rs1131885; 7:143100513_CT_C; rs4726618; rs3935067 | EPHA1-AS1 |
| **214** | 7 | 143132183 | 132 | 7:143132183:A:G | rs9640386 | 4.97E-08 | 5 | rs9640386; 7:143100513_CT_C; rs3935067; 7:143111202_CCA_C; rs55697090 | EPHA1-AS1 |
| **215** | 7 | 146254508 | 133 | 7:146254508:A:G | rs12154459 | 8.41E-09 | 1 | rs12154459 | CNTNAP2 |
| **216** | 8 | 5681883 | 134 | 8:5681883:A:T | rs73189737 | 3.57E-08 | 1 | rs73189737 | RP11-728L1.1 |
| **217** | 8 | 18712920 | 135 | 8:18712920:C:T | rs59239262 | 1.34E-08 | 1 | rs59239262 | PSD3 |
| **218** | 8 | 27205046 | 136 | 8:27205046:A:AG | 8:27205046_AG_A | 6.52E-19 | 4 | 8:27205046_AG_A; rs2741342; rs2565045; rs4534095 | PTK2B |
| **219** | 8 | 27402777 | 136 | 8:27402777:A:G | rs7341557 | 1.07E-10 | 3 | rs7341557; rs34181358; rs2741342 | EPHX2 |
| **220** | 8 | 27413421 | 136 | 8:27413421:G:T | rs2640725 | 1.56E-09 | 2 | rs2640725; rs7844965 | GULOP |
| **221** | 8 | 27485120 | 136 | 8:27485120:C:G | rs525716 | 2.65E-09 | 5 | rs525716; 8:27491006_TA_T; rs4732746; rs34173549; rs569205 | SCARA3 |
| **222** | 8 | 27504228 | 136 | 8:27504228:A:G | rs17383366 | 1.48E-08 | 1 | rs17383366 | SCARA3 |
| **223** | 8 | 27743062 | 136 | 8:27743062:A:C | rs17481534 | 4.39E-09 | 4 | rs17481534; 8:27491006_TA_T; rs4732746; rs34173549 | SCARA5 |
| **224** | 8 | 42667432 | 137 | 8:42667432:C:G | rs62515894 | 6.51E-17 | 5 | rs62515894; rs7839894; rs13269381; rs4737055; rs2005165 | CHRNA6 |
| **225** | 8 | 43687523 | 138 | 8:43687523:A:G | rs75723456 | 2.68E-12 | 2 | rs75723456; rs4976911 | RP11-643N23.1 |
| **226** | 8 | 47332028 | 139 | 8:47332028:C:G | rs13252503 | 8.91E-14 | 1 | rs13252503 | HSPA8P13 |
| **227** | 8 | 49064181 | 140 | 8:49064181:C:T | rs76557543 | 2.48E-09 | 1 | rs76557543 | RP11-769N21.2 |
| **228** | 8 | 49604591 | 141 | 8:49604591:C:T | rs10090044 | 1.44E-10 | 2 | rs10090044; rs75815822 | RP11-770E5.1 |
| **229** | 8 | 51965536 | 142 | 8:51965536:A:ATATT | 8:51965536_ATATT_A | 1.28E-08 | 1 | 8:51965536_ATATT_A | SNORA7 |
| **230** | 8 | 78458874 | 143 | 8:78458874:C:T | rs77342820 | 7.02E-10 | 1 | rs77342820 | RP11-38H17.1 |
| **231** | 8 | 99636229 | 144 | 8:99636229:C:T | rs6992925 | 8.03E-09 | 1 | rs6992925 | STK3 |
| **232** | 8 | 110654538 | 145 | 8:110654538:A:G | rs11986510 | 3.19E-09 | 2 | rs11986510; rs60573964 | SYBU |
| **233** | 8 | 128507995 | 146 | 8:128507995:C:T | rs1374626 | 1.63E-11 | 2 | rs1374626; rs16902169 | CASC8 |
| **234** | 9 | 32128797 | 147 | 9:32128797:G:T | rs76095252 | 2.31E-08 | 2 | rs76095252; rs192871786 | RNA5SP281 |
| **235** | 9 | 35648008 | 148 | 9:35648008:A:G | rs2153240 | 4.13E-10 | 2 | rs2153240; rs56249943 | RP11-331F9.4 |
| **236** | 9 | 36549836 | 149 | 9:36549836:G:GT | rs113133772 | 3.27E-10 | 1 | rs113133772 | MELK |
| **237** | 9 | 93391288 | 150 | 9:93391288:C:T | rs183428791 | 1.79E-16 | 3 | rs183428791; rs73503379; rs547946 | DIRAS2 |
| **238** | 9 | 107031327 | 151 | 9:107031327:C:CT | rs35904446 | 3.16E-09 | 1 | rs35904446 | RP11-86L19.2 |
| **239** | 9 | 111813111 | 152 | 9:111813111:T:TA | rs199816612 | 4.32E-14 | 6 | rs199816612; rs7038888; rs111693948; rs10512388; rs4978759; rs141184426 | TMEM245 |
| **240** | 9 | 130297409 | 153 | 9:130297409:C:T | rs2247361 | 1.55E-08 | 1 | rs2247361 | FAM129B |
| **241** | 10 | 6728410 | 154 | 10:6728410:A:G | rs146828526 | 4.80E-09 | 1 | rs146828526 | RP11-554I8.2 |
| **242** | 10 | 11718713 | 155 | 10:11718713:A:G | rs7912495 | 9.20E-09 | 1 | rs7912495 | RP11-138I18.2 |
| **243** | 10 | 15346448 | 156 | 10:15346448:C:T | rs11812587 | 1.65E-08 | 1 | rs11812587 | FAM171A1 |
| **244** | 10 | 22801839 | 157 | 10:22801839:A:G | rs4304661 | 2.61E-08 | 1 | rs4304661 | PIP4K2A |
| **245** | 10 | 27960532 | 158 | 10:27960532:A:G | rs2815548 | 7.31E-10 | 1 | rs2815548 | MKX |
| **246** | 10 | 33561560 | 159 | 10:33561560:A:G | rs17296436 | 1.83E-08 | 1 | rs17296436 | NRP1 |
| **247** | 10 | 35324619 | 160 | 10:35324619:C:T | rs7079205 | 2.39E-10 | 2 | rs7079205; rs4934541 | CUL2 |
| **248** | 10 | 35870714 | 160 | 10:35870714:C:T | rs12770412 | 6.58E-09 | 2 | rs12770412; rs4934541 | CCNY |
| **249** | 10 | 37140516 | 161 | 10:37140516:T:TTTTATTTA | rs778472728 | 2.09E-15 | 11 | rs778472728; rs7099719; rs2504392; rs2505172; rs2486120; rs1767387; rs2185863; rs1855638; rs12770632; 10:37107624_ATT_A; rs10827731 | RP11-322I2.1 |
| **250** | 10 | 37263182 | 161 | 10:37263182:A:C | rs11010967 | 4.53E-08 | 2 | rs11010967; rs2504392 | ARL6IP1P2 |
| **251** | 10 | 43333894 | 162 | 10:43333894:G:T | rs11597306 | 2.77E-08 | 3 | rs11597306; rs652998; rs1815706 | BMS1 |
| **252** | 10 | 43352894 | 162 | 10:43352894:C:T | rs2795507 | 3.34E-14 | 3 | rs2795507; rs652998; rs2795531 | RP11-124O11.1 |
| **253** | 10 | 43621712 | 162 | 10:43621712:A:G | rs2742239 | 3.66E-08 | 1 | rs2742239 | RET |
| **254** | 10 | 60056114 | 163 | 10:60056114:A:C | rs2590374 | 7.05E-09 | 1 | rs2590374 | CISD1 |
| **255** | 10 | 68490976 | 164 | 10:68490976:C:T | rs10509277 | 2.67E-08 | 1 | rs10509277 | CTNNA3 |
| **256** | 10 | 68608120 | 164 | 10:68608120:C:T | rs7099541 | 4.56E-08 | 1 | rs7099541 | CTNNA3 |
| **257** | 10 | 123781134 | 165 | 10:123781134:A:G | rs2459085 | 1.10E-11 | 1 | rs2459085 | TACC2 |
| **258** | 11 | 25538293 | 166 | 11:25538293:C:T | rs11028743 | 2.48E-08 | 1 | rs11028743 | AC015820.1 |
| **259** | 11 | 46691737 | 167 | 11:46691737:A:G | rs4606447 | 2.91E-08 | 2 | rs4606447; 11:47371842_AT_A | ATG13 |
| **260** | 11 | 47001908 | 167 | 11:47001908:C:G | rs7130812 | 1.15E-08 | 7 | rs7130812; rs11039114; rs3740691; rs67871383; rs3740688; rs9988865; rs72895811 | C11orf49 |
| **261** | 11 | 47197153 | 167 | 11:47197153:C:T | rs75290815 | 8.66E-21 | 11 | rs75290815; rs7944419; rs7939345; rs11607114; rs76400970; rs60602051; rs9988865; rs58385891; rs72895811; rs11039114; rs3740691 | ARFGAP2 |
| **262** | 11 | 47391948 | 167 | 11:47391948:A:G | rs10437655 | 8.54E-37 | 17 | rs10437655; rs11039281; rs7107356; rs151189779; rs2930191; rs7939345; rs77210655; rs11607114; rs187440142; rs11039638; rs563960872; rs72895811; rs11039114; rs7944419; rs67871383; 11:47371842_AT_A; rs3740688 | SPI1 |
| **263** | 11 | 47758599 | 167 | 11:47758599:A:C | rs55743056 | 6.95E-12 | 7 | rs55743056; rs2930191; rs1228001; rs67871383; rs3740688; rs72911714; rs7107356 | FNBP4 |
| **264** | 11 | 47775761 | 167 | 11:47775761:G:GAAA | 11:47775761_GAAA_G | 7.04E-13 | 5 | 11:47775761_GAAA_G; rs7939345; rs11607114; rs11039638; rs11530245 | FNBP4 |
| **265** | 11 | 47800904 | 167 | 11:47800904:C:T | rs60137857 | 2.54E-14 | 6 | rs60137857; rs117770498; rs1228001; 11:47371842_AT_A; rs3740688; rs7107356 | NUP160 |
| **266** | 11 | 48346996 | 167 | 11:48346996:A:G | rs12794960 | 3.93E-30 | 6 | rs12794960; rs144974052; rs72912680; rs6485964; rs35634647; rs117770498 | OR4C3 |
| **267** | 11 | 48472051 | 167 | 11:48472051:A:G | rs61915439 | 7.62E-18 | 5 | rs61915439; rs61915456; rs10839231; rs11039281; rs151189779 | OR4C9P |
| **268** | 11 | 48709133 | 167 | 11:48709133:C:T | rs75184591 | 2.03E-40 | 23 | rs75184591; rs7951780; rs11040151; rs10839197; rs368284713; rs2696916; rs12275064; rs172129; rs35634647; rs56361714; rs12283059; rs55749259; rs2930191; rs7939345; rs1228001; rs77210655; rs11607114; rs187440142; rs11039638; rs10769342; rs563960872; rs12786632; rs11530245 | OR4A44P |
| **269** | 11 | 48983862 | 167 | 11:48983862:A:G | rs12575218 | 4.24E-08 | 13 | rs12575218; rs368284713; rs2696916; rs12275064; rs12283059; rs77210655; rs11607114; rs187440142; rs11039638; rs563960872; rs12786632; rs7951780; rs10839197 | RP11-56P9.8 |
| **270** | 11 | 50189874 | 167 | 11:50189874:C:CAA | 11:50189874_CAA_C | 8.50E-33 | 11 | 11:50189874_CAA_C; rs12788022; rs72907342; rs12800768; rs7395833; rs12365294; rs36177431; rs35634647; rs71479308; rs10839285; rs7949044 | RP11-347H15.6 |
| **271** | 11 | 50468801 | 167 | 11:50468801:C:T | rs1813937 | 6.15E-42 | 13 | rs1813937; rs12365294; rs11245786; rs4463839; rs10839285; rs10769591; rs12283059; rs374975971; rs151086397; rs4468343; rs139564703; rs11533319; rs1608244 | RP11-574M7.2 |
| **272** | 11 | 51253295 | 167 | 11:51253295:G:T | rs4312050 | 7.15E-22 | 6 | rs4312050; rs4881862; rs12800768; rs7395833; rs12365294; rs36177431 | AC110283.1 |
| **273** | 11 | 51476467 | 167 | 11:51476467:A:C | rs4515954 | 2.35E-26 | 3 | rs4515954; rs199614839; rs4463839 | OR4C7P |
| **274** | 11 | 54892370 | 168 | 11:54892370:C:T | rs58904316 | 2.44E-26 | 10 | rs58904316; rs35391355; 11:55267156_AT_A; rs10896993; rs2013618; 11:55762826_AT_A; rs117804393; rs540746002; rs558514573; rs186643527 | TRIM48 |
| **275** | 11 | 54960592 | 168 | 11:54960592:A:G | rs12807494 | 8.71E-14 | 7 | rs12807494; rs10896993; 11:55585267_CA_C; rs4939007; rs2335130; rs9666492; rs35391355 | TRIM48 |
| **276** | 11 | 55501163 | 168 | 11:55501163:G:T | rs191675955 | 1.42E-11 | 3 | rs191675955; 11:55585267_CA_C; rs35778494 | OR5D3P |
| **277** | 11 | 55526769 | 168 | 11:55526769:C:T | rs297069 | 3.96E-08 | 4 | rs297069; rs4939007; rs9666492; rs35391355 | OR5D17P |
| **278** | 11 | 55567256 | 168 | 11:55567256:C:T | rs72918199 | 2.45E-23 | 5 | rs72918199; rs35778494; rs11228739; rs511910; rs9666492 | OR5D14 |
| **279** | 11 | 56058535 | 168 | 11:56058535:C:T | rs11600896 | 2.89E-14 | 9 | rs11600896; rs76710975; rs77609032; rs2013618; rs4939007; 11:55762826_AT_A; rs2449126; rs117804393; rs773344516 | OR8H1 |
| **280** | 11 | 56549480 | 168 | 11:56549480:A:G | rs72923892 | 8.23E-10 | 5 | rs72923892; rs511910; 11:55585267_CA_C; rs12797149; rs11228739 | OR5G1P |
| **281** | 11 | 57010037 | 169 | 11:57010037:C:T | rs78277055 | 3.34E-11 | 1 | rs78277055 | APLNR |
| **282** | 11 | 57089203 | 169 | 11:57089203:G:GGCA | rs748161345 | 4.23E-08 | 1 | rs748161345 | TNKS1BP1 |
| **283** | 11 | 57092768 | 169 | 11:57092768:A:G | rs72921997 | 2.82E-14 | 1 | rs72921997 | RP11-872D17.4 |
| **284** | 11 | 57156438 | 169 | 11:57156438:A:G | rs3741089 | 3.30E-11 | 1 | rs3741089 | PRG2:RP11-872D17.8 |
| **285** | 11 | 58340484 | 170 | 11:58340484:C:T | rs7110210 | 5.32E-10 | 1 | rs7110210 | LPXN |
| **286** | 11 | 59968705 | 171 | 11:59968705:A:G | rs367670643 | 2.36E-21 | 10 | rs367670643; rs2868099; rs2044982; rs4522186; rs749908546; rs1286289; 11:59832687_CTT_C; rs580064; rs35421721; rs474123 | MS4A4E |
| **287** | 11 | 73982449 | 172 | 11:73982449:C:T | rs4121668 | 8.47E-10 | 1 | rs4121668 | P4HA3 |
| **288** | 11 | 84832089 | 173 | 11:84832089:A:C | rs117627917 | 8.77E-09 | 1 | rs117627917 | DLG2 |
| **289** | 11 | 85190334 | 173 | 11:85190334:A:C | rs188255 | 5.58E-09 | 1 | rs188255 | DLG2 |
| **290** | 11 | 85588544 | 173 | 11:85588544:A:T | rs68059633 | 2.09E-11 | 10 | rs68059633; rs137950229; rs11608136; rs11825598; rs598561; rs7101740; rs7938634; rs640620; rs67821335; rs10898417 | CCDC83 |
| **291** | 11 | 85716032 | 173 | 11:85716032:C:T | rs680119 | 6.84E-10 | 10 | rs680119; rs604767; rs11234542; 11:85837459_GT_G; rs67598967; rs5793182; rs7113976; rs7129687; rs60185300; rs659801 | PICALM |
| **292** | 11 | 85863080 | 173 | 11:85863080:A:C | rs56157503 | 2.08E-10 | 10 | rs56157503; rs7113976; rs7937794; rs10454495; rs137950229; rs60185300; 11:85759879_CAAA_C; 11:85837459_GT_G; rs5793182; rs11234552 | RNU6-560P |
| **293** | 11 | 85867875 | 173 | 11:85867875:A:G | rs10792832 | 6.03E-31 | 18 | rs10792832; rs7113976; rs7129687; rs11234568; rs7937794; rs3888884; rs60185300; rs11825598; rs598561; rs659801; rs7101740; 11:85759879_CAAA_C; rs604767; 11:85837459_GT_G; rs67598967; rs5793182; rs7938634; rs11234552 | RNU6-560P |
| **294** | 11 | 86401281 | 174 | 11:86401281:A:G | rs34227026 | 2.79E-10 | 2 | rs34227026; rs2512983 | CTD-2005H7.1 |
| **295** | 11 | 125461039 | 175 | 11:125461039:T:TA | rs34354348 | 3.06E-08 | 1 | rs34354348 | STT3A-AS1 |
| **296** | 12 | 32010784 | 176 | 12:32010784:C:T | rs10771877 | 1.94E-09 | 1 | rs10771877 | RP11-428G5.4 |
| **297** | 12 | 32146906 | 176 | 12:32146906:A:T | rs7307638 | 4.82E-09 | 2 | rs7307638; 12:32166247_TACA_T | KIAA1551 |
| **298** | 12 | 34454301 | 177 | 12:34454301:A:G | rs7314457 | 3.47E-24 | 16 | rs7314457; rs11053229; rs7966130; rs12424735; rs9706386; rs10844589; rs369941489; rs7313428; 12:33780794_C_CT; 12:33805757_CGGTGTGTG_C; rs1979580; rs2171534; rs114895731; rs12310956; rs10772152; rs4643153 | RP13-7D7.1 |
| **299** | 12 | 38666013 | 178 | 12:38666013:A:T | rs10880819 | 5.48E-25 | 13 | rs10880819; rs12822662; rs7957376; 12:39123758_ATCT_A; rs11609849; rs56149694; rs7970834; rs61922013; rs7302705; rs11495511; rs34366092; rs202048324; rs12299971 | Y_RNA |
| **300** | 12 | 39425199 | 178 | 12:39425199:A:G | rs1449840 | 1.23E-13 | 7 | rs1449840; rs7970834; rs34894002; rs17126850; rs4255576; rs116896515; rs564727247 | RP11-554L12.2 |
| **301** | 12 | 39900742 | 178 | 12:39900742:A:AT | rs200698156 | 2.79E-08 | 2 | rs200698156; rs10877426 | RP11-242C24.3 |
| **302** | 12 | 39988971 | 178 | 12:39988971:A:G | rs11172721 | 4.08E-08 | 7 | rs11172721; rs11172897; rs10877426; rs113895504; 12:39123758_ATCT_A; rs11609849; rs7487535 | ABCD2 |
| **303** | 12 | 40105272 | 178 | 12:40105272:C:T | rs142352564 | 1.60E-11 | 8 | rs142352564; rs10877426; rs61931461; rs113895504; rs17126850; rs4255576; rs11172687; rs11172897 | C12orf40 |
| **304** | 12 | 40238338 | 178 | 12:40238338:A:T | rs4542485 | 4.34E-15 | 5 | rs4542485; rs79987201; rs370765986; rs7311756; rs7487535 | C12orf40:SLC2A13 |
| **305** | 12 | 40367171 | 178 | 12:40367171:C:T | rs12810368 | 1.93E-09 | 3 | rs12810368; rs7311756; rs370765986 | SLC2A13 |
| **306** | 12 | 40730463 | 178 | 12:40730463:C:T | rs2404835 | 6.95E-09 | 3 | rs2404835; rs11175943; rs11175958 | LRRK2 |
| **307** | 12 | 40784253 | 178 | 12:40784253:A:G | rs11613829 | 1.24E-15 | 9 | rs11613829; rs10878714; rs12422396; rs61931461; rs2200086; rs113895504; rs7969677; rs11175943; rs11175958 | MUC19 |
| **308** | 12 | 50803591 | 179 | 12:50803591:A:T | rs7301142 | 1.02E-08 | 1 | rs7301142 | LARP4 |
| **309** | 12 | 61233977 | 180 | 12:61233977:A:C | rs61923796 | 4.78E-09 | 1 | rs61923796 | RP11-471N19.1 |
| **310** | 12 | 72218419 | 181 | 12:72218419:A:G | rs141997660 | 1.45E-08 | 1 | rs141997660 | RP11-2H8.3 |
| **311** | 12 | 84368364 | 182 | 12:84368364:A:G | rs6539809 | 3.90E-10 | 1 | rs6539809 | SNORA3 |
| **312** | 12 | 87905754 | 183 | 12:87905754:A:C | rs79267673 | 1.04E-10 | 1 | rs79267673 | RP11-248E9.1 |
| **313** | 12 | 95195293 | 184 | 12:95195293:G:T | rs6538539 | 1.83E-08 | 1 | rs6538539 | KRT19P2 |
| **314** | 12 | 110051149 | 185 | 12:110051149:A:G | rs73200429 | 3.52E-09 | 2 | rs73200429; rs61942626 | MVK |
| **315** | 12 | 110232684 | 185 | 12:110232684:A:G | rs11068311 | 8.67E-09 | 1 | rs11068311 | TRPV4 |
| **316** | 12 | 111269159 | 185 | 12:111269159:A:G | rs61942658 | 2.34E-08 | 2 | rs61942658; rs61942626 | RP1-74B13.2 |
| **317** | 12 | 112341613 | 186 | 12:112341613:C:T | rs73205628 | 7.50E-13 | 4 | rs73205628; rs73207627; rs117769479; rs73193171 | ADAM1A |
| **318** | 12 | 113167134 | 186 | 12:113167134:A:T | rs7313341 | 3.17E-10 | 3 | rs7313341; rs4766653; rs494273 | RPH3A |
| **319** | 12 | 113216693 | 186 | 12:113216693:A:G | rs4238032 | 9.05E-11 | 2 | rs4238032; rs4766653 | RPH3A |
| **320** | 12 | 122028904 | 187 | 12:122028904:A:C | rs28507431 | 8.09E-17 | 6 | rs28507431; rs113526867; rs117745126; 12:122172800_CAA_C; 12:122176889_TC_T; rs61952259 | RP13-941N14.1 |
| **321** | 12 | 122950794 | 188 | 12:122950794:C:T | rs61956096 | 9.16E-11 | 1 | rs61956096 | ZCCHC8 |
| **322** | 12 | 123329690 | 189 | 12:123329690:C:T | rs897390 | 3.53E-09 | 1 | rs897390 | HIP1R |
| **323** | 12 | 123339358 | 189 | 12:123339358:C:G | rs2292137 | 2.07E-10 | 1 | rs2292137 | HIP1R |
| **324** | 12 | 123768925 | 189 | 12:123768925:G:GTTTGT | rs796884303 | 5.48E-11 | 4 | rs796884303; rs61955214; rs1716183; rs1716162 | RNA5SP375 |
| **325** | 13 | 52397614 | 190 | 13:52397614:A:T | rs10220242 | 5.48E-09 | 1 | rs10220242 | RP11-327P2.5 |
| **326** | 13 | 52496060 | 190 | 13:52496060:A:G | rs7988558 | 8.99E-19 | 4 | rs7988558; rs61957450; rs61957455; rs9536253 | ATP7B |
| **327** | 13 | 52862570 | 190 | 13:52862570:G:T | rs9535966 | 1.11E-36 | 10 | rs9535966; rs9596645; rs2476356; rs35799542; rs386771059; rs34921261; rs9536253; rs113789076; rs61957450; rs61957455 | RP11-248G5.8:TPTE2P2 |
| **328** | 13 | 52883003 | 190 | 13:52883003:A:G | rs114747892 | 6.65E-32 | 1 | rs114747892 | TPTE2P2 |
| **329** | 13 | 53234583 | 190 | 13:53234583:A:G | rs9568747 | 9.12E-11 | 3 | rs9568747; rs7139630; rs9535784 | SUGT1 |
| **330** | 13 | 65258629 | 191 | 13:65258629:C:T | rs9571250 | 2.16E-08 | 1 | rs9571250 | LGMNP1 |
| **331** | 14 | 44934571 | 192 | 14:44934571:A:T | rs382066 | 5.85E-12 | 3 | rs382066; rs12884395; rs4906531 | RP11-99L13.1 |
| **332** | 14 | 46613186 | 193 | 14:46613186:C:T | rs80268960 | 7.27E-10 | 3 | rs80268960; rs28743162; rs76584360 | LINC00871 |
| **333** | 14 | 49594152 | 194 | 14:49594152:A:C | rs11625334 | 1.44E-08 | 1 | rs11625334 | RP11-816J8.1 |
| **334** | 14 | 50811025 | 195 | 14:50811025:C:T | rs1465160 | 4.56E-12 | 1 | rs1465160 | CDKL1 |
| **335** | 14 | 53240059 | 196 | 14:53240059:A:AT | rs373046986 | 4.53E-11 | 1 | rs373046986 | STYX |
| **336** | 14 | 73411524 | 197 | 14:73411524:C:T | rs2806045 | 2.64E-08 | 1 | rs2806045 | DCAF4 |
| **337** | 14 | 86262897 | 198 | 14:86262897:A:C | rs12434216 | 1.49E-08 | 2 | rs12434216; rs17121444 | CTD-2341M24.1 |
| **338** | 14 | 92845636 | 199 | 14:92845636:G:T | rs35627364 | 1.59E-10 | 2 | rs35627364; rs11626106 | SLC24A4 |
| **339** | 14 | 92938855 | 199 | 14:92938855:A:G | rs12590654 | 1.29E-08 | 1 | rs12590654 | SLC24A4 |
| **340** | 15 | 24649154 | 200 | 15:24649154:C:CAT | rs33960156 | 1.01E-14 | 8 | rs33960156; rs7497589; rs5006859; rs11852485; rs2201880; rs12905662; rs35881256; rs56137049 | PWRN3 |
| **341** | 15 | 43509980 | 201 | 15:43509980:C:T | rs118011588 | 1.52E-09 | 1 | rs118011588 | EPB42 |
| **342** | 15 | 44188854 | 201 | 15:44188854:C:T | rs533143 | 4.78E-08 | 1 | rs533143 | FRMD5 |
| **343** | 15 | 44900675 | 202 | 15:44900675:C:T | rs36014111 | 8.65E-09 | 1 | rs36014111 | SPG11 |
| **344** | 15 | 51057868 | 203 | 15:51057868:A:C | rs3896609 | 3.49E-11 | 3 | rs3896609; rs7183420; rs1124769 | SPPL2A |
| **345** | 15 | 59057023 | 204 | 15:59057023:A:T | rs602602 | 1.04E-13 | 7 | rs602602; rs28537903; rs632811; 15:59207390_AAAAAACAAAAAC_A; rs3985721; rs4775086; rs796338552 | RP11-30K9.6 |
| **346** | 15 | 59282073 | 204 | 15:59282073:C:CT | 15:59282073_CT_C | 8.60E-09 | 5 | 15:59282073_CT_C; rs28537903; rs632811; 15:59207390_AAAAAACAAAAAC_A; rs3985721 | RNF111 |
| **347** | 15 | 63641427 | 205 | 15:63641427:C:T | rs11634841 | 5.07E-10 | 8 | rs11634841; rs75530524; rs11071739; rs28386949; rs16946801; rs2018400; rs1039289; rs35763222 | CA12 |
| **348** | 15 | 63760569 | 205 | 15:63760569:C:T | rs4984289 | 1.79E-16 | 3 | rs4984289; rs11071755; rs7174110 | AC007950.1 |
| **349** | 15 | 64170637 | 205 | 15:64170637:C:T | rs150357968 | 3.36E-09 | 2 | rs150357968; 15:64109339_TAA_T | MIR422A |
| **350** | 15 | 64406024 | 205 | 15:64406024:T:TAAAG | 15:64406024_TAAAG_T | 4.75E-08 | 1 | 15:64406024_TAAAG_T | SNX1 |
| **351** | 15 | 74560363 | 206 | 15:74560363:C:G | rs7163055 | 1.10E-08 | 1 | rs7163055 | CCDC33 |
| **352** | 15 | 75220968 | 207 | 15:75220968:C:T | rs11072513 | 6.19E-10 | 3 | rs11072513; rs773107888; rs548686121 | COX5A |
| **353** | 15 | 76772062 | 208 | 15:76772062:C:T | rs2469249 | 5.26E-23 | 14 | rs2469249; rs11072599; rs2896954; rs2017103; 15:77185301_GCACATATA_G; rs2458252; rs62027291; rs35018118; rs8029854; rs3936040; rs35134156; rs2456045; rs11072577; rs4886795 | SCAPER |
| **354** | 15 | 77572906 | 208 | 15:77572906:C:CT | 15:77572906_CT_C | 3.36E-11 | 3 | 15:77572906_CT_C; rs62027291; rs3936040 | PEAK1 |
| **355** | 15 | 77888252 | 208 | 15:77888252:C:G | rs12902811 | 1.83E-14 | 4 | rs12902811; rs2017103; rs8029854; rs12912181 | RP11-307C19.2 |
| **356** | 15 | 80595221 | 209 | 15:80595221:G:T | rs961751 | 1.65E-08 | 1 | rs961751 | LINC00927 |
| **357** | 15 | 83320547 | 210 | 15:83320547:A:G | rs72751690 | 2.26E-08 | 1 | rs72751690 | RP11-752G15.3 |
| **358** | 15 | 85611817 | 211 | 15:85611817:T:TA | rs34743976 | 2.69E-08 | 1 | rs34743976 | PDE8A |
| **359** | 16 | 16894858 | 212 | 16:16894858:A:G | rs6498627 | 3.68E-08 | 1 | rs6498627 | RP11-14N9.2 |
| **360** | 16 | 30018720 | 213 | 16:30018720:C:T | rs12921753 | 2.51E-12 | 2 | rs12921753; rs9783783 | DOC2A |
| **361** | 16 | 30636777 | 214 | 16:30636777:C:T | rs7499339 | 5.76E-14 | 10 | rs7499339; rs150458589; rs28853644; rs4889490; rs140820592; rs2884737; rs9972727; rs1549299; rs6565192; rs74249949 | RP11-146F11.3 |
| **362** | 16 | 31318290 | 214 | 16:31318290:C:CAT | 16:31318290_CAT_C | 2.68E-11 | 2 | 16:31318290_CAT_C; rs2884737 | ITGAM |
| **363** | 16 | 31368178 | 214 | 16:31368178:C:T | rs7190997 | 1.74E-12 | 6 | rs7190997; rs7203472; rs140820592; rs2884737; rs9972727; rs17708638 | ITGAX |
| **364** | 16 | 46976245 | 215 | 16:46976245:T:TA | rs35331432 | 4.77E-08 | 1 | rs35331432 | GPT2 |
| **365** | 16 | 47816476 | 215 | 16:47816476:C:CT | rs199732214 | 1.28E-15 | 7 | rs199732214; 16:47893949_GAT_G; 16:47972068_TA_T; 16:47980985_CA_C; rs16945387; rs16945493; 16:47743517_CA_C | RP11-523L20.2 |
| **366** | 16 | 67667163 | 216 | 16:67667163:G:GGT | 16:67667163_GGT_G | 3.87E-08 | 2 | 16:67667163_GGT_G; rs532742216 | CTCF |
| **367** | 16 | 69669655 | 217 | 16:69669655:C:G | rs62049970 | 3.02E-11 | 1 | rs62049970 | NFAT5 |
| **368** | 16 | 70048383 | 217 | 16:70048383:A:C | rs1862664 | 3.20E-10 | 4 | rs1862664; rs112394000; rs6499327; rs9929531 | PDXDC2P:PDXDC2P |
| **369** | 16 | 70575214 | 217 | 16:70575214:A:G | rs4985532 | 7.03E-09 | 3 | rs4985532; rs71401820; rs4985556 | SF3B3 |
| **370** | 16 | 70676478 | 217 | 16:70676478:C:T | rs12598456 | 2.52E-21 | 8 | rs12598456; rs71401820; rs55704776; rs4985556; rs16964335; rs72792820; rs6499327; rs9929531 | IL34 |
| **371** | 16 | 70720630 | 217 | 16:70720630:C:G | rs35874206 | 3.35E-14 | 5 | rs35874206; rs4985562; rs7203578; rs9929531; rs2124832 | MTSS1L |
| **372** | 16 | 70728477 | 217 | 16:70728477:A:G | rs3785425 | 4.63E-19 | 11 | rs3785425; rs12149236; rs143836946; rs7203578; rs12926985; rs2502671; rs142784127; rs71401820; rs4985556; rs11538963; rs2124832 | VAC14 |
| **373** | 16 | 70729954 | 217 | 16:70729954:C:T | rs62047964 | 1.31E-13 | 4 | rs62047964; rs1875941; rs2161711; rs62055045 | VAC14 |
| **374** | 16 | 71105060 | 217 | 16:71105060:A:G | rs1512607 | 8.82E-09 | 2 | rs1512607; rs4985562 | HYDIN |
| **375** | 16 | 72595796 | 218 | 16:72595796:C:T | rs117276692 | 2.13E-10 | 6 | rs117276692; rs9923575; rs61452478; rs16970692; rs726887; rs117863134 | AC004158.2 |
| **376** | 16 | 78510270 | 219 | 16:78510270:A:G | rs111610950 | 1.38E-08 | 1 | rs111610950 | WWOX |
| **377** | 16 | 79341493 | 220 | 16:79341493:G:T | rs9925135 | 3.24E-12 | 7 | rs9925135; rs11863601; rs1876764; rs1553720; rs7188266; rs8050996; rs13339057 | RNA5SP431 |
| **378** | 16 | 88995687 | 221 | 16:88995687:C:CA | 16:88995687_CA_C | 4.39E-08 | 1 | 16:88995687_CA_C | CBFA2T3 |
| **379** | 16 | 90109711 | 222 | 16:90109711:A:G | rs3743825 | 1.40E-08 | 1 | rs3743825 | GAS8:URAHP |
| **380** | 17 | 3914513 | 223 | 17:3914513:G:T | rs57625058 | 1.88E-08 | 1 | rs57625058 | ZZEF1 |
| **381** | 17 | 4988417 | 224 | 17:4988417:C:T | rs9898019 | 1.41E-10 | 3 | rs9898019; rs562450336; rs17707385 | ZFP3 |
| **382** | 17 | 5105303 | 224 | 17:5105303:A:C | rs78538460 | 2.48E-12 | 1 | rs78538460 | RP11-333E1.1 |
| **383** | 17 | 5147239 | 224 | 17:5147239:A:G | rs8073171 | 4.34E-08 | 3 | rs8073171; rs17707385; rs562450336 | RP11-333E1.1 |
| **384** | 17 | 17427004 | 225 | 17:17427004:A:T | rs73294357 | 4.25E-11 | 5 | rs73294357; rs60343188; rs117924544; rs76528177; rs12949004 | PEMT |
| **385** | 17 | 17878524 | 225 | 17:17878524:C:CTT | 17:17878524_CTT_C | 4.58E-09 | 4 | 17:17878524_CTT_C; rs76528177; rs854786; rs117924544 | LRRC48 |
| **386** | 17 | 19854918 | 226 | 17:19854918:C:G | rs175921 | 1.06E-12 | 2 | rs175921; rs145571077 | AKAP10 |
| **387** | 17 | 21727308 | 227 | 17:21727308:C:G | rs7226114 | 4.99E-08 | 1 | rs7226114 | UBBP4 |
| **388** | 17 | 42108974 | 228 | 17:42108974:A:C | rs375677 | 1.55E-08 | 1 | rs375677 | LSM12 |
| **389** | 17 | 43798360 | 229 | 17:43798360:A:G | rs55938136 | 1.15E-14 | 1 | rs55938136 | CRHR1:RP11-105N13.4 |
| **390** | 17 | 44257788 | 229 | 17:44257788:C:T | rs2696697 | 5.08E-16 | 15 | rs2696697; rs2696531; rs2696531; rs199470; rs199500; rs62065454; rs71279459; rs9303521; rs8067527; rs34186148; rs242937; rs9915721; rs754593; rs8067056; rs35173354 | KANSL1 |
| **391** | 17 | 47316259 | 230 | 17:47316259:A:G | rs850522 | 6.57E-10 | 1 | rs850522 | PHOSPHO1 |
| **392** | 17 | 61560763 | 231 | 17:61560763:C:T | rs4311 | 2.72E-10 | 2 | rs4311; rs4309 | ACE |
| **393** | 18 | 36663160 | 232 | 18:36663160:A:G | rs72886476 | 4.53E-10 | 2 | rs72886476; rs117678874 | RNU6-706P |
| **394** | 19 | 1043638 | 233 | 19:1043638:C:T | rs3752231 | 1.84E-12 | 7 | rs3752231; rs12151021; 19:1051137_CTG_C; 19:1014537_ACGTGATGGGG_A; 19:1032778_TTC_T; rs111278892; rs3795065 | ABCA7 |
| **395** | 19 | 5061345 | 234 | 19:5061345:A:T | rs2009654 | 5.35E-09 | 1 | rs2009654 | KDM4B |
| **396** | 19 | 11238239 | 235 | 19:11238239:C:G | rs2569540 | 1.18E-09 | 1 | rs2569540 | LDLR |
| **397** | 19 | 18512817 | 236 | 19:18512817:T:TAGAAAAAAA | rs754032589 | 1.39E-08 | 1 | rs754032589 | LRRC25 |
| **398** | 19 | 37693764 | 237 | 19:37693764:C:T | rs12462716 | 2.45E-11 | 2 | rs12462716; rs34952147 | CTC-454I21.3:ZNF585B |
| **399** | 19 | 40184489 | 238 | 19:40184489:C:CG | 19:40184489_CG_C | 3.32E-08 | 1 | 19:40184489_CG_C | AC093063.3 |
| **400** | 19 | 44202701 | 239 | 19:44202701:A:G | rs147698232 | 2.86E-12 | 4 | rs147698232; rs36120065; rs140388093; rs77594976 | IRGC |
| **401** | 19 | 44204469 | 239 | 19:44204469:C:G | rs346054 | 3.60E-08 | 2 | rs346054; rs36120065 | IRGC |
| **402** | 19 | 44293278 | 239 | 19:44293278:C:T | rs8104447 | 1.14E-09 | 1 | rs8104447 | AC115522.3 |
| **403** | 19 | 44434937 | 239 | 19:44434937:A:C | rs7255151 | 2.85E-08 | 1 | rs7255151 | ZNF45 |
| **404** | 19 | 44522357 | 239 | 19:44522357:C:T | rs73035978 | 3.70E-21 | 7 | rs73035978; rs73037644; rs72480795; rs117515605; rs77594976; rs47775; rs58591332 | ZNF230 |
| **405** | 19 | 44549793 | 239 | 19:44549793:C:T | rs55715017 | 1.33E-10 | 2 | rs55715017; rs78187790 | ZNF223 |
| **406** | 19 | 44883547 | 239 | 19:44883547:C:T | rs1836278 | 4.42E-15 | 5 | rs1836278; rs8109894; rs11881426; rs2722677; rs62115272 | CTC-512J12.6 |
| **407** | 19 | 44903050 | 239 | 19:44903050:A:T | rs145494710 | 1.14E-11 | 2 | rs145494710; rs204556 | CTC-512J12.6:ZNF285:CTC-512J12.4 |
| **408** | 19 | 44962550 | 239 | 19:44962550:A:C | rs63749402 | 2.84E-08 | 6 | rs63749402; rs55644952; rs544171727; rs35899189; rs8109894; rs204540 | ZNF285B |
| **409** | 19 | 45004823 | 239 | 19:45004823:A:G | rs2293166 | 1.03E-14 | 10 | rs2293166; rs55644952; rs544171727; rs62116891; rs204556; rs204547; rs204540; rs55771561; 19:44997793_GCTCTTGCTCT_G; rs892596 | ZNF180 |
| **410** | 19 | 45080065 | 239 | 19:45080065:C:G | rs846876 | 2.42E-23 | 6 | rs846876; rs1661174; rs1727760; rs74172478; rs846848; rs846881 | CEACAM22P |
| **411** | 19 | 45125197 | 239 | 19:45125197:C:T | rs62119263 | 5.21E-13 | 8 | rs62119263; rs11083743; rs11670070; rs2272021; rs2965156; rs144526132; rs2965160; rs12985991 | IGSF23 |
| **412** | 19 | 45149613 | 239 | 19:45149613:C:T | rs56261258 | 5.11E-66 | 11 | rs56261258; rs2965156; rs35488312; rs58500115; rs79638902; rs55923289; rs4468734; rs386809682; rs59175670; rs11083743; rs11670070 | CTB-171A8.1:PVR |
| **413** | 19 | 45198936 | 239 | 19:45198936:C:T | rs2965162 | 1.49E-27 | 10 | rs2965162; rs117947551; rs2965164; rs80307900; rs203709; rs2965156; rs144526132; rs73568026; rs35488312; rs62120565 | CTB-171A8.1 |
| **414** | 19 | 45200634 | 239 | 19:45200634:C:T | rs80000866 | 2.34E-10 | 1 | rs80000866 | CTB-171A8.1 |
| **415** | 19 | 45205592 | 239 | 19:45205592:C:T | rs8105132 | 4.35E-14 | 11 | rs8105132; rs58500115; rs2965109; rs10422350; rs80307900; rs4803749; rs144526132; rs35488312; rs117947551; rs2965164; rs2919847 | CTB-171A8.1:CEACAM16 |
| **416** | 19 | 45208026 | 239 | 19:45208026:A:C | rs2860314 | 2.59E-12 | 4 | rs2860314; rs58530605; rs62119318; rs2919847 | CTB-171A8.1:CEACAM16 |
| **417** | 19 | 45220896 | 239 | 19:45220896:C:T | rs11881756 | 1.22E-35 | 7 | rs11881756; rs62117161; rs8108110; rs762016934; rs62120565; rs58530605; rs58500115 | CTB-171A8.1 |
| **418** | 19 | 45235700 | 239 | 19:45235700:C:T | rs74607435 | 2.23E-19 | 1 | rs74607435 | snoZ6 |
| **419** | 19 | 45245698 | 239 | 19:45245698:C:T | rs7249244 | 1.22E-14 | 6 | rs7249244; rs4803749; rs762016934; rs10422350; rs779632968; rs2927437 | BCL3 |
| **420** | 19 | 45253542 | 239 | 19:45253542:C:CTTTG | rs10629382 | 3.34E-117 | 10 | rs10629382; rs762016934; rs2965164; rs2919847; rs2965109; rs10422350; rs779632968; rs55923289; rs2927437; rs4803749 | BCL3 |
| **421** | 19 | 45298069 | 239 | 19:45298069:A:G | rs10419669 | 5.01E-12 | 3 | rs10419669; rs139576276; rs1081106 | CBLC |
| **422** | 19 | 45302951 | 239 | 19:45302951:A:G | rs2967668 | 7.63E-56 | 6 | rs2967668; 19:45316330_TGGA_T; rs112949028; rs2927455; rs2927447; rs139576276 | CBLC |
| **423** | 19 | 45324138 | 239 | 19:45324138:A:G | rs28399637 | 6.80E-192 | 6 | rs28399637; rs7026; rs149214960; rs12974942; rs57537848; rs4803764 | BCAM |
| **424** | 19 | 45335676 | 239 | 19:45335676:A:G | rs56394238 | 2.78E-91 | 14 | rs56394238; rs10415074; rs55840414; rs12974942; rs57537848; rs2972566; rs2972558; rs4803764; rs34165484; rs393584; rs7343130; rs149214960; rs4803760; rs2972555 | BCAM |
| **425** | 19 | 45345787 | 239 | 19:45345787:A:T | rs111371860 | 1.25E-38 | 4 | rs111371860; rs1001611; rs7343130; rs2972555 | PVRL2 |
| **426** | 19 | 45349177 | 239 | 19:45349177:C:G | rs77241309 | 9.24E-14 | 11 | rs77241309; rs2972558; rs34165484; 19:45376044_GTATTTAT_G; rs393584; rs405697; rs7026; rs78986976; rs7343130; rs2972555; rs1001611 | PVRL2 |
| **427** | 19 | 45382717 | 239 | 19:45382717:A:G | rs406456 | 1.16E-103 | 12 | rs406456; rs3852861; rs1081106; rs2972562; rs2972558; 19:45367430_AAAAC_A; rs34165484; rs395908; 19:45376044_GTATTTAT_G; rs393584; rs73050293; rs6859 | PVRL2 |
| **428** | 19 | 45383037 | 239 | 19:45383037:A:T | rs11673139 | 1.76E-24 | 7 | rs11673139; rs157580; rs11668327; rs484195; rs2972555; rs768441224; 19:45376044_GTATTTAT_G | PVRL2 |
| **429** | 19 | 45386467 | 239 | 19:45386467:G:GTAA | rs142042446 | 0 | 7 | rs142042446; rs157582; rs10119; rs483082; rs814573; rs41289512; rs6859 | PVRL2:CTB-129P6.4 |
| **430** | 19 | 45403924 | 239 | 19:45403924:C:T | rs1160984 | 3.36E-25 | 4 | rs1160984; rs484195; rs395908; rs393584 | TOMM40 |
| **431** | 19 | 45407788 | 239 | 19:45407788:A:G | rs7259620 | 2.60E-156 | 18 | rs7259620; rs1081106; rs483082; rs59325138; rs484195; rs7026; rs7343130; rs2972562; rs1001611; rs12974942; rs34165484; 19:45376044_GTATTTAT_G; rs393584; rs73050293; rs157580; rs11668327; rs405697; rs10119 | TOMM40 |
| **432** | 19 | 45413233 | 239 | 19:45413233:G:T | rs1065853 | 1.25E-108 | 4 | rs1065853; rs483082; rs28795074; rs768441224 | APOE |
| **433** | 19 | 45477111 | 239 | 19:45477111:C:T | rs204480 | 7.79E-19 | 4 | rs204480; 19:45502368_TAAA_T; rs2376868; rs35670684 | CLPTM1 |
| **434** | 19 | 45477231 | 239 | 19:45477231:C:G | rs57465754 | 4.32E-64 | 7 | rs57465754; rs35194062; rs2376868; rs35194383; rs145887105; rs5167; rs35670684 | CLPTM1 |
| **435** | 19 | 45530351 | 239 | 19:45530351:C:T | rs34583389 | 1.50E-36 | 6 | rs34583389; rs1076986; rs62117227; rs1968449; rs2376868; rs35194383 | RELB |
| **436** | 19 | 45574113 | 239 | 19:45574113:G:T | rs201269048 | 2.07E-14 | 1 | rs201269048 | CLASRP |
| **437** | 19 | 45582402 | 239 | 19:45582402:C:G | rs7251911 | 7.66E-29 | 1 | rs7251911 | CTB-179K24.3 |
| **438** | 19 | 45592475 | 239 | 19:45592475:C:T | rs9653111 | 1.29E-15 | 7 | rs9653111; rs10413253; rs79322367; rs7247764; rs114783480; rs148023052; rs562297120 | CTB-179K24.3:GEMIN7:MARK4 |
| **439** | 19 | 45605308 | 239 | 19:45605308:C:T | rs12461065 | 9.47E-42 | 9 | rs12461065; rs10419255; rs79322367; rs78073763; rs10421247; rs10416371; rs56413312; rs141012049; rs562297120 | MARK4:PPP1R37 |
| **440** | 19 | 45633686 | 239 | 19:45633686:A:G | rs754366 | 4.43E-71 | 8 | rs754366; rs10416371; rs7247764; rs532649997; rs12460985; rs12461027; rs141012049; rs10413253 | MARK4:PPP1R37 |
| **441** | 19 | 45638505 | 239 | 19:45638505:C:CAAA | 19:45638505_CAAA_C | 2.00E-08 | 2 | 19:45638505_CAAA_C; rs10419255 | MARK4:PPP1R37 |
| **442** | 19 | 45673122 | 239 | 19:45673122:A:G | rs76971643 | 3.31E-12 | 2 | rs76971643; rs78073763 | MARK4:TRAPPC6A |
| **443** | 19 | 45700657 | 239 | 19:45700657:A:C | rs8113128 | 2.49E-26 | 2 | rs8113128; rs2627646 | MARK4:AC005779.2:AC006126.3 |
| **444** | 19 | 45707917 | 239 | 19:45707917:G:T | rs582747 | 6.91E-43 | 5 | rs582747; rs12978573; rs346757; rs12461027; rs620807 | MARK4:AC006126.3 |
| **445** | 19 | 45719790 | 239 | 19:45719790:C:T | rs10415392 | 2.00E-42 | 5 | rs10415392; rs73034893; rs2627646; rs2627642; rs346740 | MARK4:AC006126.3:EXOC3L2 |
| **446** | 19 | 45723706 | 239 | 19:45723706:C:T | rs12461144 | 2.65E-34 | 4 | rs12461144; rs12978573; rs346757; rs346740 | MARK4:EXOC3L2 |
| **447** | 19 | 45734751 | 239 | 19:45734751:A:G | rs62118504 | 4.87E-44 | 8 | rs62118504; rs11667829; 19:45765191_CA_C; rs344811; rs749827442; rs34600364; rs11878815; rs11667509 | MARK4:EXOC3L2 |
| **448** | 19 | 45750833 | 239 | 19:45750833:C:T | rs77345544 | 3.27E-10 | 1 | rs77345544 | MARK4 |
| **449** | 19 | 45813831 | 239 | 19:45813831:C:T | rs344793 | 3.58E-16 | 11 | rs344793; rs11878815; rs1967311; rs344819; rs344818; rs1799783; rs11667829; 19:45765191_CA_C; rs2098088; rs749827442; rs34600364 | CKM |
| **450** | 19 | 45830947 | 239 | 19:45830947:A:G | rs123187 | 8.72E-27 | 20 | rs123187; rs10416849; rs1799793; rs1799783; rs10419090; rs11673653; rs12983892; rs10420725; rs8109532; rs35242315; rs139138946; rs11667829; 19:45765191_CA_C; rs344811; rs749827442; rs34600364; rs11878815; rs344819; rs344818; rs7247937 | CKM |
| **451** | 19 | 45854330 | 239 | 19:45854330:A:C | rs3916898 | 5.45E-11 | 2 | rs3916898; rs10419090 | KLC3:ERCC2 |
| **452** | 19 | 45881981 | 239 | 19:45881981:A:G | rs4544343 | 2.31E-10 | 7 | rs4544343; rs10419090; rs11673653; rs8109532; rs344819; rs1799793; rs1799783 | PPP1R13L |
| **453** | 19 | 45938019 | 239 | 19:45938019:A:T | rs143008566 | 1.07E-18 | 1 | rs143008566 | ERCC1 |
| **454** | 19 | 45952862 | 239 | 19:45952862:A:G | rs34699196 | 5.28E-10 | 4 | rs34699196; rs8109532; rs10416517; rs10402243 | ERCC1 |
| **455** | 19 | 45953074 | 239 | 19:45953074:C:T | rs144057986 | 2.28E-12 | 14 | rs144057986; rs8109532; rs10402243; rs35242315; rs2282695; rs139138946; rs60533566; rs758761; rs758762; rs1799793; rs10419090; rs11673653; rs12983892; rs10420725 | ERCC1 |
| **456** | 19 | 45967369 | 239 | 19:45967369:C:G | rs8110514 | 2.40E-08 | 8 | rs8110514; rs35242315; rs2282695; rs139138946; rs11673653; rs10420725; rs8109532; rs10416517 | ERCC1 |
| **457** | 19 | 46049982 | 239 | 19:46049982:A:G | rs10422253 | 3.35E-25 | 17 | rs10422253; rs11083772; rs62109650; rs74313320; rs55793324; rs4803840; rs1799783; rs10402243; rs35242315; rs2282695; rs139138946; rs60533566; rs1603; rs758761; rs758762; rs12978806; rs62109648 | OPA3 |
| **458** | 19 | 46069895 | 239 | 19:46069895:A:AT | rs576381162 | 1.94E-08 | 1 | rs576381162 | OPA3 |
| **459** | 19 | 46165082 | 239 | 19:46165082:A:G | rs112972879 | 3.47E-22 | 14 | rs112972879; rs8103658; rs55742289; rs2302593; rs10410910; rs17878252; rs2014576; rs11545326; rs4802279; rs55793324; rs12985660; rs4803836; rs4803840; rs12974310 | GIPR |
| **460** | 19 | 46310512 | 239 | 19:46310512:A:C | rs12462616 | 2.34E-08 | 1 | rs12462616 | RSPH6A |
| **461** | 19 | 46358957 | 239 | 19:46358957:A:G | rs113996837 | 3.97E-12 | 9 | rs113996837; rs7248383; rs11671009; rs55742289; rs10410910; rs17878252; rs2014576; rs4802279; rs16980051 | SYMPK |
| **462** | 19 | 46428653 | 239 | 19:46428653:C:T | rs9789319 | 1.63E-18 | 6 | rs9789319; rs10424403; rs11083788; rs9304651; rs7248383; rs11671009 | NOVA2 |
| **463** | 19 | 46451400 | 239 | 19:46451400:A:C | rs57784658 | 1.23E-10 | 2 | rs57784658; rs11671009 | NOVA2 |
| **464** | 20 | 25282967 | 240 | 20:25282967:C:T | rs746748 | 3.80E-09 | 1 | rs746748 | ABHD12 |
| **465** | 20 | 35996787 | 241 | 20:35996787:A:C | rs117481304 | 4.29E-10 | 1 | rs117481304 | SRC |
| **466** | 20 | 36240021 | 241 | 20:36240021:A:G | rs6125601 | 1.59E-10 | 2 | rs6125601; rs59294445 | LINC00489 |
| **467** | 21 | 43066272 | 242 | 21:43066272:A:G | rs2838084 | 1.79E-08 | 1 | rs2838084 | LINC00111 |
| **468** | 22 | 30236395 | 243 | 22:30236395:A:G | rs2097351 | 3.73E-10 | 2 | rs2097351; rs5763593 | ASCC2 |
| **469** | 22 | 41587556 | 244 | 22:41587556:A:T | rs9607782 | 3.14E-18 | 8 | rs9607782; rs6002328; rs2281331; rs169361; rs13054099; rs138337; rs4822000; rs10854741 | RP1-85F18.5 |
| **470** | 22 | 42235352 | 244 | 22:42235352:C:T | rs9607850 | 4.45E-11 | 3 | rs9607850; rs4822000; rs2281331 | SREBF2 |
| **471** | 22 | 42649443 | 244 | 22:42649443:A:G | rs78179954 | 2.15E-13 | 2 | rs78179954; rs149152747 | TCF20 |
| **472** | 22 | 45314246 | 245 | 22:45314246:C:T | rs9614981 | 2.48E-08 | 1 | rs9614981 | PHF21B |
| **473** | 22 | 50193909 | 246 | 22:50193909:C:T | rs11913353 | 1.37E-08 | 1 | rs11913353 | BRD1 |

Variants with *p*-value <= 5x10^-08^ in the UKBB GWAS of PRS. SNP positions are in GRCh37/hg19. Genomic locus is the index of the genomic risk loci defined by independent lead SNPs and maximum distance between their LD block (>250 kb apart), defined according to FUMA. Number of independent significant SNPs in LD with the leading SNP of each genomic locus is indicated in the column “# Independent Significant SNPs in LD”.

**Table S2. Replications in UKBB Extremes GWAS, comparing to previously reported studies from GWAS Catalog.**

| **CHR** | **Position - reported** | **Position - PRS GWAS** | **RsID - reported** | **RsID - PRS GWAS** | **GwasP - reported** | **GwasP - PRS GWAS** | **Nearest gene - reported** | **Study** | **Nearest gene - PRS GWAS** | **OR - PRS GWAS** | **SE - PRS GWAS** |
| --- | --- | --- | --- | --- | --- | --- | --- | --- | --- | --- | --- |
| 1 | 161155392 | 161155392 | rs4575098 | rs4575098 | 2.05E-10 | 6.90E-18 | *ADAMTS4* | Jansen | *ADAMTS4* | 1.162 | 0.017 |
| 1 | 207653395 | 207653395 | rs17617 | rs17617 | 6.32E-11 | 2.60E-18 | *CR2* | Moreno | *CR2* | 1.195 | 0.020 |
| 1 | 207692049 | 207802552 | rs6656401 | rs4844610 | 1.37E-29 | 4.87E-25 | *CR1* | Marioni | *CR1* | 1.222 | 0.019 |
| 1 | 207692049 | 207802552 | rs6656401 | rs4844610 | 2.87E-24 | 4.87E-25 | *CR1* | Moreno | *CR1* | 1.222 | 0.019 |
| 1 | 207692049 | 207802552 | rs6656401 | rs4844610 | 5.70E-24 | 4.87E-25 | *CR1* | Lambert | *CR1* | 1.222 | 0.019 |
| 1 | 207786828 | 207802552 | rs6656401 | rs4844610 | 1.10E-18 | 4.87E-25 | *CR1* | Jansen | *CR1* | 1.222 | 0.019 |
| 1 | 207802552 | 207802552 | rs4844610 | rs4844610 | 3.60E-24 | 4.87E-25 | *CR1* | Kunkle | *CR1* | 1.222 | 0.019 |
| 2 | 127891427 | 127891427 | rs4663105 | rs4663105 | 3.38E-44 | 1.57E-24 | *BIN1* | Jansen | *BIN1* | 1.166 | 0.015 |
| 2 | 127892810 | 127891427 | rs6733839 | rs4663105 | 1.89E-46 | 1.57E-24 | *BIN1* | Moreno | *BIN1* | 1.166 | 0.015 |
| 2 | 127892810 | 127891427 | rs6733839 | rs4663105 | 2.10E-44 | 1.57E-24 | *BIN1* | Kunkle | *BIN1* | 1.166 | 0.015 |
| 2 | 127892810 | 127891427 | rs6733839 | rs4663105 | 2.37E-69 | 1.57E-24 | *BIN1* | Marioni | *BIN1* | 1.166 | 0.015 |
| 2 | 127892810 | 127891427 | rs6733839 | rs4663105 | 6.90E-44 | 1.57E-24 | *BIN1* | Lambert | *BIN1* | 1.166 | 0.015 |
| 2 | 233981912 | 234067884 | rs10933431 | rs36133610 | 3.40E-09 | 4.78E-14 | *INPP5D* | Kunkle | *INPP5D* | 0.894 | 0.015 |
| 2 | 233981912 | 234067884 | rs10933431 | rs36133610 | 8.92E-10 | 4.78E-14 | *INPP5D* | Jansen | *INPP5D* | 0.894 | 0.015 |
| 2 | 234068476 | 234067884 | rs35349669 | rs36133610 | 3.20E-08 | 4.78E-14 | *INPP5D* | Lambert | *INPP5D* | 0.894 | 0.015 |
| 2 | 234068476 | 234067884 | rs35349669 | rs36133610 | 3.58E-11 | 4.78E-14 | *INPP5D* | Marioni | *INPP5D* | 0.894 | 0.015 |
| 6 | 32575406 | 32575319 | rs9271058 | rs9271053 | 1.40E-11 | 1.83E-10 | *HLA-DRB1* | Kunkle | - | 1.111 | 0.017 |
| 6 | 32578530 | 32578632 | rs9271192 | rs3997869 | 2.90E-12 | 1.82E-28 | *HLA-DRB5-HLA-DRB1* | Lambert | - | 0.832 | 0.017 |
| 6 | 32583357 | 32583146 | rs9269853 | rs4959105 | 8.41E-11 | 5.15E-28 | *HLA-DRB1* | Jansen | - | 0.839 | 0.016 |
| 6 | 40942196 | 41140984 | - | rs9394764 | 1.45E-16 | 1.01E-09 | *TREM2* | Jansen | *TREM2* | 0.906 | 0.016 |
| 6 | 41129252 | 41140984 | rs75932628 | rs9394764 | 2.70E-15 | 1.01E-09 | *TREM2* | Kunkle | *TREM2* | 0.906 | 0.016 |
| 6 | 41154650 | 41140984 | rs9381040 | rs9394764 | 1.55E-08 | 1.01E-09 | *TREM2* | Marioni | *TREM2* | 0.906 | 0.016 |
| 6 | 47431284 | 47595155 | rs9473117 | rs1385742 | 1.20E-10 | 7.24E-13 | *CD2AP* | Kunkle | *CD2AP* | 1.118 | 0.016 |
| 6 | 47431284 | 47432637 | rs9473117 | rs9381563 | 2.88E-11 | 6.13E-12 | *AL355353.1* | Moreno | *AL355353.1* | 1.112 | 0.015 |
| 6 | 47432637 | 47595155 | rs9381563 | rs1385742 | 2.52E-10 | 7.24E-13 | *CD2AP* | Jansen | *CD2AP* | 1.118 | 0.016 |
| 6 | 47432637 | 47595155 | rs9381563 | rs1385742 | 5.83E-14 | 7.24E-13 | *CD2AP* | Marioni | *CD2AP* | 1.118 | 0.016 |
| 6 | 47443806 | 47442377 | rs9381564 | rs1931837 | 2.12E-11 | 1.79E-10 | *RP11-385F7.1* | Moreno | *RP11-385F7.1* | 1.111 | 0.016 |
| 6 | 47487762 | 47595155 | rs10948363 | rs1385742 | 1.57E-11 | 7.24E-13 | *CD2AP* | Moreno | *CD2AP* | 1.118 | 0.016 |
| 6 | 47487762 | 47595155 | rs10948363 | rs1385742 | 5.20E-11 | 7.24E-13 | *CD2AP* | Lambert | *CD2AP* | 1.118 | 0.016 |
| 6 | 47627027 | 47621579 | rs4711895 | rs7772202 | 7.23E-10 | 3.76E-09 | *GPR111* | Moreno | *GPR111* | 1.103 | 0.017 |
| 6 | 47656852 | 47653629 | rs7770492 | rs10948375 | 3.74E-08 | 3.01E-08 | *GPR111:GPR115* | Moreno | *GPR111:GPR115* | 1.106 | 0.018 |
| 7 | 99591021 | 99587980 | rs34213597 | rs6465759 | 4.03E-09 | 2.81E-15 | *RP4-604G5.3* | Moreno | *RP4-604G5.3* | 1.125 | 0.015 |
| 7 | 99642745 | 99642745 | rs4424195 | rs4424195 | 2.34E-08 | 2.47E-16 | *ZKSCAN1* | Moreno | *ZKSCAN1* | 0.875 | 0.016 |
| 7 | 99656927 | 99650429 | rs12705070 | rs59261051 | 2.96E-08 | 1.59E-16 | *ZSCAN21* | Moreno | *ZSCAN21* | 0.874 | 0.016 |
| 7 | 99677917 | 99682026 | rs34031156 | rs12705071 | 1.90E-08 | 1.23E-16 | *ZNF3* | Moreno | *ZNF3* | 0.874 | 0.016 |
| 7 | 99703958 | 99703958 | rs2293479 | rs2293479 | 2.09E-08 | 6.92E-18 | *AP4M1* | Moreno | *AP4M1* | 0.870 | 0.016 |
| 7 | 99728790 | 99736059 | rs35991721 | rs11771139 | 2.96E-08 | 6.86E-18 | *AC073842.19* | Moreno | *AC073842.19* | 0.869 | 0.016 |
| 7 | 99840461 | 99843724 | rs866500 | 7:99843724_CT_C | 9.14E-09 | 6.80E-27 | *GATS:GATS* | Moreno | *GATS:GATS* | 0.841 | 0.016 |
| 7 | 99971834 | 100004446 | rs1859788 | rs1476679 | 2.22E-15 | 3.31E-35 | *ZCWPW1* | Jansen | *ZCWPW1* | 0.821 | 0.016 |
| 7 | 99971834 | 99984089 | rs1859788 | rs2906657 | 3.45E-10 | 2.08E-36 | *PILRA* | Moreno | *PILRA* | 0.819 | 0.016 |
| 7 | 100004446 | 100004446 | rs1476679 | rs1476679 | 4.51E-10 | 3.31E-35 | *ZCWPW1* | Moreno | *ZCWPW1* | 0.821 | 0.016 |
| 7 | 100004446 | 100004446 | rs1476679 | rs1476679 | 5.60E-10 | 3.31E-35 | *ZCWPW1* | Lambert | *ZCWPW1* | 0.821 | 0.016 |
| 7 | 100004446 | 100004446 | rs1476679 | rs1476679 | 9.93E-19 | 3.31E-35 | *ZCWPW1/PILRA* | Marioni | *ZCWPW1* | 0.821 | 0.016 |
| 7 | 100079857 | 100079857 | rs6971558 | rs6971558 | 6.39E-10 | 2.74E-33 | *AC092849.1* | Moreno | *AC092849.1* | 0.827 | 0.016 |
| 7 | 100091795 | 100091795 | rs12539172 | rs12539172 | 4.65E-10 | 8.07E-33 | *NYAP1* | Moreno | *NYAP1* | 0.829 | 0.016 |
| 7 | 100091795 | 100091795 | rs12539172 | rs12539172 | 9.30E-10 | 8.07E-33 | *NYAP1* | Kunkle | *NYAP1* | 0.829 | 0.016 |
| 7 | 143099133 | 143104331 | rs10808026 | rs3935067 | 1.13E-14 | 2.09E-11 | *EPHA1* | Marioni | *EPHA1* | 1.107 | 0.015 |
| 7 | 143099133 | 143104331 | rs10808026 | rs3935067 | 1.30E-10 | 2.09E-11 | *EPHA1* | Kunkle | *EPHA1* | 1.107 | 0.015 |
| 7 | 143099133 | 143104331 | rs10808026 | rs3935067 | 1.40E-14 | 2.09E-11 | *EPHA1* | Moreno | *EPHA1* | 1.107 | 0.015 |
| 7 | 143108158 | 143104331 | rs11763230 | rs3935067 | 3.59E-11 | 2.09E-11 | *EPHA1* | Jansen | *EPHA1* | 1.107 | 0.015 |
| 7 | 143110762 | 143104331 | rs11771145 | rs3935067 | 1.10E-13 | 2.09E-11 | *EPHA1* | Lambert | *EPHA1* | 1.107 | 0.015 |
| 7 | 143110762 | 143108158 | rs11771145 | rs7810606 | 2.04E-14 | 3.72E-13 | *EPHA1-AS1* | Moreno | *EPHA1-AS1* | 0.898 | 0.015 |
| 7 | 145950029 | 146254508 | - | rs12154459 | 2.10E-09 | 8.41E-09 | *CNTNAP2* | Jansen | *CNTNAP2* | 0.908 | 0.017 |
| 8 | 27195121 | 27205046 | rs28834970 | 8:27205046_AG_A | 1.04E-14 | 6.52E-19 | *PTK2B* | Moreno | *PTK2B* | 1.146 | 0.015 |
| 8 | 27195121 | 27205046 | rs28834970 | 8:27205046_AG_A | 7.40E-14 | 6.52E-19 | *PTK2B* | Lambert | *PTK2B* | 1.146 | 0.015 |
| 8 | 27219987 | 27205046 | rs73223431 | 8:27205046_AG_A | 6.30E-14 | 6.52E-19 | *PTK2B* | Kunkle | *PTK2B* | 1.146 | 0.015 |
| 8 | 27330286 | 27330096 | rs2741341 | rs2741342 | 1.26E-09 | 1.66E-13 | *CHRNA2* | Moreno | *CHRNA2* | 0.880 | 0.017 |
| 8 | 27448028 | 27413421 | rs6983452 | rs2640725 | 1.02E-11 | 1.56E-09 | *GULOP* | Moreno | *GULOP* | 1.095 | 0.015 |
| 8 | 27464929 | 27476815 | rs4236673 | rs538181 | 1.07E-28 | 1.41E-08 | *CLU* | Marioni | *CLU* | 0.919 | 0.015 |
| 8 | 27467686 | 27476815 | rs9331896 | rs538181 | 1.17E-25 | 1.41E-08 | *CLU* | Moreno | *CLU* | 0.919 | 0.015 |
| 8 | 27467686 | 27476815 | rs9331896 | rs538181 | 2.80E-25 | 1.41E-08 | *CLU* | Lambert | *CLU* | 0.919 | 0.015 |
| 8 | 27467686 | 27476815 | rs9331896 | rs538181 | 4.60E-24 | 1.41E-08 | *CLU* | Kunkle | *CLU* | 0.919 | 0.015 |
| 8 | 27486916 | 27485120 | rs576748 | rs525716 | 8.80E-09 | 2.65E-09 | *SCARA3* | Moreno | *SCARA3* | 0.883 | 0.021 |
| 10 | 11717397 | 11718713 | rs11257242 | rs7912495 | 1.26E-08 | 9.20E-09 | *ECHDC3* | Jansen | *RP11-138I18.2* | 1.089 | 0.015 |
| 10 | 11720308 | 11718713 | rs7920721 | rs7912495 | 2.30E-09 | 9.20E-09 | *ECHDC3* | Kunkle | *RP11-138I18.2* | 1.089 | 0.015 |
| 10 | 11720308 | 11718713 | rs7920721 | rs7912495 | 3.17E-11 | 9.20E-09 | *RP11-138I18.2* | Marioni | *RP11-138I18.2* | 1.089 | 0.015 |
| 11 | 47380340 | 47391948 | rs3740688 | rs10437655 | 1.35E-09 | 8.54E-37 | *SPI1* | Moreno | *SPI1* | 1.213 | 0.015 |
| 11 | 47380340 | 47391948 | rs3740688 | rs10437655 | 5.40E-13 | 8.54E-37 | *SPI1* | Kunkle | *SPI1* | 1.213 | 0.015 |
| 11 | 47419663 | 47407861 | rs1534576 | rs768890137 | 3.20E-09 | 2.06E-36 | *RP11-750H9.5* | Moreno | *RP11-750H9.5* | 1.211 | 0.015 |
| 11 | 47429904 | 47429904 | rs7947450 | rs7947450 | 3.39E-09 | 1.71E-35 | *RP11-750H9.5:SLC39A13* | Moreno | *RP11-750H9.5:SLC39A13* | 1.208 | 0.015 |
| 11 | 47432725 | 47432725 | rs10742802 | rs10742802 | 4.55E-09 | 1.91E-35 | *SLC39A13* | Moreno | *SLC39A13* | 1.207 | 0.015 |
| 11 | 47449072 | 47447346 | rs12292911 | rs10838709 | 1.63E-09 | 1.34E-35 | *PSMC3* | Moreno | *PSMC3* | 1.208 | 0.015 |
| 11 | 47449072 | 47391948 | rs12292911 | rs10437655 | 3.30E-09 | 8.54E-37 | *SPI1* | Marioni | *SPI1* | 1.213 | 0.015 |
| 11 | 47461783 | 47461783 | rs7103648 | rs7103648 | 3.98E-09 | 1.74E-35 | *RAPSN* | Moreno | *RAPSN* | 1.207 | 0.015 |
| 11 | 47557871 | 47568344 | rs10838725 | rs10838726 | 1.10E-08 | 4.01E-29 | *CELF1* | Lambert | *CELF1* | 1.198 | 0.016 |
| 11 | 47557871 | 47568344 | rs10838725 | rs10838726 | 3.96E-09 | 4.01E-29 | *CELF1* | Moreno | *CELF1* | 1.198 | 0.016 |
| 11 | 47662932 | 47662932 | rs7120548 | rs7120548 | 2.45E-08 | 9.84E-30 | *MTCH2* | Moreno | *MTCH2* | 1.201 | 0.016 |
| 11 | 47695840 | 47700168 | rs11039332 | rs147311748 | 4.03E-08 | 1.54E-31 | *AGBL2* | Moreno | *AGBL2* | 1.210 | 0.016 |
| 11 | 59837097 | 59826677 | rs528823 | rs1286289 | 4.58E-09 | 1.52E-13 | *MS4A3* | Moreno | *MS4A3* | 0.889 | 0.016 |
| 11 | 59846504 | 59848232 | rs549651 | rs1151105 | 2.27E-09 | 4.26E-12 | *RP11-736I10.2* | Moreno | *RP11-736I10.2* | 0.895 | 0.016 |
| 11 | 59859609 | 59857581 | rs2847667 | rs2583476 | 1.37E-17 | 9.66E-17 | *MS4A2* | Moreno | *MS4A2* | 0.883 | 0.015 |
| 11 | 59923508 | 59928672 | rs983392 | rs11605427 | 1.81E-18 | 9.07E-21 | *AP001257.1* | Moreno | *AP001257.1* | 0.869 | 0.015 |
| 11 | 59923508 | 59959969 | rs983392 | rs1834551 | 6.10E-16 | 6.63E-21 | *MS4A6A* | Lambert | *MS4A6A* | 0.869 | 0.015 |
| 11 | 59936926 | 59857581 | rs7933202 | rs2583476 | 1.90E-19 | 9.66E-17 | *MS4A2* | Kunkle | *MS4A2* | 0.883 | 0.015 |
| 11 | 59936926 | 59959969 | rs7933202 | rs1834551 | 4.73E-18 | 6.63E-21 | *MS4A6A* | Moreno | *MS4A6A* | 0.869 | 0.015 |
| 11 | 59958380 | 59959969 | rs7935829 | rs1834551 | 1.55E-15 | 6.63E-21 | *MS4A6A* | Jansen | *MS4A6A* | 0.869 | 0.015 |
| 11 | 60021948 | 60021948 | rs1582763 | rs1582763 | 1.01E-18 | 1.05E-20 | *MS4A4A/MS4A4E* | Marioni | *MS4A4E* | 0.868 | 0.015 |
| 11 | 60021948 | 59968705 | rs1582763 | rs367670643 | 1.26E-17 | 2.36E-21 | *MS4A4E* | Moreno | *MS4A4E* | 0.867 | 0.015 |
| 11 | 60076940 | 60033371 | rs11824773 | rs7930318 | 7.98E-16 | 2.66E-18 | *MS4A4A* | Moreno | *MS4A4A* | 0.877 | 0.015 |
| 11 | 60099225 | 60097777 | rs4939338 | rs749908546 | 1.02E-15 | 1.75E-18 | *MS4A6E* | Moreno | *MS4A6E* | 0.875 | 0.015 |
| 11 | 85655105 | 85654002 | rs1237230 | rs71036497 | 5.51E-11 | 1.05E-19 | *SLC25A1P1* | Moreno | *SLC25A1P1* | 0.849 | 0.018 |
| 11 | 85776544 | 85788351 | rs10792832 | rs541458 | 2.19E-18 | 6.72E-30 | *PICALM* | Jansen | *PICALM* | 0.835 | 0.016 |
| 11 | 85776544 | 85788351 | rs867611 | rs541458 | 2.30E-21 | 6.72E-30 | *PICALM* | Moreno | *PICALM* | 0.835 | 0.016 |
| 11 | 85831541 | 85800279 | rs471470 | rs561655 | 1.29E-25 | 1.38E-30 | *snoU13* | Moreno | *snoU13* | 0.837 | 0.015 |
| 11 | 85867875 | 85867875 | rs10792832 | rs10792832 | 1.32E-27 | 6.03E-31 | *RNU6-560P* | Moreno | *RNU6-560P* | 0.838 | 0.015 |
| 11 | 85867875 | 85788351 | rs10792832 | rs541458 | 5.08E-36 | 6.72E-30 | *PICALM* | Marioni | *PICALM* | 0.835 | 0.016 |
| 11 | 85867875 | 85788351 | rs10792832 | rs541458 | 9.30E-26 | 6.72E-30 | *PICALM* | Lambert | *PICALM* | 0.835 | 0.016 |
| 11 | 85868640 | 85788351 | rs3851179 | rs541458 | 6.00E-25 | 6.72E-30 | *PICALM* | Kunkle | *PICALM* | 0.835 | 0.016 |
| 14 | 53298277 | 53268857 | rs12586707 | rs7154989 | 1.01E-08 | 1.28E-10 | *RP11-589M4.3* | Moreno | *RP11-589M4.3* | 0.909 | 0.015 |
| 14 | 92926952 | 92845636 | rs10498633 | rs35627364 | 1.69E-08 | 1.59E-10 | *SLC24A4* | Moreno | *SLC24A4* | 0.900 | 0.016 |
| 14 | 92926952 | 92845636 | rs10498633 | rs35627364 | 5.50E-09 | 1.59E-10 | *SLC24A4* | Lambert | *SLC24A4* | 0.900 | 0.016 |
| 14 | 92932828 | 92845636 | rs12881735 | rs35627364 | 7.40E-09 | 1.59E-10 | *SLC24A4* | Kunkle | *SLC24A4* | 0.900 | 0.016 |
| 14 | 92938855 | 92845636 | rs12590654 | rs35627364 | 1.65E-10 | 1.59E-10 | *SLC24A4* | Jansen | *SLC24A4* | 0.900 | 0.016 |
| 14 | 92938855 | 92938855 | rs12590654 | rs12590654 | 8.21E-12 | 1.29E-08 | *SLC24A4* | Marioni | *SLC24A4* | 0.915 | 0.016 |
| 15 | 51001534 | 51057868 | rs59685680 | rs3896609 | 9.17E-09 | 3.49E-11 | *SPPL2A* | Marioni | *SPPL2A* | 0.881 | 0.019 |
| 15 | 59022615 | 59042012 | rs442495 | rs653765 | 1.31E-09 | 3.37E-13 | *ADAM10* | Jansen | *ADAM10* | 0.888 | 0.016 |
| 15 | 59045774 | 59042012 | rs593742 | rs653765 | 2.78E-11 | 3.37E-13 | *ADAM10* | Marioni | *ADAM10* | 0.888 | 0.016 |
| 15 | 63569902 | 63596440 | rs117618017 | rs28386949 | 3.35E-08 | 1.15E-09 | *APH1B* | Jansen | *APH1B* | 0.891 | 0.019 |
| 16 | 31122571 | 31112810 | rs889555 | rs7187995 | 4.11E-08 | 6.03E-09 | *BCKDK/KAT8* | Marioni | *BCKDK* | 1.094 | 0.015 |
| 16 | 31133100 | 31132662 | rs59735493 | rs28725459 | 3.98E-08 | 4.31E-09 | *KAT8* | Jansen | *KAT8* | 0.908 | 0.016 |
| 16 | 70694000 | 70676478 | rs4985556 | rs12598456 | 3.67E-08 | 2.52E-21 | *IL34* | Marioni | *IL34* | 1.158 | 0.015 |
| 16 | 70694000 | 70676478 | rs4985556 | rs12598456 | 3.91E-10 | 2.52E-21 | *IL34* | Moreno | *IL34* | 1.158 | 0.015 |
| 17 | 5137047 | 5137047 | rs7225151 | rs7225151 | 1.12E-08 | 1.23E-09 | *RP11-333E1.1:SCIMP* | Moreno | *RP11-333E1.1:SCIMP* | 1.148 | 0.023 |
| 17 | 5137047 | 5137047 | rs7225151 | rs7225151 | 6.06E-12 | 1.23E-09 | *SCIMP* | Marioni | *RP11-333E1.1:SCIMP* | 1.148 | 0.023 |
| 17 | 5138980 | 5138980 | rs113260531 | rs113260531 | 9.16E-10 | 8.13E-10 | *SCIMP* | Jansen | *RP11-333E1.1* | 1.150 | 0.023 |
| 17 | 5140399 | 5105303 | rs4456560 | rs78538460 | 1.75E-08 | 2.48E-12 | *RP11-333E1.1* | Moreno | *RP11-333E1.1* | 1.239 | 0.031 |
| 17 | 47450775 | 47316259 | rs28394864 | rs850522 | 1.87E-08 | 6.57E-10 | *ABI3* | Jansen | *PHOSPHO1* | 0.900 | 0.017 |
| 17 | 61538148 | 61560763 | rs138190086 | rs4311 | 1.95E-09 | 2.72E-10 | *ACE* | Marioni | *ACE* | 0.910 | 0.015 |
| 17 | 61538148 | 61560763 | rs138190086 | rs4311 | 7.50E-09 | 2.72E-10 | *ACE* | Kunkle | *ACE* | 0.910 | 0.015 |
| 19 | 1028149 | 1039323 | rs75364577 | rs111278892 | 1.07E-10 | 1.18E-11 | *CNN2* | Moreno | *CNN2* | 1.148 | 0.020 |
| 19 | 1039323 | 1043638 | rs4147929 | rs3752231 | 7.93E-11 | 1.84E-12 | *ABCA7* | Jansen | *ABCA7* | 1.127 | 0.017 |
| 19 | 1043638 | 1039323 | rs3752231 | rs111278892 | 4.37E-13 | 1.18E-11 | *CNN2* | Marioni | *CNN2* | 1.148 | 0.020 |
| 19 | 1056492 | 1043638 | rs3752246 | rs3752231 | 3.10E-16 | 1.84E-12 | *ABCA7* | Kunkle | *ABCA7* | 1.127 | 0.017 |
| 19 | 1063443 | 1043638 | rs4147929 | rs3752231 | 1.10E-15 | 1.84E-12 | *ABCA7* | Lambert | *ABCA7* | 1.127 | 0.017 |
| 19 | 1063443 | 1043638 | rs4147929 | rs3752231 | 1.20E-16 | 1.84E-12 | *ABCA7* | Moreno | *ABCA7* | 1.127 | 0.017 |
| 19 | 45351516 | 45413576 | rs41289512 | rs75627662 | 5.79E-276 | 5.19E-259 | *APOE* | Jansen | *APOE* | 1.875 | 0.018 |
| 19 | 45351516 | 45413576 | rs41289512 | rs75627662 | 6.70E-255 | 5.19E-259 | *APOE* | Marioni | *APOE* | 1.875 | 0.018 |
| 19 | 46241841 | 46267453 | rs76320948 | rs16980013 | 4.64E-08 | 5.80E-20 | *AC074212.3* | Jansen | *AC074212.3* | 1.157 | 0.016 |

Comparison between locus found previously reported in studies and replicated in PRS extremes GWAS. Studies used for Comparison were Jansen et *al*., Kunkle et *al.*, Lambert et *al.*, Marioni et *al.*, Moreno et *al.*, Scelsi et *al.*, from GWAS Catalog and are indicated in “Study”. Variants with *p*-value <= 5x10^-08^ in these studies were considered. SNP positions are in GRCh37/hg19.

**Table S3. Significant loci in previously reported studies from GWAS Catalog and were not replicated in UKBB Extremes GWAS.**

| **CHR** | **Position** | **RsID** | **GwasP** | **Nearest gene** | **Study** | **Start** | **End** | **Reason** | **Extra notes** |
| --- | --- | --- | --- | --- | --- | --- | --- | --- | --- |
| 2 | 37515958 | rs876461 | 1.34E-09 | *PRKD3/ NDUFAF7* | Moreno | 37458774 | 37324833 | Only two variants were detected in the PRS GWAS | Likely a false positive in PRS GWAS study |
| 3 | 57226150 | - | 1.24E-08 | *HESX1* | Jansen | 57225900 | 57226400 | Excluded due to very low frequency (MAF=0.0016) | Near *SLMAP* locus which is GWS |
| 4 | 11026028 | rs6448453 | 1.93E-09 | *CLNK* | Jansen | 11025778 | 11026278 | Borderline significant:  *p*-value=4.75E-07 |  |
| 4 | 11027619 | rs4351014 | 9.16E-12 | *HS3ST1* | Moreno | 11027369 | 11027869 | Borderline significant:  *p*-value=1.02E-06 |  |
| 4 | 11723235 | rs7657553 | 2.16E-08 | *HS3ST1* | Jansen | 11722985 | 11723485 | Not GWS |  |
| 4 | 18006089 | rs6850306 | 1.03E-08 | *LCORL* | Scelsi | 18005839 | 18006339 | Not GWS |  |
| 5 | 74368254 | rs4704171 | 2.78E-08 | *ANKRD31* | Moreno | 74368004 | 74368504 | Not GWS |  |
| 5 | 88223420 | rs190982 | 8.75E-09 | *MEF2C-AS1* | Moreno | 88223170 | 88223670 | Excluded due to missing call rates (geno = 0.057) |  |
| 5 | 88223420 | rs190982 | 3.20E-08 | *MEF2C* | Lambert | 88223170 | 88223670 | Excluded due to missing call rates (geno = 0.057) |  |
| 6 | 47379843 | rs9369686 | 6.27E-09 | *RP11-157D6.1* | Moreno | 47379593 | 47380093 | *p*-value=1.30E-05 | Near *CD2AP* which we replicate |
| 7 | 37841534 | rs2718058 | 3.90E-08 | *GPR141:EPDR1* | Moreno | 37841284 | 37841784 | Not GWS |  |
| 7 | 37841534 | rs2718058 | 4.80E-09 | *NME8* | Lambert | 37841284 | 37841784 | Not GWS |  |
| 8 | 95955074 | rs4735333 | 2.57E-08 | *NDUFAF6:TP53INP1* | Moreno | 95954824 | 95955324 | Not GWS |  |
| 8 | 145158607 | rs34173062 | 9.62E-13 | *SHARPIN* | Moreno | 145158357 | 145158857 | Excluded due to missing call rates (geno = 0.059) |  |
| 11 | 121435587 | rs11218343 | 1.09E-11 | *SORL1* | Jansen | 121435337 | 121435837 | Excluded due to very low frequency (MAF=0.0377) |  |
| 11 | 121435587 | rs11218343 | 2.90E-12 | *SORL1* | Kunkle | 121435337 | 121435837 | Excluded due to very low frequency (MAF=0.0377) |  |
| 11 | 121435587 | rs11218343 | 4.58E-17 | *SORL1* | Marioni | 121435337 | 121435837 | Excluded due to very low frequency (MAF=0.0377) |  |
| 11 | 121435587 | rs11218343 | 9.70E-15 | *SORL1* | Lambert | 121435337 | 121435837 | Excluded due to very low frequency (MAF=0.0377) |  |
| 11 | 121435587 | rs11218343 | 5.25E-14 | *SORL1* | Moreno | 121435337 | 121435837 | Excluded due to very low frequency (MAF=0.0377) |  |
| 14 | 53298277 | rs12586707 | 1.01E-08 | *RP11-589M4.3* | Moreno | 53298027 | 53298527 | Not GWS |  |
| 14 | 53391680 | rs17125924 | 1.34E-11 | *FERMT2* | Marioni | 53391430 | 53391930 | Borderline significant:  *p*-value=6.80E-07 |  |
| 14 | 53391680 | rs17125924 | 1.40E-09 | *FERMT2* | Kunkle | 53391430 | 53391930 | Borderline significant:  *p*-value=6.80E-08 |  |
| 14 | 53400629 | rs17125944 | 7.90E-09 | *FERMT2* | Lambert | 53400379 | 53400879 | Borderline significant:  *p*-value=9.45E-07 |  |
| 14 | 53400629 | rs17125944 | 8.07E-10 | *FERMT2* | Moreno | 53400379 | 53400879 | Borderline significant:  *p*-value=9.45E-07 |  |
| 16 | 81773209 | rs12444183 | 3.15E-08 | *PLCG2* | Marioni | 81772959 | 81773459 | Not GWS |  |
| 16 | 81773209 | rs12444183 | 6.81E-12 | *PLCG2* | Moreno | 81772959 | 81773459 | Not GWS |  |
| 17 | 4805437 | rs72835061 | 1.51E-10 | *CHRNE* | Moreno | 4805187 | 4805687 | *p*-value=1.46E-05 | Near *SCIMP* locus which we replicate |
| 17 | 56409089 | rs2632516 | 1.42E-09 | *BZRAP1-AS1* | Jansen | 56408839 | 56409339 | Borderline significant:  *p*-value=3.68E-06 |  |
| 18 | 29088958 | rs8093731 | 4.63E-08 | *SUZ12P1* | Jansen | 29088708 | 29089208 | Excluded due to very low frequency (MAF=0.0116) | From "Summary statistics and functional annotation for SNPS reaching genome-wide significance in the GRACE dementia with dbGaP" |
| 18 | 29088958 | rs8093731 | 4.63E-08 | *SUZ12P1/ DSG2* | Lambert | 29088708 | 29089208 | Excluded due to very low frequency (MAF=0.0116) |  |
| 18 | 56189459 | rs76726049 | 3.30E-08 | *ALPK2* | Jansen | 56189209 | 56189709 | Excluded due to very low frequency (MAF=0.0116) |  |
| 19 | 1021627 | rs117481827 | 1.55E-08 | *RNU6-2* | Moreno | 1021377 | 1021877 | ABCA7 Region |  |
| 19 | 1073073 | rs2072102 | 2.65E-10 | *HMHA1* | Moreno | 1072823 | 1073323 | Not GWS |  |
| 19 | 51727962 | rs3865444 | 6.34E-09 | *CD33* | Jansen | 51727712 | 51728212 | Not GWS |  |
| 19 | 51728477 | rs12459419 | 7.97E-09 | *CD33* | Marioni | 51728227 | 51728727 | *p*-value=3.74E-05 |  |
| 20 | 54983075 | rs6069736 | 2.00E-10 | *CASS4* | Marioni | 54982825 | 54983325 | Not GWS |  |
| 20 | 54997568 | rs6024870 | 3.50E-08 | *CASS4* | Kunkle | 54997318 | 54997818 | Not GWS |  |
| 20 | 54998544 | rs6014724 | 6.56E-10 | *CASS4* | Jansen | 54998294 | 54998794 | *p*-value=4.74E-05 |  |
| 20 | 55018260 | rs7274581 | 2.50E-08 | *CASS4* | Lambert | 55018010 | 55018510 | Not GWS |  |
| 21 | 27473875 | rs2154481 | 1.39E-11 | *APP* | Moreno | 27473625 | 27474125 | Not GWS |  |

Comparison between locus found previously reported in studies and not replicated in PRS extremes GWAS. Studies used for Comparison were Jansen et *al*., Kunkle et *al.*, Lambert et *al.*, Marioni et *al.*, Moreno et *al.*, Scelsi et *al.*, from GWAS Catalog and are indicated in “Study”. Variants with *p*-value <= 5x10^-08^ in these studies were considered. SNP positions are in GRCh37/hg19. We indicate the reasons why these SNPs were not replicated and extra notes when considered relevant.

**Table S4. Traits from GWAS Catalog with overlapping genes in the PRS extremes GWAS.**

| **Category** | **# Genes in GWAS Catalog reported trait** | **# Overlapping genes in our GWAS** | ***p*-value** | **Adjusted *p*-value** | **Genes** |
| --- | --- | --- | --- | --- | --- |
| Autism spectrum disorder or schizophrenia | 474 | 183 | 2.16E-96 | 3.92E-93 | *CREB3L1:DGKZ:MDK:CHRM4:AMBRA1:HARBI1:ATG13:ARHGAP1:ZNF408:F2:CKAP5:LRP4:C11orf49:ARFGAP2:PACSIN3:DDB2:ACP2:NR1H3:MADD:MYBPC3:ABCB9:OGFOD2:ARL6IP4:PITPNM2:MPHOSPH9:C12orf65:CDK2AP1:SBNO1:SETD8:RILPL2:ETFA:TMEM219:TAOK2:HIRIP3:INO80E:DOC2A:C16orf92:FAM57B:ALDOA:PPP4C:TBX6:YPEL3:GDPD3:MAPK3:SLC52A1:ZNF804A:MCHR1:SLC25A17:ST13:XPNPEP3:DNAJB7:RBX1:EP300:L3MBTL2:CHADL:RANGAP1:ZC3H7B:TEF:TOB2:SEPT3:WBP2NL:NAGA:CYP2D6:TCF20:PPP2R3A:MSL2:PCCB:STAG1:SLC35G2:NCK1:IL20RB:HCN1:ELOVL7:NDUFAF2:SMIM15:ZSWIM6:GALNT10:SCGN:SLC17A4:SLC17A1:SLC17A3:SLC17A2:TRIM38:HIST1H1A:HIST1H3A:HIST1H4A:HIST1H4B:HIST1H3B:HIST1H2AB:HIST1H2BB:HIST1H3C:HIST1H1C:HFE:HIST1H4C:HIST1H1T:HIST1H2BC:HIST1H2AC:HIST1H1E:HIST1H2BD:HIST1H2BE:HIST1H4D:HIST1H3D:HIST1H2AD:HIST1H2BF:HIST1H4E:HIST1H3E:HIST1H1D:HIST1H3G:HIST1H2BI:BTN3A2:BTN2A2:BTN3A1:BTN3A3:BTN2A1:BTN1A1:HMGN4:ABT1:ZNF322:HIST1H2BJ:HIST1H2AG:HIST1H2BK:HIST1H4I:HIST1H2AH:PRSS16:POM121L2:ZNF391:ZNF184:HIST1H2BL:HIST1H2AI:HIST1H3H:HIST1H2AJ:HIST1H2BM:HIST1H4J:HIST1H4K:HIST1H2AK:HIST1H2BN:HIST1H2AL:HIST1H1B:HIST1H3I:HIST1H4L:HIST1H3J:HIST1H2AM:HIST1H2BO:OR2B2:OR2B6:ZNF165:ZSCAN16:ZKSCAN8:ZSCAN9:ZKSCAN4:NKAPL:PGBD1:ZSCAN31:ZKSCAN3:ZSCAN12:ZSCAN23:GPX6:GPX5:SCAND3:TRIM27:C6orf100:ZNF311:OR2W1:OR2B3:OR2J3:OR2J2:OR14J1:OR5V1:OR12D3:OR12D2:OR11A1:OR10C1:OR2H1:MAS1L:UBD:GABBR1:OR2H2:RIMS1:PTK2B:CHRNA2:EPHX2:CLU:SCARA3* |
| Schizophrenia | 704 | 158 | 3.47E-46 | 3.15E-43 | *CR1L:NRP1:CREB3L1:DGKZ:MDK:CHRM4:AMBRA1:HARBI1:ATG13:ARHGAP1:ZNF408:F2:CKAP5:DLG2:FEZ1:STT3A:ATP2A2:HIP1R:ABCB9:OGFOD2:ARL6IP4:PITPNM2:MPHOSPH9:C12orf65:CDK2AP1:SBNO1:SETD8:RILPL2:FLRT2:LIPC:ADAM10:CPEB1:AP3B2:TMEM219:TAOK2:HIRIP3:INO80E:DOC2A:C16orf92:FAM57B:ALDOA:PPP4C:TBX6:YPEL3:GDPD3:MAPK3:RLTPR:ACD:PARD6A:ENKD1:C16orf86:GFOD2:RANBP10:TSNAXIP1:SLC52A1:RAI1:SREBF1:TOM1L2:LRRC48:ATPAF2:GID4:DRG2:MYO15A:AKAP10:SPECC1:CLC:KCNN4:PRKD3:FBXO11:ZNF804A:MCHR1:XPNPEP3:EP300:L3MBTL2:CHADL:RANGAP1:ZC3H7B:POLR3H:CSDC2:SREBF2:SEPT3:WBP2NL:NAGA:CYP2D6:TCF20:BRD1:GNAT1:HYAL3:RASSF1:ROBO1:PPP2R3A:MSL2:PCCB:STAG1:SLC35G2:NCK1:NDST3:HCN1:SMIM15:ZSWIM6:CTNNA1:SRA1:CD14:NDUFA2:TMCO6:IK:WDR55:DND1:HARS:HARS2:ZMAT2:PCDHA1:PCDHA2:PCDHA3:PCDHA4:PCDHA5:PCDHA6:PCDHA7:PCDHA8:PCDHA9:PCDHA10:PCDHA13:PCDHAC2:GRIA1:GALNT10:SLC17A4:SLC17A1:SLC17A3:HFE:HIST1H3D:BTN3A2:BTN2A2:BTN3A1:HIST1H2BJ:HIST1H2AG:PRSS16:POM121L2:ZNF184:HIST1H2AJ:ZKSCAN4:NKAPL:ZSCAN31:ZKSCAN3:ZSCAN23:TRIM27:ZNF311:OR12D3:UBD:GABBR1:PHF3:EYS:RIMS1:POP7:PSD3:PTK2B:CHRNA2:EPHX2:CLU* |
| **Alzheimer's disease or HDL levels (pleiotropy)** | **53** | **41** | **7.30E-39** | **4.41E-36** | ***PICALM:LIPC:IGSF23:PVR:CEACAM19:CEACAM16:BCL3:CBLC:BCAM:PVRL2:TOMM40:APOE:APOC1:APOC4-APOC2:APOC4:APOC2:CLPTM1:RELB:CLASRP:ZNF296:GEMIN7:MARK4:PPP1R37:NKPD1:TRAPPC6A:BLOC1S3:EXOC3L2:CKM:KLC3:ERCC2:PPP1R13L:CD3EAP:ERCC1:FOSB:RTN2:PPM1N:VASP:OPA3:BIN1:ZYX:EPHA1*** |
| Alzheimer's disease or fasting glucose levels (pleiotropy) | 45 | 37 | 5.10E-37 | 2.31E-34 | *CREB3L1:DGKZ:MDK:CHRM4:AMBRA1:HARBI1:ATG13:ARHGAP1:ZNF408:F2:CKAP5:LRP4:C11orf49:ARFGAP2:PACSIN3:DDB2:ACP2:NR1H3:MADD:MYBPC3:SPI1:SLC39A13:PSMC3:RAPSN:CELF1:NDUFS3:PTPMT1:KBTBD4:KBTBD4:FAM180B:C1QTNF4:MTCH2:AGBL2:FNBP4:NUP160:PTPRJ:OR4S1:ETFA* |
| **Alzheimer's disease or family history of Alzheimer's disease** | **48** | **38** | **8.49E-37** | **3.08E-34** | ***ADAMTS4:CR1:MS4A6A:PICALM:SLC24A4:ADAM10:APH1B:KAT8:SCIMP:ABI3:ABCA7:IGSF23:PVR:BCL3:CBLC:BCAM:PVRL2:TOMM40:APOE:CLPTM1:RELB:CLASRP:GEMIN7:MARK4:NKPD1:EXOC3L2:CKM:AC074212.3:BIN1:INPP5D:CD2AP:STAG3:ZCWPW1:EPHA1:CNTNAP2:PTK2B:CHRNA2:CLU*** |
| Sleep duration (short sleep) | 94 | 44 | 8.16E-28 | 2.47E-25 | *PSMC3:RAPSN:CELF1:NDUFS3:KBTBD4:KBTBD4:C1QTNF4:MTCH2:AGBL2:FNBP4:NUP160:PTPRJ:USP4:BSN:APEH:MST1:RNF123:AMIGO3:GMPPB:IP6K1:FAM212A:UBA7:TRAIP:CAMKV:MST1R:MON1A:RBM6:RBM5:SEMA3F:GNAT1:GNAI2:LSMEM2:IFRD2:HYAL3:NAT6:HYAL1:HYAL2:TUSC2:RASSF1:CYB561D2:TMEM115:CACNA2D2:ZSCAN31:ZKSCAN3:ZSCAN12* |
| Neuroticism | 138 | 48 | 2.74E-23 | 7.10E-21 | *CACNA1E:ENAH:DDB2:ACP2:NR1H3:MADD:MYBPC3:SPI1:SLC39A13:PSMC3:RAPSN:CELF1:NDUFS3:PTPMT1:KBTBD4:KBTBD4:FAM180B:C1QTNF4:MTCH2:AGBL2:FNBP4:NUP160:ETFA:ARHGAP27:PLEKHM1:CRHR1:SPPL2C:MAPT:STH:KANSL1:ZNF804A:RBX1:EP300:L3MBTL2:CHADL:RANGAP1:ZC3H7B:TEF:TOB2:PHF5A:ACO2:POLR3H:CSDC2:PMM1:DESI1:XRCC6:NHP2L1:C22orf46:MEI1* |
| **Family history of Alzheimer's disease** | **26** | **20** | **1.79E-19** | **4.07E-17** | ***ADAMTS4:CR1:MS4A6A:PICALM:SLC24A4:ADAM10:APH1B:KAT8:SCIMP:ABI3:ABCA7:APOE:BIN1:INPP5D:CD2AP:ZCWPW1:EPHA1:CNTNAP2:PTK2B:CLU*** |
| **Alzheimer's disease (late onset)** | **83** | **33** | **1.71E-18** | **3.45E-16** | ***CR1:F2:CKAP5:CELF1:MS4A6A:MS4A4A:PICALM:SLC24A4:SPPL2A:ADAM10:WWOX:SCIMP:ABI3:ACE:ACE:GRIN3B:ABCA7:HMHA1:CEACAM16:BCL3:PVRL2:TOMM40:APOE:PPP1R37:BIN1:INPP5D:OARD1:TREML2:CD2AP:ZCWPW1:EPHA1:CNTNAP2:PTK2B:CLU*** |
| **Body mass index x age interaction** | **37** | **22** | **1.65E-17** | **3.00E-15** | ***ZNF229:ZNF180:IGSF23:PVR:BCL3:CBLC:BCAM:PVRL2:TOMM40:APOE:APOC1:CLPTM1:RELB:CLASRP:GEMIN7:MARK4:PPP1R37:BLOC1S3:EXOC3L2:CKM:KLC3:ERCC1*** |
| **Body mass index** | **1192** | **153** | **2.88E-17** | **4.75E-15** | ***ECE1:MACF1:PRDX1:AKR1A1:NASP:CCDC17:GPBP1L1:TMEM69:IPP:MAST2:PIK3R3:GIPC2:GOLPH3L:HORMAD1:CTSS:CTSK:SETDB1:BNIPL:GABPB2:PIP5K1A:PSMD4:ZNF687:PI4KB:PSMB4:POGZ:AMBRA1:CELF1:NDUFS3:MTCH2:AGBL2:FNBP4:NUP160:OR4A5:OR4C15:DLG2:EI24:CPNE8:TCTN1:BRAP:ACAD10:ALDH2:MAPKAPK5:TMEM116:ERP29:NAA25:TRAFD1:HECTD4:RPL6:PTPN11:RPH3A:CLIP1:HIP1R:TAOK2:INO80E:ZNF646:KAT8:NOB1:WWP2:CLEC18A:CALB2:ZFHX3:C17orf107:RABEP1:RPAIN:RAI1:AKAP10:NSF:WNT3:ZNF229:ZNF180:IGSF23:PVR:BCL3:CBLC:BCAM:PVRL2:TOMM40:APOE:APOC1:CLPTM1:RELB:CLASRP:GEMIN7:MARK4:PPP1R37:BLOC1S3:EXOC3L2:CKM:KLC3:CD3EAP:ERCC1:GIPR:QPCTL:PRKD3:STON1-GTF2A1L:LHCGR:NRXN1:EHBP1:TMEM163:ACMSD:CCNT2:MAP3K19:CALCRL:ENTPD6:MCHR1:TCF20:BSN:APEH:MST1:RNF123:IP6K1:CDHR4:FAM212A:UBA7:MST1R:MON1A:RBM6:RBM5:SEMA3F:GNAT1:GNAI2:LSMEM2:IFRD2:HYAL3:HYAL2:ERC2:CADM2:STAG1:SLC35G2:NCK1:ZNF131:ZSWIM6:NRG2:IGIP:CYSTM1:PFDN1:GALNT10:BTN1A1:OR5V1:C6orf106:UHRF1BP1:EYS:FAM220A:RAC1:DAGLB:KDELR2:LSM5:AVL9:SBDS:AUTS2:CNTNAP2:FRRS1L:EPB41L4B*** |
| Sarcoidosis (Lofgren's syndrome vs non-Lofgren's syndrome) | 11 | 11 | 7.01E-14 | 1.02E-11 | *ZNF184:ZNF165:ZKSCAN3:ZSCAN12:TRIM27:C6orf100:OR2B3:OR2J3:OR12D3:OR12D2:MAS1L* |
| Lung cancer in ever smokers | 107 | 32 | 7.28E-14 | 1.02E-11 | *GIPC2:TP53BP1:STRC:FSHR:NRXN1:SCGN:TRIM38:HIST1H1A:HIST1H2AC:HIST1H1E:BTN3A2:BTN2A1:HMGN4:ABT1:ZNF322:PRSS16:ZNF184:OR2B2:OR2B6:ZSCAN31:ZSCAN23:SCAND3:TRIM27:ZNF311:OR2J3:OR2J2:OR14J1:UBD:GABBR1:AK9:EPHX2:CLU* |
| **Blood protein levels** | **1553** | **172** | **2.94E-13** | **3.81E-11** | ***TESK2:CCDC163P:MMACHC:PRDX1:AKR1A1:NASP:CCDC17:GPBP1L1:TMEM69:IPP:MAST2:MCL1:GOLPH3L:HORMAD1:CTSS:CTSK:FCGR2A:HSPA6:FCGR3A:C4BPA:CD55:NRP1:RET:CSGALNACT2:ARHGAP1:PTPRJ:FOLH1:GLYAT:BRAP:TMEM120B:CDK2AP1:THSD1:GNPNAT1:FLRT2:PDIA3:DAPK2:MAPK3:ZDHHC1:HSD11B2:ATP6V0D1:AGRP:FAM65A:TXNL4B:DHX38:PMFBP1:CYB5D2:GP1BA:PYY:ACE:ACE:HMHA1:BCAM:PVRL2:APOE:APOC1:APOC4-APOC2:APOC4:APOC2:CKM:CD3EAP:QPCTL:MDH1:BIN1:PLA2R1:NCKIPSD:IP6K2:PRKAR2A:SLC25A20:ARIH2OS:ARIH2:P4HTM:WDR6:DALRD3:NDUFAF3:IMPDH2:QRICH1:QARS:USP19:LAMB2:CCDC71:KLHDC8B:C3orf84:CCDC36:USP4:BSN:APEH:MST1:RNF123:AMIGO3:GMPPB:IP6K1:TRAIP:CAMKV:RBM6:RBM5:C3orf18:MAPKAPK3:DOCK3:MANF:ERC2:PROK2:LSAMP:GHR:SEPP1:CD14:NDUFA2:SLC17A3:BTN3A3:BTN2A1:BTN1A1:HMGN4:ABT1:ZNF322:HIST1H2BL:HIST1H2AI:HIST1H3H:HIST1H2AJ:HIST1H2BM:HIST1H4J:HIST1H4K:HIST1H2AK:HIST1H2BN:HIST1H2AL:HIST1H1B:HIST1H3I:HIST1H4L:HIST1H2AM:HIST1H2BO:OR2B2:ZNF165:ZSCAN16:ZKSCAN8:ZSCAN9:NKAPL:PGBD1:ZSCAN31:ZKSCAN3:ZSCAN12:ZSCAN23:GPX6:SCAND3:TRIM27:C6orf100:ZNF311:OR2W1:OR2B3:OR2J3:OR2J2:OR14J1:OR5V1:OR12D3:OR11A1:OR10C1:OR2H1:MAS1L:UBD:GABBR1:TREML2:TREM1:LRP11:KDELR2:TPST1:CYP3A7:PILRB:PILRA:LRCH4:PCOLCE:EPO:ZYX:EPHA1:CNTNAP2:SCARA5:CD72*** |
| Regular attendance at a gym or sports club | 38 | 18 | 2.79E-12 | 3.37E-10 | *BSN:APEH:MST1:RNF123:AMIGO3:GMPPB:IP6K1:CDHR4:FAM212A:UBA7:TRAIP:CAMKV:MST1R:MON1A:RBM6:RBM5:SEMA3F:CADM2* |
| Body fat distribution (arm fat ratio) | 129 | 33 | 3.77E-12 | 4.27E-10 | *SLC39A13:PSMC3:RAPSN:CELF1:NDUFS3:PTPMT1:KBTBD4:KBTBD4:C1QTNF4:LIMA1:LARP4:TMEM219:TAOK2:HIRIP3:INO80E:DOC2A:FAM57B:ALDOA:PPP4C:TBX6:YPEL3:GDPD3:ZNF668:ZNF668:ZNF646:PRSS53:VKORC1:NFAT5:NOB1:WWP2:RBM6:DOCK3:C6orf106:SNRPC:UHRF1BP1* |
| **Body mass index (age>50)** | **59** | **22** | **4.05E-12** | **4.33E-10** | ***ZNF229:ZNF180:IGSF23:PVR:BCL3:CBLC:BCAM:PVRL2:TOMM40:APOE:APOC1:CLPTM1:RELB:CLASRP:GEMIN7:MARK4:PPP1R37:BLOC1S3:EXOC3L2:CKM:KLC3:ERCC1*** |
| Parkinson's disease | 161 | 36 | 2.79E-11 | 2.81E-09 | *ITPKB:FAM171A1:DLG2:SYT10:C12orf40:SLC2A13:LRRK2:CCDC62:HIP1R:OGFOD2:STX1B:ZNF646:PRSS53:BCKDK:KAT8:RAI1:SREBF1:ARHGAP27:PLEKHM1:CRHR1:SPPL2C:MAPT:STH:KANSL1:NSF:WNT3:CYP1B1:TMEM163:ACMSD:CCNT2:NCKIPSD:MRPS30:ELOVL7:NDUFAF2:ZNF184:HIST1H2BL* |
| Intelligence (MTAG) | 246 | 46 | 3.90E-11 | 3.73E-09 | *COL11A1:CACNA1E:C12orf76:IFT81:PITPNM2:MPHOSPH9:CDK2AP1:EFTUD1:NSF:WNT3:CKM:BMPR2:CCDC134:NCKIPSD:USP4:BSN:IP6K1:TRAIP:CAMKV:MON1A:RBM6:SEMA3F:CACNA2D2:CADM2:FNIP2:ELOVL7:NDUFAF2:ZSWIM6:CYSTM1:HBEGF:SLC17A4:SLC17A1:HFE:HIST1H2AC:HIST1H1E:HIST1H2BD:HIST1H2BF:BTN2A2:BTN3A1:BTN2A1:BTN1A1:ABT1:ZNF322:POM121L2:HIST1H2BN:AVL9* |
| Lung cancer | 157 | 35 | 5.74E-11 | 5.20E-09 | *GIPC2:CATSPER2:WDR76:MLTK:MTMR3:ARAP3:SLC17A4:TRIM38:HIST1H1A:BTN3A1:ABT1:ZNF322:PRSS16:ZNF184:HIST1H2BL:HIST1H2BO:OR2B2:PGBD1:ZSCAN31:ZSCAN12:ZSCAN23:SCAND3:C6orf100:ZNF311:OR2J2:OR14J1:UBD:GABBR1:RIMS1:CD164:RFX6:GINM1:KATNA1:CHRNA2:EPHX2* |
| General cognitive ability | 185 | 37 | 4.46E-10 | 3.86E-08 | *MTCH2:SPPL2A:EFTUD1:CPEB1:RAI1:ARHGAP27:MAPT:WNT3:KCNJ3:TANK:DESI1:SREBF2:SEPT3:IP6K2:BSN:IP6K1:CDHR4:CAMKV:RBM6:SEMA3F:GNAT1:CADM2:ZSWIM6:HBEGF:SLC17A1:HIST1H1E:HIST1H2BD:BTN3A2:BTN2A2:BTN3A1:BTN2A1:HMGN4:ABT1:POM121L2:HIST1H2BN:OR5V1:AUTS2* |
| Loneliness (MTAG) | 67 | 21 | 5.37E-10 | 4.38E-08 | *MADD:MYBPC3:SPI1:SLC39A13:PSMC3:RAPSN:CELF1:NDUFS3:PTPMT1:KBTBD4:KBTBD4:FAM180B:C1QTNF4:MTCH2:AGBL2:FNBP4:NUP160:OR5B3:OR5B2:OR5B12:OR5B21:ETFA* |
| Alcohol use disorder (total score) | 29 | 14 | 5.55E-10 | 4.38E-08 | *SPI1:SLC39A13:PSMC3:RAPSN:CELF1:NDUFS3:PLEKHM1:CRHR1:SPPL2C:MAPT:STH:KANSL1:NSF:WNT3* |
| **Alzheimer's disease** | **63** | **20** | **1.07E-09** | **8.11E-08** | ***CR1:MS4A3:MS4A6A:MS4A4E:PICALM:ABCA7:ZNF224:PVR:BCL3:BCAM:PVRL2:TOMM40:APOE:APOC1:EXOC3L2:BIN1:PFDN1:HBEGF:CNTNAP2:CLU*** |
| Alzheimer's disease in APOE e4- carriers | 36 | 15 | 1.74E-09 | 1.27E-07 | *MS4A6A:MS4A4A:MS4A6E:PICALM:SLC24A4:CRHR1:MAPT:KANSL1:WNT3:BIN1:PFDN1:HBEGF:EPHA1:PTK2B:CLU* |
| Extremely high intelligence | 81 | 22 | 4.32E-09 | 3.02E-07 | *COL11A1:TANK:BSN:APEH:MST1:RNF123:AMIGO3:GMPPB:IP6K1:CDHR4:FAM212A:UBA7:TRAIP:CAMKV:MST1R:MON1A:RBM6:RBM5:SEMA3F:GNAT1:LSM5:AVL9* |
| Squamous cell lung carcinoma | 89 | 23 | 5.53E-09 | 3.72E-07 | *CATSPER2:HCN1:SCGN:HIST1H2AC:HIST1H1E:BTN3A2:BTN3A3:ZNF322:PRSS16:ZNF184:OR2B2:OR2B6:ZNF165:PGBD1:ZSCAN12:GPX6:SCAND3:C6orf100:ZNF311:OR2J3:OR2J2:OR14J1:RIMS1* |
| **Cerebrospinal AB1-42 levels in mild cognitive impairment** | **16** | **10** | **6.19E-09** | **3.88E-07** | ***BCAM:PVRL2:TOMM40:APOE:APOC1:APOC4-APOC2:APOC4:APOC2:CLPTM1:RELB*** |
| **Cerebrospinal fluid t-tau levels in mild cognitive impairment** | **16** | **10** | **6.19E-09** | **3.88E-07** | ***BCAM:PVRL2:TOMM40:APOE:APOC1:APOC4-APOC2:APOC4:APOC2:CLPTM1:RELB*** |
| Tourette syndrome | 102 | 24 | 1.92E-08 | 1.16E-06 | *PICALM:CCDC62:HIP1R:VPS37B:ABCB9:OGFOD2:ARL6IP4:PITPNM2:MPHOSPH9:C12orf65:CDK2AP1:SBNO1:SETD8:RILPL2:EFTUD1:CPEB1:AP3B2:MARCH7:CD302:LY75:LY75-CD302:PLA2R1:TANK:ERC2* |
| Mood instability | 61 | 18 | 2.66E-08 | 1.55E-06 | *LRP4:NR1H3:RAPSN:NDUFS3:PTPMT1:KBTBD4:KBTBD4:MTCH2:CALB2:CRHR1:MAPT:WNT3:RANGAP1:NDUFAF3:QARS:LAMB2:CCDC36:BSN:AMIGO3* |
| Body fat distribution (leg fat ratio) | 223 | 38 | 2.73E-08 | 1.55E-06 | *SLC39A13:PSMC3:RAPSN:CELF1:NDUFS3:PTPMT1:KBTBD4:KBTBD4:C1QTNF4:LIMA1:LARP4:PITPNM2:NFAT5:NOB1:WWP2:ARIH2OS:ARIH2:P4HTM:WDR6:DALRD3:NDUFAF3:IMPDH2:QRICH1:QARS:RBM6:HIST1H2BD:HIST1H2BE:HIST1H4D:HIST1H3D:HIST1H2AD:HIST1H2BF:HIST1H4E:HIST1H3E:HIST1H1D:HIST1H3G:HIST1H2BI:C6orf106:SNRPC:UHRF1BP1* |
| **Cerebrospinal AB1-42 levels in Alzheimer's disease dementia** | **18** | **10** | **3.01E-08** | **1.65E-06** | ***CBLC:BCAM:PVRL2:TOMM40:APOE:APOC1:APOC4-APOC2:APOC4:APOC2:CLPTM1*** |
| **Cerebrospinal fluid AB1-42 levels** | **50** | **16** | **4.39E-08** | **2.29E-06** | ***RNF111:BCAM:PVRL2:TOMM40:APOE:APOC1:APOC4-APOC2:APOC4:APOC2:CLPTM1:RELB:GEMIN7:PPP1R37:NKPD1:TRAPPC6A:GPR141*** |
| **Body mass index x sex x age interaction (4df test)** | **91** | **22** | **4.41E-08** | **2.29E-06** | ***ZNF229:ZNF180:IGSF23:PVR:BCL3:CBLC:BCAM:PVRL2:TOMM40:APOE:APOC1:CLPTM1:RELB:CLASRP:GEMIN7:MARK4:PPP1R37:BLOC1S3:EXOC3L2:CKM:KLC3:ERCC1*** |
| Reaction time | 39 | 14 | 5.90E-08 | 2.92E-06 | *DGKZ:ATG13:ARHGAP1:LRP4:HECTD4:PTPN11:MAPT:KANSL1:ARL17B:LRRC37A2:ARL17A:NSF:WNT3:PFDN1* |
| **Logical memory (immediate recall)** | **19** | **10** | **5.98E-08** | **2.92E-06** | ***BCAM:PVRL2:TOMM40:APOE:APOC1:APOC4-APOC2:APOC4:APOC2:CLPTM1:RELB*** |
| Chronic obstructive pulmonary disease or coronary artery disease (pleiotropy) | 15 | 9 | 6.12E-08 | 2.92E-06 | *SNRPD2:QPCTL:FBXO46:SIX5:DMPK:DMWD:RSPH6A:SYMPK:FOXA3* |
| Coffee consumption | 95 | 22 | 1.01E-07 | 4.69E-06 | *PABPC4:SETDB1:BRAP:ACAD10:ALDH2:MAPKAPK5:NAA25:TRAFD1:HECTD4:RPL6:CPLX3:ULK3:HYDIN:SREBF1:EHBP1:SLC25A17:CCDC36:CACNA2D2:FNIP2:EYS:AUTS2:POR* |
| Morning person | 199 | 34 | 1.36E-07 | 6.18E-06 | *HP1BP3:EIF4G3:ECE1:ENSA:GOLPH3L:OR5B12:OR5B21:ZFP91:SYT10:ALG10:ALG10B:CPNE8:KIF21A:DHODH:PMFBP1:ZFHX3:RASD1:FSHR:NRXN1:IP6K2:PRKAR2A:ARIH2:QARS:BSN:RNF123:CAMKV:RBM6:RBM5:SEMA3F:GNAI2:CACNA2D2:DOCK3:GRIA1:LSM5* |
| **Cerebrospinal fluid p-tau levels in mild cognitive impairment** | **21** | **10** | **2.03E-07** | **8.77E-06** | ***BCAM:PVRL2:TOMM40:APOE:APOC1:APOC4:APOC2:CLPTM1:RELB:FAM220A*** |
| **Logical memory (delayed recall)** | **21** | **10** | **2.03E-07** | **8.77E-06** | ***BCAM:PVRL2:TOMM40:APOE:APOC1:APOC4-APOC2:APOC4:APOC2:CLPTM1:RELB*** |
| Loneliness | 91 | 21 | 2.09E-07 | 8.81E-06 | *MADD:MYBPC3:SPI1:SLC39A13:PSMC3:RAPSN:CELF1:NDUFS3:PTPMT1:KBTBD4:KBTBD4:FAM180B:C1QTNF4:MTCH2:AGBL2:FNBP4:NUP160:OR5B3:OR5B2:OR5B12:OR5B21:ETFA* |
| Regular attendance at a religious group | 70 | 18 | 2.71E-07 | 1.12E-05 | *EHBP1:OTX1:WDPCP:BSN:APEH:MST1:RNF123:AMIGO3:GMPPB:IP6K1:CDHR4:FAM212A:UBA7:TRAIP:CAMKV:MST1R:MON1A:RBM6* |
| Handedness (Right-handed vs. non-right-handed) | 10 | 7 | 4.35E-07 | 1.75E-05 | *STH:KANSL1:ARL17B:LRRC37A:LRRC37A2:ARL17A:NSF* |
| **Hippocampal volume in Alzheimer's disease dementia** | **52** | **15** | **5.32E-07** | **2.10E-05** | ***COG4:SF3B3:IL34:MTSS1L:VAC14:BCAM:PVRL2:TOMM40:APOE:APOC1:APOC4-APOC2:APOC4:APOC2:CLPTM1:RELB*** |
| **Cerebrospinal fluid t-tau levels** | **23** | **10** | **5.85E-07** | **2.26E-05** | ***BCAM:PVRL2:TOMM40:APOE:APOC1:APOC4-APOC2:APOC4:APOC2:CLPTM1:RELB*** |
| **HDL cholesterol** | **233** | **36** | **7.56E-07** | **2.86E-05** | ***MACF1:PABPC4:LRP4:ACP2:NR1H3:MADD:FOLH1:OR4C46:MYO1H:KCTD10:UBE3B:MMAB:MVK:HECTD4:SBNO1:TUBGCP4:CATSPER2:LIPC:ETFA:CTCF:GFOD2:RANBP10:SPC24:PVRL2:TOMM40:APOE:APOC1:APOC2:IP6K2:RBM6:RBM5:MSL2:STAG1:C6orf106:SNRPC:DAGLB*** |
| Body fat distribution (trunk fat ratio) | 236 | 36 | 1.03E-06 | 3.83E-05 | *SLC39A13:PSMC3:RAPSN:CELF1:NDUFS3:PTPMT1:KBTBD4:KBTBD4:C1QTNF4:LIMA1:LARP4:PITPNM2:ARIH2OS:ARIH2:P4HTM:WDR6:DALRD3:NDUFAF3:IMPDH2:QRICH1:QARS:RBM6:DOCK3:HIST1H2BD:HIST1H2BE:HIST1H4D:HIST1H3D:HIST1H2AD:HIST1H2BF:HIST1H4E:HIST1H3E:HIST1H1D:HIST1H3G:HIST1H2BI:C6orf106:SNRPC:UHRF1BP1* |
| Handedness (Left-handed vs. non-left-handed) | 11 | 7 | 1.13E-06 | 4.10E-05 | *STH:KANSL1:ARL17B:LRRC37A:LRRC37A2:ARL17A:NSF* |
| General factor of neuroticism | 78 | 18 | 1.52E-06 | 5.42E-05 | *ARFGAP2:MADD:RAPSN:AGBL2:MYO1H:ETFA:ARHGAP27:CRHR1:MAPT:KANSL1:NSF:WNT3:WDPCP:ZC3H7B:QRICH1:CADM2:LSAMP:PPP2R3A* |
| Functional impairment in major depressive disorder, bipolar disorder and schizophrenia | 8 | 6 | 1.69E-06 | 5.79E-05 | *FCGR2A:HSPA6:FCGR3A:ZZEF1:CYB5D2:ANKFY1* |
| Ticagrelor levels in individuals with acute coronary syndromes treated with ticagrelor | 8 | 6 | 1.69E-06 | 5.79E-05 | *ARPC1A:CYP3A43:ZKSCAN1:STAG3:GATS:PVRIG* |
| Ulcerative colitis | 362 | 47 | 2.92E-06 | 9.82E-05 | *FCGR2A:HSPA6:FCGR3A:CCNY:OR5B2:OR5B12:OR5B21:LPXN:ZFP91:ZFP91-CNTF:CNTF:GLYAT:PEMT:NCKIPSD:IP6K2:PRKAR2A:SLC25A20:ARIH2OS:ARIH2:P4HTM:WDR6:DALRD3:NDUFAF3:IMPDH2:QRICH1:QARS:USP19:LAMB2:CCDC71:KLHDC8B:C3orf84:CCDC36:C3orf62:USP4:BSN:APEH:MST1:RNF123:AMIGO3:GMPPB:IP6K1:UBA7:MST1R:DAGLB:KDELR2:KPNA7:CNTNAP2* |
| Bipolar I disorder | 82 | 18 | 3.29E-06 | 0.000108446 | *VKORC1:BCKDK:KAT8:PRSS8:PRSS36:FUS:MCHR1:SLC25A17:ST13:XPNPEP3:DNAJB7:RBX1:CADM2:PFDN1:HBEGF:SLC4A9:ANKHD1:ANKHD1-EIF4EBP3* |
| **Cerebrospinal fluid p-tau levels** | **17** | **8** | **3.95E-06** | **0.000125664** | ***BCAM:PVRL2:APOE:APOC1:APOC4:APOC2:CLPTM1:RELB*** |
| Alcohol consumption (max-drinks) | 17 | 8 | 3.95E-06 | 0.000125664 | *BRAP:ACAD10:ALDH2:NAA25:TRAFD1:HECTD4:PTPN11:RPH3A* |
| Intraocular pressure | 346 | 45 | 4.51E-06 | 0.000141087 | *COL24A1:F2:LRP4:C11orf49:NR1H3:MYBPC3:SPI1:PSMC3:RAPSN:AGBL2:NUP160:PTPRJ:OR4C5:OR4A47:TRIM49B:FOLH1:OR4C13:OR4A5:OR4C46:TRIM48:OR4A15:OR4C15:OR4P4:OR4S2:OR8H3:OR5J2:OR5T2:OR5AP2:OR5AR1:ME3:FAM186A:IL34:CAMTA2:INCA1:NSF:WNT3:SPC24:SEMA3F:CADM2:PPP2R3A:STAG1:PTCD2:ZNF366:PCDHGA5:VKORC1L1* |
| Experiencing mood swings | 40 | 12 | 4.63E-06 | 0.000142387 | *ARHGAP1:MYBPC3:SPI1:PTPRJ:ORAI1:MAPT:XPNPEP3:PHF5A:IP6K2:LSAMP:TRIM27:EYS* |
| **Alzheimer's disease in hypertension** | **9** | **6** | **4.80E-06** | **0.000145135** | ***SLC2A13:BCAM:PVRL2:TOMM40:APOE:APOC1*** |
| Age at first birth | 28 | 10 | 4.99E-06 | 0.000146212 | *RNF123:CAMKV:MST1R:MON1A:RBM6:RBM5:SEMA3F:HYAL3:MRPS30:HCN1* |
| Social communication problems | 28 | 10 | 4.99E-06 | 0.000146212 | *RNF111:RAB8B:TRIM27:ZNF311:OR2W1:OR2B3:OR2J3:OR2J2:OR5V1:OR12D3* |
| Response to alcohol consumption (flushing response) | 13 | 7 | 5.24E-06 | 0.000150927 | *BRAP:ACAD10:ALDH2:NAA25:TRAFD1:HECTD4:PTPN11* |
| Global electrical heterogeneity phenotypes | 18 | 8 | 6.70E-06 | 0.000190149 | *SPI1:SLC39A13:PSMC3:RAPSN:CELF1:AGBL2:FNBP4:NUP160* |
| Neurociticism | 95 | 19 | 7.58E-06 | 0.000211736 | *ARHGAP1:C11orf49:DDB2:ACP2:NR1H3:SCAPER:PRR14:STH:OTX1:ZNF804A:MCHR1:SLC25A17:SLC35G2:GALNT10:HIST1H1C:BTN2A2:HIST1H2BK:OR2B6:TRIM27* |
| Empathy quotient | 14 | 7 | 9.90E-06 | 0.000268124 | *ZDHHC1:HSD11B2:ATP6V0D1:ENKD1:GFOD2:RANBP10:TSNAXIP1* |
| Caffeine consumption | 14 | 7 | 9.90E-06 | 0.000268124 | *LMAN1L:CPLX3:ULK3:SCAMP2:COX5A:SCAMP5:PPCDC* |
| **Low density lipoprotein cholesterol levels** | **43** | **12** | **1.06E-05** | **0.000283647** | ***RPH3A:LIPC:DHODH:TXNL4B:PMFBP1:LDLR:SPC24:PVRL2:APOE:APOC1:APOC2:FSHR*** |
| HDL cholesterol levels in current drinkers | 106 | 20 | 1.11E-05 | 0.00029105 | *PABPC4:HEYL:MYBPC3:FOLH1:OR4A5:OR4C6:OR5D13:UBE3B:SBNO1:TP53BP1:LIPC:CEACAM16:BCL3:RBM5:MSL2:PCCB:HIST1H3A:HIST1H4A:C6orf106:DAGLB* |
| Neutrophil count | 154 | 25 | 1.51E-05 | 0.000390545 | *MAST2:FAM186A:MVK:HECTD4:SPPL2A:CRHR1:GRIN3B:ABCA7:MARK4:LY75:LY75-CD302:PCDHA1:PCDHA2:PCDHA3:PCDHA4:PCDHA5:PCDHA6:PCDHA7:PCDHA8:PCDHA9:PCDHA10:PCDHA11:PCDHA12:PCDHA13:PSD3* |
| Rhegmatogenous retinal detachment | 20 | 8 | 1.72E-05 | 0.000439663 | *ADAMTSL4:MCL1:ENSA:GOLPH3L:HORMAD1:CTSS:CTSK:PRUNE* |
| Crohn's disease | 597 | 65 | 1.80E-05 | 0.000454362 | *CUL2:CREM:CCNY:IPMK:CISD1:PRG3:PRG2:SLC2A13:LRRK2:MUC19:BRAP:ACAD10:ALDH2:MAPKAPK5:TMEM116:ERP29:NAA25:SLC52A1:HMHA1:NANOS2:EHBP1:MARCH7:LY75:PLA2R1:INPP5D:ASCC2:MTMR3:RBX1:EP300:L3MBTL2:CHADL:TEF:PMM1:NHP2L1:NCKIPSD:IP6K2:PRKAR2A:SLC25A20:ARIH2OS:ARIH2:P4HTM:WDR6:DALRD3:NDUFAF3:IMPDH2:QRICH1:QARS:USP19:LAMB2:CCDC71:KLHDC8B:C3orf84:CCDC36:C3orf62:USP4:BSN:APEH:MST1:RNF123:HFE:UBD:GABBR1:CNTNAP2:PTK2B:EPHX2* |
| **Resting heart rate** | **138** | **23** | **2.11E-05** | **0.000525397** | ***CD34:SYT10:ALG10B:CPNE8:FLRT2:SREBF1:PVRL2:APOE:APOC1:PPP1R13L:CALCRL:PRKAR2A:QRICH1:LAMB2:KLHDC8B:GMPPB:IP6K1:FAM212A:PCOLCE:GIGYF1:EPHB4:SLC12A9:SRRT*** |
| Iron status biomarkers | 21 | 8 | 2.62E-05 | 0.000643281 | *GHR:SCGN:SLC17A1:TRIM38:HFE:BTN1A1:HIST1H2BJ:TFR2* |
| Sum basophil neutrophil counts | 116 | 20 | 4.35E-05 | 0.001051841 | *MAST2:FAM186A:HECTD4:SPPL2A:CRHR1:ABCA7:MARK4:PCDHA1:PCDHA2:PCDHA3:PCDHA4:PCDHA5:PCDHA6:PCDHA7:PCDHA8:PCDHA9:PCDHA10:PCDHA11:PCDHA12:PCDHA13* |
| Immunoglobulin light chain (AL) amyloidosis | 12 | 6 | 4.46E-05 | 0.001066016 | *PRR14:FBRS:SRCAP:RNF40:BCL7C:ULK4* |
| HDL cholesterol levels x alcohol consumption (regular vs non-regular drinkers) interaction (2df) | 118 | 20 | 5.59E-05 | 0.001317191 | *PABPC4:HEYL:MYBPC3:FOLH1:OR4A5:OR4C6:OR5D13:UBE3B:SBNO1:TP53BP1:LIPC:CEACAM16:BCL3:RBM5:MSL2:PCCB:HIST1H3A:HIST1H4A:C6orf106:DAGLB* |
| HDL cholesterol levels x alcohol consumption (drinkers vs non-drinkers) interaction (2df) | 128 | 21 | 6.03E-05 | 0.001403043 | *PABPC4:HEYL:MYBPC3:FOLH1:OR4A5:OR4C6:OR5D13:UBE3B:SBNO1:TP53BP1:LIPC:LDLR:CEACAM16:BCL3:RBM5:MSL2:PCCB:HIST1H3A:HIST1H4A:C6orf106:DAGLB* |
| Inflammatory bowel disease | 637 | 66 | 7.21E-05 | 0.0016554 | *FCGR2A:HSPA6:FCGR3A:CUL2:CREM:CCNY:IPMK:CISD1:LPXN:CNTF:SLC2A13:LRRK2:MUC19:BRAP:ACAD10:ALDH2:MAPKAPK5:TMEM116:ERP29:NAA25:MARCH7:LY75:PLA2R1:ASCC2:MTMR3:L3MBTL2:CHADL:TEF:PMM1:NHP2L1:NCKIPSD:IP6K2:PRKAR2A:SLC25A20:ARIH2OS:ARIH2:P4HTM:WDR6:DALRD3:NDUFAF3:IMPDH2:QRICH1:QARS:USP19:LAMB2:CCDC71:KLHDC8B:C3orf84:CCDC36:C3orf62:USP4:BSN:APEH:MST1:RNF123:IP6K1:MST1R:SEPP1:RFX6:DAGLB:KDELR2:KPNA7:EPO:CNTNAP2:PTK2B:EPHX2* |
| Takayasu arteritis | 52 | 12 | 8.37E-05 | 0.001899897 | *FCGR2A:FCGR3A:MSH6:ROBO1:HIST1H3G:GPX6:GPX5:OR2B3:OR2J3:OR2J2:OR14J1:PTK2B* |
| Immunoglobulin A | 19 | 7 | 0.00010941 | 0.002421644 | *DGKZ:MDK:AMBRA1:HARBI1:ATG13:F2:CKAP5* |
| Alcohol dependence symptom count | 19 | 7 | 0.00010941 | 0.002421644 | *BRAP:ACAD10:ALDH2:NAA25:TRAFD1:HECTD4:PTPN11* |
| **Quantitative traits** | **25** | **8** | **0.00011076** | **0.002422083** | ***TOMM40:APOE:APOC1:APOC4:APOC2:BLOC1S3:EXOC3L2:SDK1*** |
| Cholangiocarcinoma in primary sclerosing cholangitis (time to event) | 14 | 6 | 0.00012979 | 0.002804406 | *CACNA2D2:C3orf18:HEMK1:CISH:MAPKAPK3:DOCK3* |
| Feeling miserable | 33 | 9 | 0.00016497 | 0.003522496 | *MDK:ARHGAP1:DDB2:ACP2:NR1H3:KCNJ3:MST1:LSAMP:TRIM27* |
| **Alzheimer's disease in hypertension-negative individuals** | **10** | **5** | **0.0002037** | **0.004299012** | ***BCAM:PVRL2:TOMM40:APOE:APOC1*** |
| Iron status biomarkers (total iron binding capacity) | 35 | 9 | 0.00026899 | 0.005611608 | *CCDC17:GPBP1L1:IPP:MAST2:SCGN:SLC17A2:HFE:HIST1H2BJ:PRSS16* |
| Esophageal cancer | 16 | 6 | 0.00030974 | 0.006388385 | *BRAP:ACAD10:ALDH2:HECTD4:RPL6:UNC5CL* |
| Facial emotion recognition (sad faces) | 23 | 7 | 0.00042463 | 0.008659593 | *CHN2:KPNA7:ARPC1A:ARPC1B:PDAP1:ZNF789:ZNF394* |
| Feeling fed-up | 30 | 8 | 0.00045014 | 0.008934751 | *HARBI1:FOLH1:OR4A5:TRIM48:FAM117B:IP6K2:CADM2:HCN1* |
| Hepcidin/ferritin ratio | 30 | 8 | 0.00045014 | 0.008934751 | *TACC2:GLYAT:STON1:GTF2A1L:PRELID2:SLC17A3:HFE:ZNF165* |
| Sense of smell | 17 | 6 | 0.00045289 | 0.008934751 | *KANSL1:LRRC37A:LRRC37A2:NSF:TOMM40:ERC2* |
| Laterality in neovascular age-related macular degeneration | 7 | 4 | 0.00049797 | 0.009718496 | *STON1:GTF2A1L:LHCGR:FSHR* |
| Alzheimer's disease in APOE e4+ carriers | 13 | 5 | 0.00088472 | 0.016633286 | *CR1:PICALM:BIN1:CADM2:CLU* |
| **Lipoprotein phospholipase A2 activity in cardiovascular disease** | **13** | **5** | **0.00088472** | **0.016633286** | ***TP53BP1:MFAP1:FRMD5:LDLR:APOE*** |
| Feeling tense | 19 | 6 | 0.00088894 | 0.016633286 | *C16orf93:PHF5A:WBP2NL:APEH:CADM2:GALNT10* |
| Heart rate increase in response to exercise | 19 | 6 | 0.00088894 | 0.016633286 | *SYT10:ALG10:ALG10B:C7orf43:TRIP6:SRRT* |
| Headache | 27 | 7 | 0.0012282 | 0.022746689 | *MACF1:ATG13:MYO1H:IFT81:CRHR1:MAPT:BTN2A2* |
| Hematocrit | 128 | 18 | 0.0013125 | 0.024062502 | *NASP:CCDC17:FCGR2A:RANBP10:HYDIN:CRHR1:WNT3:MARK4:TMEM163:ACMSD:HFE:HIST1H1T:NYAP1:AGFG2:TFR2:ACTL6B:GNB2:EPO* |
| Educational attainment | 71 | 12 | 0.00169222 | 0.030713754 | *PITPNM2:MPHOSPH9:C12orf65:SBNO1:SETD8:RILPL2:PPP1R21:NRXN1:TANK:CAMKV:CADM2:FNIP2* |
| Chronic obstructive pulmonary disease or high blood pressure (pleiotropy) | 72 | 12 | 0.00191487 | 0.034295911 | *MACF1:BMP8A:PABPC4:TMEM219:TAOK2:HIRIP3:INO80E:DOC2A:C16orf92:FAM57B:ALDOA:PPP4C* |
| **HDL cholesterol levels** | **143** | **19** | **0.00192737** | **0.034295911** | ***PABPC4:HEYL:LRP4:MYBPC3:OR4C46:OR4C6:OR5D13:UBE3B:MVK:HECTD4:SBNO1:TP53BP1:LIPC:APOE:APOC1:APOC2:RBM5:C6orf106:DAGLB*** |
| Alcohol consumption | 22 | 6 | 0.00207215 | 0.036514164 | *CCDC63:ALDH2:HECTD4:TANK:CADM2:AUTS2* |
| **LDL cholesterol** | **178** | **22** | **0.00227851** | **0.039764441** | ***CR1L:CREB3L1:BRAP:SCAMP5:DHX38:ZFHX3:LDLR:CBLC:BCAM:TOMM40:APOE:APOC1:APOC4:APOC2:EHBP1:FAM117B:MTMR3:SLC17A2:HFE:HIST1H4C:C6orf106:GIGYF1*** |
| Suffering from nerves | 5 | 3 | 0.00236521 | 0.040884429 | *ARHGAP1:ACP2:CADM2* |
| **Hippocampal volume** | **47** | **9** | **0.00260099** | **0.044535868** | ***BCAM:PVRL2:TOMM40:APOE:APOC1:APOC4:APOC2:CLPTM1:RELB*** |
| Heart rate response to recovery post exercise (10 sec) | 23 | 6 | 0.00265363 | 0.044595769 | *SYT10:ALG10:ALG10B:C7orf43:TRIP6:SRRT* |
| Heart rate response to recovery post exercise (30 sec) | 23 | 6 | 0.00265363 | 0.044595769 | *SYT10:ALG10:ALG10B:C7orf43:TRIP6:SRRT* |

Traits reported in FUMA Analysis, in GWAS catalog category with genes in common with the genes found in PRS extremes GWAS.

**Table S5-A. PheWAS results, Beta >= 0.1 using PRS calculated from Jansen et *al*. including *APOE* locus.**

| **n** | **Beta** | **Lower CI 95** | **Upper CI 95** | ***p*-value** | **Trait/Description** | **Category** |
| --- | --- | --- | --- | --- | --- | --- |
| **27385/10399(37784)** | **4.41** | **4.29** | **4.54** | **0** | **Illnesses of mother: Alzheimer's disease/dementia** | **Family history** |
| **32263/5521(37784)** | **3.96** | **3.79** | **4.13** | **0** | **Illnesses of father: Alzheimer's disease/dementia** | **Family history** |
| **37473/311(37784)** | **3.22** | **2.69** | **3.86** | **9.70E-28** | **G30.9 Alzheimer's disease, unspecified** | **Summary Diagnoses** |
| **37581/203(37784)** | **2.00** | **1.59** | **2.45** | **5.72E-20** | **F03 Unspecified dementia** | **Summary Diagnoses** |
| **37468/316(37784)** | **1.21** | **0.94** | **1.48** | **9.85E-19** | **Illnesses of siblings: Alzheimer's disease/dementia** | **Family history** |
| **37490/292(37782)** | **0.58** | **0.34** | **0.83** | **2.24E-06** | **Treatment/medication code: ginkgo forte tablet** | **Medications** |
| **37546/238(37784)** | **0.58** | **0.31** | **0.85** | **2.51E-05** | **R15 Faecal incontinence** | **Summary Diagnoses** |
| **37459/325(37784)** | **0.55** | **0.32** | **0.78** | **3.07E-06** | **R41.0 Disorientation, unspecified** | **Summary Diagnoses** |
| **37422/362(37784)** | **0.54** | **0.32** | **0.76** | **1.12E-06** | **R29.6 Tendency to fall, not elsewhere classified** | **Summary Diagnoses** |
| **37070/714(37784)** | **0.51** | **0.36** | **0.66** | **9.61E-11** | **Illnesses of mother: Parkinson's disease** | **Family history** |
| **36631/1153(37784)** | **0.46** | **0.33** | **0.58** | **1.36E-13** | **Illnesses of father: Parkinson's disease** | **Family history** |
| 37365/417(37782) | 0.41 | 0.21 | 0.61 | 5.59E-05 | Treatment/medication code: tramadol | Medications |
| 36382/1402(37784) | 0.35 | 0.24 | 0.46 | 2.04E-10 | Illnesses of father: Severe depression | Family history |
| 36604/1178(37782) | 0.35 | 0.23 | 0.47 | 8.45E-09 | Treatment/medication code: atorvastatin | Medications |
| 37122/662(37784) | 0.34 | 0.19 | 0.50 | 2.05E-05 | I25.8 Other forms of chronic ischaemic heart disease | Summary Diagnoses |
| 36610/1172(37782) | 0.34 | 0.22 | 0.46 | 3.13E-08 | Non-cancer illness code, self-reported: angina | Medical conditions |
| 36498/1206(37704) | 0.33 | 0.22 | 0.45 | 3.55E-08 | Vascular/heart problems diagnosed by doctor: Angina | Health and medical history |
| 4634/933(5567) | 0.31 | 0.17 | 0.46 | 3.13E-05 | Chest pain or discomfort walking normally | Health and medical history |
| 34918/2776(37694) | 0.30 | 0.21 | 0.38 | 2.70E-12 | Medication for cholesterol, blood pressure, diabetes, or take exogenous hormones: Cholesterol lowering medication | Health and medical history |
| 36451/1333(37784) | 0.29 | 0.18 | 0.40 | 2.79E-07 | F32.9 Depressive episode, unspecified | Summary Diagnoses |
| 36340/1444(37784) | 0.29 | 0.18 | 0.40 | 1.36E-07 | N39.0 Urinary tract infection, site not specified | Summary Diagnoses |
| 36145/1639(37784) | 0.27 | 0.17 | 0.37 | 1.15E-07 | U05.1 Computed tomography of head | Summary Operations |
| 36876/906(37782) | 0.27 | 0.14 | 0.41 | 8.26E-05 | Treatment/medication code: co-codamol | Medications |
| 35291/2493(37784) | 0.27 | 0.19 | 0.35 | 1.30E-10 | Illnesses of mother: Severe depression | Family history |
| 35133/1148(36281) | 0.26 | 0.14 | 0.38 | 2.39E-05 | Treatment speciality of consultant (recoded): Geriatric medicine | Summary Administration |
| 36057/1050(37107) | 0.25 | 0.13 | 0.38 | 6.70E-05 | Destinations on discharge from hospital (recoded): Transfer to other NHS provider: General ward, young physically disabled, A&E | Summary Administration |
| 34056/3728(37784) | 0.25 | 0.18 | 0.32 | 6.35E-12 | E78.0 Pure hypercholesterolaemia | Summary Diagnoses |
| 35502/2282(37784) | 0.24 | 0.15 | 0.33 | 4.47E-08 | Illnesses of siblings: Severe depression | Family history |
| 36135/1649(37784) | 0.24 | 0.14 | 0.34 | 4.00E-06 | I20.9 Angina pectoris, unspecified | Summary Diagnoses |
| 36001/1636(37637) | 0.24 | 0.13 | 0.34 | 4.90E-06 | Main speciality of consultant (recoded): Respiratory medicine | Summary Administration |
| 33063/4719(37782) | 0.23 | 0.17 | 0.29 | 6.35E-13 | Non-cancer illness code, self-reported: high cholesterol | Medical conditions |
| 33114/4538(37652) | 0.21 | 0.14 | 0.28 | 4.07E-09 | Medication for cholesterol, blood pressure or diabetes: Cholesterol lowering medication | Health and medical history |
| 35990/1794(37784) | 0.21 | 0.12 | 0.31 | 1.59E-05 | Z88.0 Personal history of allergy to penicillin | Summary Diagnoses |
| 35955/1672(37627) | 0.21 | 0.11 | 0.31 | 4.99E-05 | Sources of admission to hospital (recoded): Transfer to other NHS provider: General ward, young physically disabled, A&E | Summary Administration |
| 35620/2164(37784) | 0.21 | 0.12 | 0.29 | 4.67E-06 | R07.4 Chest pain, unspecified | Summary Diagnoses |
| 34988/2796(37784) | 0.20 | 0.12 | 0.28 | 4.15E-07 | J45.9 Asthma, unspecified | Summary Diagnoses |
| 34658/3126(37784) | 0.20 | 0.12 | 0.27 | 2.10E-07 | G45.9 Unspecified diagnostic fibreoptic endoscopic examination of upper gastrointestinal tract | Summary Operations |
| 11267/2249(13516) | 0.19 | 0.10 | 0.29 | 3.79E-05 | Ever highly irritable/argumentative for 2 days | Psychosocial factors |
| 26082/10718(36800) | 0.19 | 0.14 | 0.24 | 5.36E-16 | Treatment speciality of consultant (recoded): General medicine | Summary Administration |
| 30565/6143(36708) | 0.18 | 0.13 | 0.24 | 9.59E-11 | Tense / 'highly strung' | Psychosocial factors |
| 34809/2784(37593) | 0.17 | 0.10 | 0.25 | 1.21E-05 | Reason for glasses/contact lenses: For long-sightedness, i.e. for distance and near, but particularly for near tasks like reading (called 'hypermetropia') | Health and medical history |
| 34571/3213(37784) | 0.17 | 0.09 | 0.24 | 7.37E-06 | Y98.1 Radiology of one body area (or < 20 minutes) | Summary Operations |
| 35145/2489(37634) | 0.17 | 0.08 | 0.25 | 7.04E-05 | Main speciality of consultant (recoded): Accident and emergency (A&E) | Summary Administration |
| 27585/10118(37703) | 0.17 | 0.12 | 0.21 | 2.94E-12 | Main speciality of consultant (recoded): General medicine | Summary Administration |
| 32610/5047(37657) | 0.16 | 0.10 | 0.23 | 6.48E-07 | Main speciality of consultant (recoded): Gynaecology | Summary Administration |
| 34315/3469(37784) | 0.16 | 0.09 | 0.23 | 5.80E-06 | Z27.4 Duodenum | Summary Operations |
| 24838/12131(36969) | 0.16 | 0.12 | 0.20 | 1.25E-12 | Long-standing illness, disability or infirmity | Health and medical history |
| 11632/8789(20421) | 0.16 | 0.10 | 0.22 | 2.45E-07 | Ever used hormone-replacement therapy (HRT) | Sex-specific factors |
| 33935/3758(37693) | 0.16 | 0.08 | 0.23 | 2.32E-05 | Medication for cholesterol, blood pressure, diabetes, or take exogenous hormones: Blood pressure medication | Health and medical history |
| 29024/8366(37390) | 0.16 | 0.11 | 0.20 | 8.28E-10 | Medication for pain relief, constipation, heartburn: Paracetamol | Health and medical history |
| 31297/6213(37510) | 0.15 | 0.10 | 0.21 | 1.93E-07 | Qualifications | Sociodemographics |
| 27915/9686(37601) | 0.15 | 0.10 | 0.20 | 1.94E-10 | Illness, injury, bereavement, stress in last 2 years: Death of a close relative | Psychosocial factors |
| 24531/13033(37564) | 0.15 | 0.11 | 0.20 | 9.68E-12 | Seen doctor (GP) for nerves, anxiety, tension or depression | Psychosocial factors |
| 31036/6746(37782) | 0.15 | 0.10 | 0.20 | 3.04E-08 | Treatment/medication code: paracetamol | Medications |
| 31819/5622(37441) | 0.15 | 0.09 | 0.21 | 2.76E-07 | Chest pain or discomfort | Health and medical history |
| 33797/3799(37596) | 0.15 | 0.08 | 0.22 | 2.02E-05 | Illness, injury, bereavement, stress in last 2 years: Serious illness, injury or assault to yourself | Psychosocial factors |
| 29179/8605(37784) | 0.14 | 0.09 | 0.19 | 4.31E-08 | I10 Essential (primary) hypertension | Summary Diagnoses |
| 30885/6377(37262) | 0.13 | 0.08 | 0.19 | 2.25E-06 | Loneliness, isolation | Psychosocial factors |
| 31050/6653(37703) | 0.13 | 0.08 | 0.19 | 3.22E-06 | Mouth/teeth dental problems: Dentures | Health and medical history |
| 33411/4243(37654) | 0.13 | 0.07 | 0.20 | 6.17E-05 | Seen a psychiatrist for nerves, anxiety, tension or depression | Psychosocial factors |
| 32146/5547(37693) | 0.13 | 0.07 | 0.19 | 8.25E-06 | Other eye problems | Health and medical history |
| 29596/7565(37161) | 0.13 | 0.08 | 0.18 | 5.85E-07 | Wheeze or whistling in the chest in last year | Health and medical history |
| 32689/5093(37782) | 0.13 | 0.07 | 0.19 | 4.37E-05 | Treatment/medication code: aspirin | Medications |
| 28708/9010(37718) | 0.13 | 0.08 | 0.17 | 1.81E-07 | Pain type(s) experienced in last month: Neck or shoulder pain | Health and medical history |
| 35854 | 0.13 | 0.10 | 0.15 | 4.17E-32 | Apolipoprotein B | Blood assays |
| 31905/5691(37596) | 0.12 | 0.07 | 0.18 | 1.83E-05 | Reason for glasses/contact lenses: For just reading/near work as you are getting older (called 'presbyopia') | Health and medical history |
| 27188/10518(37706) | 0.12 | 0.08 | 0.17 | 1.46E-07 | Vascular/heart problems diagnosed by doctor: High blood pressure | Health and medical history |
| 26699/11052(37751) | 0.12 | 0.08 | 0.17 | 1.15E-07 | Methods of admission to hospital (recoded): Emergency admission: A&E | Summary Administration |
| 24342/13126(37468) | 0.12 | 0.08 | 0.16 | 3.38E-08 | Eye problems/disorders | Health and medical history |
| 31805/5584(37389) | 0.12 | 0.06 | 0.18 | 6.26E-05 | Medication for pain relief, constipation, heartburn: Aspirin | Health and medical history |
| 20728/16169(36897) | 0.12 | 0.08 | 0.16 | 1.93E-08 | Mood swings | Psychosocial factors |
| 32483/5113(37596) | 0.12 | 0.06 | 0.18 | 9.27E-05 | Illness, injury, bereavement, stress in last 2 years: Serious illness, injury or assault of a close relative | Psychosocial factors |
| 19775/17931(37706) | 0.12 | 0.08 | 0.16 | 1.84E-08 | Taking other prescription medications | Health and medical history |
| 27901/9881(37782) | 0.12 | 0.07 | 0.16 | 1.11E-06 | Non-cancer illness code, self-reported: hypertension | Medical conditions |
| 7061/30674(37735) | 0.11 | 0.06 | 0.17 | 2.33E-05 | Destinations on discharge from hospital (recoded): Usual Place of residence | Summary Administration |
| 27721/9996(37717) | 0.11 | 0.07 | 0.16 | 1.58E-06 | Pain type(s) experienced in last month: Back pain | Health and medical history |
| 7000/30781(37781) | 0.11 | 0.06 | 0.16 | 4.63E-05 | Methods of discharge from hospital (recoded): Discharged on clinical advice/consent | Summary Administration |
| 14752/23032(37784) | 0.11 | 0.07 | 0.15 | 4.37E-07 | Patient classification on admission (recoded): Inpatient | Summary Administration |
| 29187/7974(37161) | 0.11 | 0.06 | 0.16 | 3.98E-05 | Other serious medical condition/disability diagnosed by doctor | Health and medical history |

**Table S5-B. PheWAS results, Beta <= -0.1 using PRS calculated from Jansen et *al*. including *APOE* locus.**

| **n** | **Beta** | **Lower CI 95** | **Upper CI 95** | ***p*-value** | **Trait/Description** | **Category** |
| --- | --- | --- | --- | --- | --- | --- |
| **29802/7520(37322)** | **-0.51** | **-0.57** | **-0.45** | **1.44E-65** | **Father still alive** | **Family history** |
| **23734/13761(37495)** | **-0.51** | **-0.55** | **-0.46** | **3.42E-94** | **Mother still alive** | **Family history** |
| 27111/10673(37784) | -0.25 | -0.30 | -0.20 | 6.20E-27 | Illnesses of mother: High blood pressure | Family history |
| 29524 | -0.24 | -0.27 | -0.22 | 2.96E-100 | Father's age at death | Family history |
| 36124/1660(37784) | -0.24 | -0.34 | -0.14 | 2.84E-06 | Illnesses of mother: Bowel cancer | Family history |
| 34976/2808(37784) | -0.20 | -0.27 | -0.12 | 8.83E-07 | Illnesses of father: Prostate cancer | Family history |
| 30697/7087(37784) | -0.18 | -0.23 | -0.12 | 5.06E-11 | Illnesses of mother: Heart disease | Family history |
| 26283/11380(37663) | -0.16 | -0.23 | -0.09 | 2.83E-06 | Medication for cholesterol, blood pressure or diabetes | Health and medical history |
| 24797/12708(37505) | -0.15 | -0.19 | -0.10 | 2.49E-11 | Qualifications: College or University degree | Sociodemographics |
| 23480/14231(37711) | -0.14 | -0.20 | -0.08 | 5.98E-06 | Medication for cholesterol, blood pressure, diabetes, or take exogenous hormones | Health and medical history |
| 11206/26504(37710) | -0.12 | -0.17 | -0.07 | 3.21E-07 | Vascular/heart problems diagnosed by doctor | Health and medical history |
| 7486 | -0.11 | -0.14 | -0.08 | 5.96E-12 | Father's age | Family history |

**Table S5-C. PheWAS results, Beta >= 0.1 using PRS calculated from Jansen et *al*. excluding *APOE* locus.**

| **n** | **Beta** | **Lower CI 95** | **Upper CI 95** | ***p*-value** | **Trait/Description** | **Category** |
| --- | --- | --- | --- | --- | --- | --- |
| **27386/10398(37784)** | **4.37** | **4.25** | **4.50** | **0.00E+00** | **Illnesses of mother: Alzheimer's disease/dementia** | **Family history** |
| **32258/5526(37784)** | **3.95** | **3.79** | **4.12** | **0.00E+00** | **Illnesses of father: Alzheimer's disease/dementia** | **Family history** |
| **37479/305(37784)** | **2.89** | **2.42** | **3.43** | **3.74E-29** | **Diagnoses - ICD10: G30.9 Alzheimer's disease, unspecified** | **Summary Diagnoses** |
| **37475/309(37784)** | **1.09** | **0.83** | **1.36** | **4.36E-16** | **Illnesses of siblings: Alzheimer's disease/dementia** | **Family history** |
| **37555/229(37784)** | **0.57** | **0.30** | **0.85** | **4.67E-05** | **Diagnoses - ICD10: R15 Faecal incontinence** | **Summary Diagnoses** |
| **37429/355(37784)** | **0.54** | **0.32** | **0.76** | **1.45E-06** | **Diagnoses - ICD10: R29.6 Tendency to fall, not elsewhere classified** | **Summary Diagnoses** |
| **37482/298(37780)** | **0.50** | **0.27** | **0.74** | **3.20E-05** | **Treatment/medication code: ginkgo forte tablet** | **Medications** |
| **37472/312(37784)** | **0.46** | **0.23** | **0.69** | **1.13E-04** | **Diagnoses - ICD10: R41.0 Disorientation, unspecified** | **Summary Diagnoses** |
| **36617/1167(37784)** | **0.45** | **0.33** | **0.57** | **1.08E-13** | **Illnesses of father: Parkinson's disease** | **Family history** |
| **37044/740(37784)** | **0.45** | **0.30** | **0.60** | **5.69E-09** | **Illnesses of mother: Parkinson's disease** | **Family history** |
| 37475/309(37784) | 0.41 | 0.18 | 0.64 | 4.96E-04 | Diagnoses - ICD10: R32 Unspecified urinary incontinence | Summary Diagnoses |
| 37522/258(37780) | 0.41 | 0.16 | 0.67 | 1.58E-03 | Treatment/medication code: clopidogrel | Medications |
| 37542/238(37780) | 0.39 | 0.13 | 0.66 | 3.70E-03 | Non-cancer illness code, self-reported: gastric/stomach ulcers | Medical conditions |
| 37525/259(37784) | 0.37 | 0.12 | 0.62 | 3.51E-03 | Diagnoses - ICD10: Y43.3 Other antineoplastic drugs | Summary Diagnoses |
| 37258/522(37780) | 0.36 | 0.19 | 0.54 | 6.56E-05 | Non-cancer illness code, self-reported: emphysema/chronic bronchitis | Medical conditions |
| 37516/264(37780) | 0.36 | 0.11 | 0.61 | 4.62E-03 | Non-cancer illness code, self-reported: chronic sinusitis | Medical conditions |
| 37368/412(37780) | 0.36 | 0.16 | 0.56 | 5.03E-04 | Treatment/medication code: tramadol | Medications |
| 37572/212(37784) | 0.35 | 0.08 | 0.63 | 1.23E-02 | PCT responsible for patient data: DERBY CITY PCT | Summary Administration |
| 37552/228(37780) | 0.34 | 0.08 | 0.61 | 1.14E-02 | Treatment/medication code: rosuvastatin | Medications |
| 37368/416(37784) | 0.34 | 0.15 | 0.54 | 6.54E-04 | Diagnoses - ICD10: A41.9 Septicaemia, unspecified | Summary Diagnoses |
| 37570/214(37784) | 0.34 | 0.07 | 0.62 | 1.40E-02 | Operative procedures - OPCS4: C90.9 Unspecified local anaesthetics for ophthalmology procedures | Summary Operations |
| 37516/264(37780) | 0.34 | 0.10 | 0.59 | 6.89E-03 | Operation code: coronary angiogram | Operations |
| 37328/456(37784) | 0.34 | 0.15 | 0.53 | 4.56E-04 | Diagnoses - ICD10: M19.99 Arthrosis, unspecified (Site unspecified) | Summary Diagnoses |
| 37459/321(37780) | 0.33 | 0.11 | 0.56 | 3.60E-03 | Treatment/medication code: felodipine | Medications |
| 37476/308(37784) | 0.33 | 0.11 | 0.56 | 4.28E-03 | PCT responsible for patient data: ROTHERHAM PCT | Summary Administration |
| 37446/334(37780) | 0.33 | 0.11 | 0.55 | 3.43E-03 | Non-cancer illness code, self-reported: ear/vestibular disorder | Medical conditions |
| 37367/227(37594) | 0.32 | 0.06 | 0.59 | 1.65E-02 | Reason for glasses/contact lenses: Other eye condition | Health and medical history |
| 37207/229(37436) | 0.32 | 0.06 | 0.59 | 1.67E-02 | Eye problems/disorders: Injury or trauma resulting in loss of vision | Health and medical history |
| 37071/565(37636) | 0.32 | 0.15 | 0.49 | 1.83E-04 | Main speciality of consultant (recoded): Rheumatology | Summary Administration |
| 37447/333(37780) | 0.32 | 0.10 | 0.54 | 3.97E-03 | Non-cancer illness code, self-reported: arthritis (nos) | Medical conditions |
| 36383/1401(37784) | 0.32 | 0.21 | 0.43 | 8.38E-09 | Illnesses of father: Severe depression | Family history |
| 37530/254(37784) | 0.32 | 0.07 | 0.57 | 1.41E-02 | PCT responsible for patient data: ASHTON, LEIGH AND WIGAN PCT | Summary Administration |
| 37507/277(37784) | 0.31 | 0.07 | 0.56 | 1.17E-02 | Diagnoses - ICD10: R26.8 Other and unspecified abnormalities of gait and mobility | Summary Diagnoses |
| 37554/230(37784) | 0.31 | 0.05 | 0.58 | 2.24E-02 | Diagnoses - ICD10: D12.2 Ascending colon | Summary Diagnoses |
| 37330/454(37784) | 0.31 | 0.12 | 0.50 | 1.35E-03 | Operative procedures - OPCS4: V54.4 Injection around spinal facet of spine | Summary Operations |
| 37118/666(37784) | 0.31 | 0.15 | 0.47 | 1.23E-04 | Diagnoses - ICD10: I25.8 Other forms of chronic ischaemic heart disease | Summary Diagnoses |
| 37532/252(37784) | 0.31 | 0.05 | 0.56 | 1.75E-02 | Diagnoses - ICD10: G43.9 Migraine, unspecified | Summary Diagnoses |
| 36612/1168(37780) | 0.30 | 0.18 | 0.42 | 7.61E-07 | Treatment/medication code: atorvastatin | Medications |
| 5870/413(6283) | 0.30 | 0.10 | 0.51 | 4.07E-03 | Number of stillbirths | Sex-specific factors |
| 37553/231(37784) | 0.30 | 0.03 | 0.56 | 2.85E-02 | Diagnoses - ICD10: M54.56 Low back pain (Lumbar region) | Summary Diagnoses |
| 37582/202(37784) | 0.29 | 0.01 | 0.58 | 4.13E-02 | Diagnoses - ICD10: J96.9 Respiratory failure, unspecified | Summary Diagnoses |
| 36873/911(37784) | 0.29 | 0.16 | 0.43 | 2.02E-05 | Operative procedures - OPCS4: K63.3 Angiocardiography of left side of heart NEC | Summary Operations |
| 37057/668(37725) | 0.29 | 0.14 | 0.45 | 2.54E-04 | Blood clot, DVT, bronchitis, emphysema, asthma, rhinitis, eczema, allergy diagnosed by doctor: Emphysema/chronic bronchitis | Health and medical history |
| 37564/220(37784) | 0.29 | 0.02 | 0.56 | 3.64E-02 | Diagnoses - ICD10: Z03.4 Observation for suspected myocardial infarction | Summary Diagnoses |
| 36482/1226(37708) | 0.29 | 0.17 | 0.41 | 1.46E-06 | Vascular/heart problems diagnosed by doctor: Angina | Health and medical history |
| 37281/503(37784) | 0.29 | 0.11 | 0.47 | 1.60E-03 | Diagnoses - ICD10: G47.3 Sleep apnoea | Summary Diagnoses |
| 37448/336(37784) | 0.29 | 0.07 | 0.51 | 1.00E-02 | Diagnoses - ICD10: N18.3 Chronic kidney disease, stage 3 | Summary Diagnoses |
| 37455/329(37784) | 0.29 | 0.07 | 0.51 | 1.09E-02 | PCT responsible for patient data: STOKE ON TRENT PCT | Summary Administration |
| 36593/1187(37780) | 0.28 | 0.16 | 0.40 | 3.22E-06 | Non-cancer illness code, self-reported: angina | Medical conditions |
| 37427/357(37784) | 0.28 | 0.07 | 0.50 | 9.49E-03 | PCT responsible for patient data: KIRKLEES PCT | Summary Administration |
| 37076/568(37644) | 0.28 | 0.11 | 0.45 | 1.15E-03 | Methods of discharge from hospital (recoded): Patient death | Summary Administration |
| 37300/484(37784) | 0.28 | 0.10 | 0.47 | 2.87E-03 | Diagnoses - ICD10: Z95.1 Presence of aortocoronary bypass graft | Summary Diagnoses |
| 37569/215(37784) | 0.28 | 0.01 | 0.55 | 4.31E-02 | Operative procedures - OPCS4: Z41.3 Ureter NEC | Summary Operations |
| 37190/594(37784) | 0.28 | 0.11 | 0.45 | 9.18E-04 | Diagnoses - ICD10: M13.9 Arthritis, unspecified | Summary Diagnoses |
| 37507/273(37780) | 0.28 | 0.04 | 0.52 | 2.42E-02 | Treatment/medication code: irbesartan | Medications |
| 35270/2514(37784) | 0.28 | 0.20 | 0.36 | 2.91E-11 | Illnesses of mother: Severe depression | Family history |
| 37567/217(37784) | 0.28 | 0.01 | 0.55 | 4.45E-02 | Operative procedures - OPCS4: Z41.1 Kidney | Summary Operations |
| 36363/1421(37784) | 0.28 | 0.17 | 0.38 | 5.86E-07 | Diagnoses - ICD10: N39.0 Urinary tract infection, site not specified | Summary Diagnoses |
| 37579/205(37784) | 0.28 | 0.00 | 0.56 | 5.35E-02 | Diagnoses - ICD10: I21.1 Acute transmural myocardial infarction of inferior wall | Summary Diagnoses |
| 36887/893(37780) | 0.28 | 0.14 | 0.41 | 7.00E-05 | Treatment/medication code: co-codamol | Medications |
| 36463/1321(37784) | 0.27 | 0.16 | 0.39 | 1.44E-06 | Diagnoses - ICD10: F32.9 Depressive episode, unspecified | Summary Diagnoses |
| 37559/225(37784) | 0.27 | 0.01 | 0.54 | 4.49E-02 | Diagnoses - ICD10: R12 Heartburn | Summary Diagnoses |
| 37551/229(37780) | 0.27 | 0.01 | 0.54 | 4.47E-02 | Treatment/medication code: propranolol | Medications |
| 37290/494(37784) | 0.27 | 0.09 | 0.45 | 3.42E-03 | Diagnoses - ICD10: Z51.5 Palliative care | Summary Diagnoses |
| 37572/212(37784) | 0.27 | 0.00 | 0.55 | 5.34E-02 | Operative procedures - OPCS4: X71.5 Procurement of drugs for chemotherapy for neoplasm for regimens in Band 10 | Summary Operations |
| 37359/425(37784) | 0.27 | 0.08 | 0.46 | 6.57E-03 | Diagnoses - ICD10: Z92.6 Personal history of chemotherapy for neoplastic disease | Summary Diagnoses |
| 37066/718(37784) | 0.27 | 0.12 | 0.42 | 4.74E-04 | Diagnoses - ICD10: R13 Dysphagia | Summary Diagnoses |
| 37523/261(37784) | 0.27 | 0.02 | 0.52 | 3.39E-02 | Diagnoses - ICD10: Z50.1 Other physical therapy | Summary Diagnoses |
| 37298/486(37784) | 0.27 | 0.09 | 0.45 | 4.09E-03 | Operative procedures - OPCS4: A55.9 Unspecified diagnostic spinal puncture | Summary Operations |
| 37477/307(37784) | 0.27 | 0.04 | 0.50 | 2.29E-02 | Diagnoses - ICD10: K29.5 Chronic gastritis, unspecified | Summary Diagnoses |
| 37367/417(37784) | 0.26 | 0.07 | 0.46 | 8.29E-03 | Diagnoses - ICD10: M54.5 Low back pain | Summary Diagnoses |
| 36129/1655(37784) | 0.26 | 0.16 | 0.37 | 3.64E-07 | Diagnoses - ICD10: I20.9 Angina pectoris, unspecified | Summary Diagnoses |
| 36533/1251(37784) | 0.26 | 0.15 | 0.38 | 6.80E-06 | Diagnoses - ICD10: D64.9 Anaemia, unspecified | Summary Diagnoses |
| 37571/213(37784) | 0.26 | -0.01 | 0.54 | 5.94E-02 | Diagnoses - ICD10: K13.7 Other and unspecified lesions of oral mucosa | Summary Diagnoses |
| 37358/426(37784) | 0.26 | 0.07 | 0.46 | 8.23E-03 | Diagnoses - ICD10: Z92.3 Personal history of irradiation | Summary Diagnoses |
| 37366/418(37784) | 0.26 | 0.07 | 0.46 | 8.93E-03 | Diagnoses - ICD10: M13.99 Arthritis, unspecified (Site unspecified) | Summary Diagnoses |
| 37570/214(37784) | 0.26 | -0.01 | 0.54 | 6.06E-02 | Diagnoses - ICD10: C50.4 Upper-outer quadrant of breast | Summary Diagnoses |
| 37579/205(37784) | 0.26 | -0.02 | 0.54 | 6.71E-02 | Diagnoses - ICD10: D17.1 Benign lipomatous neoplasm of skin and subcutaneous tissue of trunk | Summary Diagnoses |
| 37519/265(37784) | 0.26 | 0.02 | 0.51 | 3.76E-02 | Diagnoses - ICD10: E87.1 Hypo-osmolality and hyponatraemia | Summary Diagnoses |
| 37534/250(37784) | 0.26 | 0.01 | 0.52 | 4.54E-02 | Diagnoses - ICD10: Z02.8 Other examinations for administrative purposes | Summary Diagnoses |
| 37562/222(37784) | 0.26 | -0.01 | 0.53 | 5.77E-02 | Illnesses of siblings: Parkinson's disease | Family history |
| 4686/916(5602) | 0.26 | 0.11 | 0.41 | 6.06E-04 | Chest pain or discomfort walking normally | Health and medical history |
| 37149/631(37780) | 0.26 | 0.10 | 0.42 | 1.46E-03 | Treatment/medication code: citalopram | Medications |
| 37387/397(37784) | 0.26 | 0.06 | 0.46 | 1.11E-02 | Diagnoses - ICD10: M23.23 Derangement of meniscus due to old tear or injury (Medial collateral ligament or Other and unspecified medial meniscus) | Summary Diagnoses |
| 37471/313(37784) | 0.26 | 0.03 | 0.49 | 2.48E-02 | Diagnoses - ICD10: M54.9 Dorsalgia, unspecified | Summary Diagnoses |
| 37431/353(37784) | 0.26 | 0.05 | 0.47 | 1.71E-02 | Diagnoses - ICD10: M51.1 Lumbar and other intervertebral disk disorders with radiculopathy | Summary Diagnoses |
| 35787/455(36242) | 0.26 | 0.07 | 0.45 | 7.14E-03 | Treatment speciality of consultant (recoded): Rheumatology | Summary Administration |
| 37508/276(37784) | 0.26 | 0.02 | 0.50 | 3.65E-02 | Diagnoses - ICD10: C79.5 Secondary malignant neoplasm of bone and bone marrow | Summary Diagnoses |
| 37384/400(37784) | 0.26 | 0.06 | 0.46 | 1.21E-02 | Diagnoses - ICD10: H91.9 Hearing loss, unspecified | Summary Diagnoses |
| 37470/314(37784) | 0.25 | 0.03 | 0.48 | 2.68E-02 | Operative procedures - OPCS4: U21.1 Magnetic resonance imaging NEC | Summary Operations |
| 37324/456(37780) | 0.25 | 0.07 | 0.44 | 7.91E-03 | Treatment/medication code: salbutamol | Medications |
| 36928/856(37784) | 0.25 | 0.11 | 0.39 | 4.44E-04 | Diagnoses - ICD10: I25.2 Old myocardial infarction | Summary Diagnoses |
| 37499/285(37784) | 0.25 | 0.01 | 0.49 | 4.25E-02 | PCT responsible for patient data: BOLTON PCT | Summary Administration |
| 36533/1251(37784) | 0.25 | 0.13 | 0.36 | 2.43E-05 | Manic/hyper symptoms | Psychosocial factors |
| 37486/298(37784) | 0.25 | 0.02 | 0.48 | 3.72E-02 | Operative procedures - OPCS4: Z67.5 Lumbar intervertebral joint | Summary Operations |
| 35494/2290(37784) | 0.25 | 0.16 | 0.33 | 2.14E-08 | Illnesses of siblings: Severe depression | Family history |
| 37123/661(37784) | 0.25 | 0.09 | 0.40 | 2.08E-03 | Diagnoses - ICD10: R06.0 Dyspnoea | Summary Diagnoses |
| 36878/906(37784) | 0.25 | 0.11 | 0.38 | 3.36E-04 | Diagnoses - ICD10: R10.1 Pain localised to upper abdomen | Summary Diagnoses |
| 37533/251(37784) | 0.24 | -0.01 | 0.51 | 6.52E-02 | Operative procedures - OPCS4: Y90.3 Scanning NEC | Summary Operations |
| 37290/494(37784) | 0.24 | 0.06 | 0.43 | 8.43E-03 | Diagnoses - ICD10: I20.0 Unstable angina | Summary Diagnoses |
| 36487/1297(37784) | 0.24 | 0.13 | 0.36 | 3.32E-05 | Diagnoses - ICD10: I25.9 Chronic ischaemic heart disease, unspecified | Summary Diagnoses |
| 37408/372(37780) | 0.24 | 0.03 | 0.45 | 2.36E-02 | Operation code: anterior/posterior repair bladder/uterus | Operations |
| 37433/351(37784) | 0.24 | 0.03 | 0.45 | 2.70E-02 | Diagnoses - ICD10: G55.1 Nerve root and plexus compressions in intervertebral disk disorders | Summary Diagnoses |
| 37272/512(37784) | 0.24 | 0.06 | 0.42 | 8.47E-03 | Diagnoses - ICD10: N81.1 Cystocele | Summary Diagnoses |
| 37459/325(37784) | 0.24 | 0.02 | 0.46 | 3.69E-02 | PCT responsible for patient data: GATESHEAD PCT | Summary Administration |
| 36961/823(37784) | 0.24 | 0.10 | 0.38 | 8.50E-04 | Manic/hyper symptoms: I was more active than usual | Psychosocial factors |
| 37407/373(37780) | 0.24 | 0.03 | 0.45 | 2.50E-02 | Operation code: coronary angioplasty (ptca) +/- stent | Operations |
| 37504/280(37784) | 0.24 | 0.00 | 0.48 | 5.53E-02 | Diagnoses - ICD10: J44.0 Chronic obstructive pulmonary disease with acute lower respiratory infection | Summary Diagnoses |
| 37523/261(37784) | 0.24 | -0.01 | 0.48 | 6.17E-02 | Operative procedures - OPCS4: U07.1 Computed tomography of chest | Summary Operations |
| 34959/2740(37699) | 0.23 | 0.15 | 0.32 | 3.99E-08 | Medication for cholesterol, blood pressure, diabetes, or take exogenous hormones: Cholesterol lowering medication | Health and medical history |
| 35139/1128(36267) | 0.23 | 0.11 | 0.36 | 1.54E-04 | Treatment speciality of consultant (recoded): Geriatric medicine | Summary Administration |
| 37558/226(37784) | 0.23 | -0.03 | 0.50 | 8.58E-02 | Diagnoses - ICD10: E14.9 Without complications | Summary Diagnoses |
| 37578/206(37784) | 0.23 | -0.04 | 0.51 | 1.00E-01 | Diagnoses - ICD10: M20.5 Other deformities of toe(s) (acquired) | Summary Diagnoses |
| 37561/223(37784) | 0.23 | -0.04 | 0.50 | 8.94E-02 | Operative procedures - OPCS4: Y81.1 Epidural anaesthetic using lumbar approach | Summary Operations |
| 37499/285(37784) | 0.23 | -0.01 | 0.47 | 5.65E-02 | Operative procedures - OPCS4: Z28.8 Specified large intestine NEC | Summary Operations |
| 37157/627(37784) | 0.23 | 0.07 | 0.39 | 4.88E-03 | Diagnoses - ICD10: K92.2 Gastro-intestinal haemorrhage, unspecified | Summary Diagnoses |
| 37512/272(37784) | 0.23 | -0.01 | 0.47 | 6.51E-02 | Diagnoses - ICD10: I73.9 Peripheral vascular disease, unspecified | Summary Diagnoses |
| 37569/215(37784) | 0.23 | -0.04 | 0.50 | 1.02E-01 | Operative procedures - OPCS4: W90.1 Aspiration of joint | Summary Operations |
| 36846/862(37708) | 0.23 | 0.09 | 0.37 | 1.28E-03 | Vascular/heart problems diagnosed by doctor: Heart attack | Health and medical history |
| 37523/261(37784) | 0.23 | -0.02 | 0.48 | 7.27E-02 | Diagnoses - ICD10: I47.1 Supraventricular tachycardia | Summary Diagnoses |
| 36568/1216(37784) | 0.23 | 0.11 | 0.34 | 1.37E-04 | Diagnoses - ICD10: R11 Nausea and vomiting | Summary Diagnoses |
| 37519/261(37780) | 0.23 | -0.02 | 0.47 | 7.33E-02 | Treatment/medication code: lipitor 10mg tablet | Medications |
| 37420/364(37784) | 0.23 | 0.02 | 0.44 | 3.49E-02 | Operative procedures - OPCS4: X72.4 Delivery of subsequent element of cycle of chemotherapy for neoplasm | Summary Operations |
| 37350/430(37780) | 0.22 | 0.03 | 0.42 | 2.22E-02 | Treatment/medication code: cetirizine | Medications |
| 37214/570(37784) | 0.22 | 0.06 | 0.39 | 9.15E-03 | Diagnoses - ICD10: K58.9 Irritable bowel syndrome without diarrhoea | Summary Diagnoses |
| 37280/504(37784) | 0.22 | 0.05 | 0.40 | 1.41E-02 | Operative procedures - OPCS4: U05.2 Magnetic resonance imaging of head | Summary Operations |
| 37366/253(37619) | 0.22 | -0.03 | 0.47 | 8.13E-02 | Sources of admission to hospital (recoded): Temporary place of residence | Summary Administration |
| 36168/1616(37784) | 0.22 | 0.12 | 0.32 | 1.78E-05 | Operative procedures - OPCS4: U05.1 Computed tomography of head | Summary Operations |
| 37085/699(37784) | 0.22 | 0.07 | 0.37 | 4.48E-03 | Diagnoses - ICD10: N17.9 Acute renal failure, unspecified | Summary Diagnoses |
| 37348/432(37780) | 0.22 | 0.03 | 0.41 | 2.45E-02 | Treatment/medication code: fluoxetine | Medications |
| 37479/301(37780) | 0.22 | -0.01 | 0.45 | 5.99E-02 | Non-cancer illness code, self-reported: muscle/soft tissue problem | Medical conditions |
| 37570/214(37784) | 0.22 | -0.05 | 0.49 | 1.14E-01 | Operative procedures - OPCS4: M61.1 Total excision of prostate and capsule of prostate | Summary Operations |
| 36040/1604(37644) | 0.22 | 0.12 | 0.32 | 2.22E-05 | Main speciality of consultant (recoded): Respiratory medicine | Summary Administration |
| 36093/1031(37124) | 0.22 | 0.09 | 0.35 | 6.22E-04 | Destinations on discharge from hospital (recoded): Transfer to other NHS provider: General ward, young physically disabled, A&E | Summary Administration |
| 37548/232(37780) | 0.22 | -0.04 | 0.48 | 9.88E-02 | Operation code: rhinoplasty / nose surgery | Operations |
| 226/1312(1538) | 0.22 | -0.07 | 0.51 | 1.35E-01 | Death record format | Death register |
| 37532/248(37780) | 0.22 | -0.03 | 0.47 | 9.00E-02 | Treatment/medication code: salbutamol 100micrograms spacehaler | Medications |
| 37479/305(37784) | 0.22 | -0.01 | 0.45 | 6.21E-02 | Diagnoses - ICD10: Z13.9 Special screening examination, unspecified | Summary Diagnoses |
| 36943/837(37780) | 0.22 | 0.08 | 0.36 | 2.36E-03 | Non-cancer illness code, self-reported: heart attack/myocardial infarction | Medical conditions |
| 37546/238(37784) | 0.22 | -0.04 | 0.48 | 9.92E-02 | Diagnoses - ICD10: B95.6 Staphylococcus aureus as the cause of diseases classified to other chapters | Summary Diagnoses |
| 37572/208(37780) | 0.22 | -0.06 | 0.49 | 1.22E-01 | Non-cancer illness code, self-reported: back pain | Medical conditions |
| 37399/385(37784) | 0.22 | 0.01 | 0.42 | 3.75E-02 | Diagnoses - ICD10: E86 Volume depletion | Summary Diagnoses |
| 36736/896(37632) | 0.22 | 0.08 | 0.35 | 1.63E-03 | Vitamin and mineral supplements: Folic acid or Folate (Vit B9) | Health and medical history |
| 37571/213(37784) | 0.22 | -0.06 | 0.49 | 1.22E-01 | Diagnoses - ICD10: C78.0 Secondary malignant neoplasm of lung | Summary Diagnoses |
| 37573/207(37780) | 0.22 | -0.06 | 0.49 | 1.28E-01 | Treatment/medication code: mebeverine | Medications |
| 37562/222(37784) | 0.21 | -0.05 | 0.48 | 1.18E-01 | Diagnoses - ICD10: M10.9 Gout, unspecified | Summary Diagnoses |
| 37437/347(37784) | 0.21 | 0.00 | 0.43 | 5.03E-02 | Diagnoses - ICD10: G40.9 Epilepsy, unspecified | Summary Diagnoses |
| 37535/249(37784) | 0.21 | -0.04 | 0.47 | 9.94E-02 | Operative procedures - OPCS4: Z42.1 Bladder NEC | Summary Operations |
| 35038/2746(37784) | 0.21 | 0.13 | 0.29 | 1.39E-07 | Diagnoses - ICD10: J45.9 Asthma, unspecified | Summary Diagnoses |
| 37565/219(37784) | 0.21 | -0.06 | 0.48 | 1.27E-01 | Diagnoses - ICD10: K56.6 Other and unspecified intestinal obstruction | Summary Diagnoses |
| 37141/643(37784) | 0.21 | 0.05 | 0.37 | 8.92E-03 | Diagnoses - ICD10: R63.4 Abnormal weight loss | Summary Diagnoses |
| 37341/443(37784) | 0.21 | 0.02 | 0.40 | 3.03E-02 | Diagnoses - ICD10: T81.4 Infection following a procedure, not elsewhere classified | Summary Diagnoses |
| 37534/246(37780) | 0.21 | -0.04 | 0.47 | 1.06E-01 | Operation code: mammoplasty/cosmetic operation on breast | Operations |
| 37386/398(37784) | 0.21 | 0.01 | 0.41 | 4.01E-02 | Operative procedures - OPCS4: X29.8 Other specified continuous Infusion of therapeutic substance | Summary Operations |
| 37575/205(37780) | 0.21 | -0.07 | 0.49 | 1.44E-01 | Operation code: stomach surgery | Operations |
| 37342/438(37780) | 0.21 | 0.02 | 0.40 | 3.34E-02 | Treatment/medication code: doxazosin | Medications |
| 37291/489(37780) | 0.20 | 0.02 | 0.39 | 2.74E-02 | Operation code: knee replacement/revision | Operations |
| 37484/300(37784) | 0.20 | -0.03 | 0.44 | 8.54E-02 | PCT responsible for patient data: HALTON AND ST HELENS PCT | Summary Administration |
| 36751/1033(37784) | 0.20 | 0.08 | 0.33 | 1.52E-03 | Illnesses of siblings: Stroke | Family history |
| 37145/290(37435) | 0.20 | -0.03 | 0.44 | 9.16E-02 | Eye problems/disorders: Diabetes related eye disease | Health and medical history |
| 37500/280(37780) | 0.20 | -0.04 | 0.44 | 9.69E-02 | Treatment/medication code: quinine | Medications |
| 11278/2276(13554) | 0.20 | 0.11 | 0.29 | 1.88E-05 | Ever highly irritable/argumentative for 2 days | Psychosocial factors |
| 37570/214(37784) | 0.20 | -0.07 | 0.48 | 1.49E-01 | Diagnoses - ICD10: R06.5 Mouth breathing | Summary Diagnoses |
| 37549/235(37784) | 0.20 | -0.06 | 0.46 | 1.29E-01 | Operative procedures - OPCS4: W84.8 Other specified therapeutic endoscopic operations on other joint structure | Summary Operations |
| 37513/267(37780) | 0.20 | -0.04 | 0.45 | 1.08E-01 | Non-cancer illness code, self-reported: cervical spondylosis | Medical conditions |
| 37553/227(37780) | 0.20 | -0.06 | 0.47 | 1.37E-01 | Operation code: elbow surgery | Operations |
| 37180/604(37784) | 0.20 | 0.04 | 0.36 | 1.70E-02 | Illnesses of siblings: Prostate cancer | Family history |
| 36004/1780(37784) | 0.20 | 0.10 | 0.29 | 5.43E-05 | Diagnoses - ICD10: Z88.0 Personal history of allergy to penicillin | Summary Diagnoses |
| 36927/857(37784) | 0.20 | 0.06 | 0.34 | 4.81E-03 | Diagnoses - ICD10: M19.9 Arthrosis, unspecified | Summary Diagnoses |
| 37490/294(37784) | 0.20 | -0.04 | 0.43 | 9.79E-02 | Diagnoses - ICD10: I51.7 Cardiomegaly | Summary Diagnoses |
| 37455/329(37784) | 0.20 | -0.02 | 0.42 | 7.99E-02 | PCT responsible for patient data: CENTRAL AND EASTERN CHESHIRE PCT | Summary Administration |
| 37521/263(37784) | 0.20 | -0.05 | 0.44 | 1.18E-01 | Diagnoses - ICD10: R00.0 Tachycardia, unspecified | Summary Diagnoses |
| 37579/205(37784) | 0.20 | -0.08 | 0.48 | 1.70E-01 | Operative procedures - OPCS4: K49.1 Percutaneous transluminal balloon angioplasty of one coronary artery | Summary Operations |
| 37174/606(37780) | 0.20 | 0.03 | 0.36 | 1.89E-02 | Treatment/medication code: ranitidine | Medications |
| 37584/200(37784) | 0.20 | -0.09 | 0.48 | 1.74E-01 | Diagnoses - ICD10: M54.59 Low back pain (Site unspecified) | Summary Diagnoses |
| 37544/236(37780) | 0.20 | -0.06 | 0.46 | 1.40E-01 | Non-cancer illness code, self-reported: bladder problem (not cancer) | Medical conditions |
| 37104/676(37780) | 0.19 | 0.04 | 0.35 | 1.30E-02 | Non-cancer illness code, self-reported: prolapsed disc/slipped disc | Medical conditions |
| 1701/423(2124) | 0.19 | -0.02 | 0.41 | 7.92E-02 | Length of longest manic/irritable episode | Psychosocial factors |
| 37413/371(37784) | 0.19 | -0.01 | 0.40 | 6.64E-02 | Diagnoses - ICD10: H40.9 Glaucoma, unspecified | Summary Diagnoses |
| 37541/243(37784) | 0.19 | -0.06 | 0.45 | 1.38E-01 | Diagnoses - ICD10: L90.5 Scar conditions and fibrosis of skin | Summary Diagnoses |
| 37542/242(37784) | 0.19 | -0.06 | 0.45 | 1.38E-01 | Operative procedures - OPCS4: Z92.7 Trunk NEC | Summary Operations |
| 37380/400(37780) | 0.19 | -0.01 | 0.39 | 5.82E-02 | Non-cancer illness code, self-reported: rheumatoid arthritis | Medical conditions |
| 36048/1736(37784) | 0.19 | 0.10 | 0.29 | 1.03E-04 | Operative procedures - OPCS4: Y75.2 Laparoscopic approach to abdominal cavity NEC | Summary Operations |
| 37164/616(37780) | 0.19 | 0.03 | 0.35 | 1.92E-02 | Non-cancer illness code, self-reported: cataract | Medical conditions |
| 37468/316(37784) | 0.19 | -0.03 | 0.42 | 9.32E-02 | Operative procedures - OPCS4: X72.2 Delivery of complex parenteral chemotherapy for neoplasm at first attendance | Summary Operations |
| 37560/224(37784) | 0.19 | -0.07 | 0.46 | 1.58E-01 | Diagnoses - ICD10: R19.5 Other fecal abnormalities | Summary Diagnoses |
| 37042/742(37784) | 0.19 | 0.04 | 0.34 | 1.09E-02 | Diagnoses - ICD10: A09.9 Gastroenteritis and colitis of unspecified origin | Summary Diagnoses |
| 37330/454(37784) | 0.19 | 0.00 | 0.38 | 4.57E-02 | Manic/hyper symptoms: I needed less sleep than usual | Psychosocial factors |
| 37306/288(37594) | 0.19 | -0.04 | 0.43 | 1.12E-01 | Reason for glasses/contact lenses: For a 'squint' or 'turn' in an eye since childhood (called 'strabismus') | Health and medical history |
| 36857/923(37780) | 0.19 | 0.06 | 0.32 | 4.98E-03 | Non-cancer illness code, self-reported: irritable bowel syndrome | Medical conditions |
| 35508/757(36265) | 0.19 | 0.04 | 0.34 | 1.09E-02 | Treatment speciality of consultant (recoded): Neurology | Summary Administration |
| 33118/4544(37662) | 0.19 | 0.12 | 0.26 | 1.56E-07 | Medication for cholesterol, blood pressure or diabetes: Cholesterol lowering medication | Health and medical history |
| 37459/325(37784) | 0.19 | -0.03 | 0.41 | 9.36E-02 | Operative procedures - OPCS4: Z66.5 Lumbar vertebra | Summary Operations |
| 37360/420(37780) | 0.19 | -0.01 | 0.38 | 5.97E-02 | Operation code: mastectomy | Operations |
| 37551/233(37784) | 0.19 | -0.07 | 0.45 | 1.59E-01 | Diagnoses - ICD10: Z09.9 Follow-up examination after unspecified treatment for other conditions | Summary Diagnoses |
| 30554/6158(36712) | 0.19 | 0.13 | 0.24 | 3.33E-11 | Tense / 'highly strung' | Psychosocial factors |
| 37142/568(37710) | 0.19 | 0.02 | 0.36 | 2.91E-02 | Pain type(s) experienced in last month: Pain all over the body | Health and medical history |
| 37420/364(37784) | 0.19 | -0.02 | 0.40 | 8.00E-02 | Diagnoses - ICD10: Z60.2 Living alone | Summary Diagnoses |
| 34797/2807(37604) | 0.19 | 0.11 | 0.26 | 2.41E-06 | Reason for glasses/contact lenses: For long-sightedness, i.e. for distance and near, but particularly for near tasks like reading (called 'hypermetropia') | Health and medical history |
| 35601/2183(37784) | 0.19 | 0.10 | 0.27 | 3.25E-05 | Diagnoses - ICD10: R07.4 Chest pain, unspecified | Summary Diagnoses |
| 37524/260(37784) | 0.19 | -0.06 | 0.43 | 1.39E-01 | Diagnoses - ICD10: Z13.8 Special screening examination for other specified diseases and disorders | Summary Diagnoses |
| 37570/210(37780) | 0.19 | -0.09 | 0.46 | 1.85E-01 | Operation code: gynaecological surgery | Operations |
| 37010/774(37784) | 0.19 | 0.04 | 0.33 | 1.16E-02 | Diagnoses - ICD10: Z53.0 Procedure not carried out because of contraindication | Summary Diagnoses |
| 34705/3079(37784) | 0.19 | 0.11 | 0.26 | 1.18E-06 | Operative procedures - OPCS4: G45.9 Unspecified diagnostic fibreoptic endoscopic examination of upper gastrointestinal tract | Summary Operations |
| 37245/535(37780) | 0.19 | 0.01 | 0.36 | 3.53E-02 | Non-cancer illness code, self-reported: anxiety/panic attacks | Medical conditions |
| 37156/624(37780) | 0.19 | 0.03 | 0.35 | 2.32E-02 | Operation code: shoulder surgery | Operations |
| 37532/252(37784) | 0.19 | -0.07 | 0.44 | 1.49E-01 | Diagnoses - ICD10: I50.0 Congestive heart failure | Summary Diagnoses |
| 36563/1221(37784) | 0.18 | 0.07 | 0.30 | 1.70E-03 | Diagnoses - ICD10: K30 Dyspepsia | Summary Diagnoses |
| 36985/799(37784) | 0.18 | 0.04 | 0.33 | 1.07E-02 | Operative procedures - OPCS4: Y53.1 Approach to organ under radiological control | Summary Operations |
| 34061/3723(37784) | 0.18 | 0.11 | 0.25 | 3.00E-07 | Diagnoses - ICD10: E78.0 Pure hypercholesterolaemia | Summary Diagnoses |
| 36072/1712(37784) | 0.18 | 0.08 | 0.28 | 2.85E-04 | Diagnoses - ICD10: R10.4 Other and unspecified abdominal pain | Summary Diagnoses |
| 36754/1026(37780) | 0.18 | 0.06 | 0.31 | 4.66E-03 | Treatment/medication code: ventolin 100micrograms inhaler | Medications |
| 36035/1607(37642) | 0.18 | 0.08 | 0.28 | 4.87E-04 | Main speciality of consultant (recoded): Geriatric medicine | Summary Administration |
| 37522/262(37784) | 0.18 | -0.06 | 0.43 | 1.50E-01 | Diagnoses - ICD10: J47 Bronchiectasis | Summary Diagnoses |
| 35733/499(36232) | 0.18 | 0.00 | 0.36 | 4.79E-02 | Treatment speciality of consultant (recoded): Upper gastrointestinal surgery | Summary Administration |
| 36567/1131(37698) | 0.18 | 0.06 | 0.30 | 3.26E-03 | Mouth/teeth dental problems: Painful gums | Health and medical history |
| 37394/390(37784) | 0.18 | -0.02 | 0.38 | 8.17E-02 | Diagnoses - ICD10: R07.2 Precordial pain | Summary Diagnoses |
| 36892/747(37639) | 0.18 | 0.03 | 0.33 | 1.66E-02 | Main speciality of consultant (recoded): Neurology | Summary Administration |
| 37396/384(37780) | 0.18 | -0.03 | 0.38 | 8.84E-02 | Treatment/medication code: warfarin | Medications |
| 35590/2190(37780) | 0.18 | 0.09 | 0.26 | 7.74E-05 | Non-cancer illness code, self-reported: depression | Medical conditions |
| 37417/367(37784) | 0.18 | -0.03 | 0.38 | 9.62E-02 | PCT responsible for patient data: WAKEFIELD DISTRICT PCT | Summary Administration |
| 37256/464(37720) | 0.18 | -0.01 | 0.36 | 6.31E-02 | Methods of admission to hospital (recoded): Emergency admission: other | Summary Administration |
| 37572/212(37784) | 0.18 | -0.10 | 0.45 | 2.10E-01 | Diagnoses - ICD10: N73.6 Female pelvic peritoneal adhesions | Summary Diagnoses |
| 37489/295(37784) | 0.18 | -0.06 | 0.41 | 1.39E-01 | Operative procedures - OPCS4: B27.4 Total mastectomy NEC | Summary Operations |
| 36200/1584(37784) | 0.18 | 0.07 | 0.28 | 8.24E-04 | Diagnoses - ICD10: E03.9 Hypothyroidism, unspecified | Summary Diagnoses |
| 37522/258(37780) | 0.18 | -0.07 | 0.42 | 1.66E-01 | Treatment/medication code: zinc product | Medications |
| 36680/348(37028) | 0.17 | -0.04 | 0.39 | 1.09E-01 | Destinations on discharge from hospital (recoded): Temporary place of residence | Summary Administration |
| 37424/294(37718) | 0.17 | -0.07 | 0.42 | 1.60E-01 | Methods of admission to hospital (recoded): Emergency admission: Other injury | Summary Administration |
| 26158/10626(36784) | 0.17 | 0.13 | 0.22 | 2.02E-13 | Treatment speciality of consultant (recoded): General medicine | Summary Administration |
| 36843/876(37719) | 0.17 | 0.04 | 0.31 | 1.27E-02 | Methods of admission to hospital (recoded): Emergency admission: Bed bureau | Summary Administration |
| 36082/1616(37698) | 0.17 | 0.07 | 0.27 | 7.85E-04 | Mouth/teeth dental problems: Toothache | Health and medical history |
| 31328/6186(37514) | 0.17 | 0.12 | 0.23 | 4.19E-09 | Qualifications | Sociodemographics |
| 34573/3211(37784) | 0.17 | 0.10 | 0.25 | 4.09E-06 | Operative procedures - OPCS4: Y98.1 Radiology of one body area (or < 20 minutes) | Summary Operations |
| 33095/4685(37780) | 0.17 | 0.11 | 0.24 | 8.63E-08 | Non-cancer illness code, self-reported: high cholesterol | Medical conditions |
| 37526/258(37784) | 0.17 | -0.08 | 0.42 | 1.74E-01 | Operative procedures - OPCS4: T52.1 Palmar fasciectomy | Summary Operations |
| 35863/1921(37784) | 0.17 | 0.08 | 0.26 | 2.94E-04 | Operative procedures - OPCS4: U21.2 Computed tomography NEC | Summary Operations |
| 11520/8824(20344) | 0.17 | 0.11 | 0.23 | 2.69E-08 | Ever used hormone-replacement therapy (HRT) | Sex-specific factors |
| 37348/432(37780) | 0.17 | -0.02 | 0.36 | 8.04E-02 | Non-cancer illness code, self-reported: psoriasis | Medical conditions |
| 37021/763(37784) | 0.17 | 0.03 | 0.32 | 2.12E-02 | Diagnoses - ICD10: R51 Headache | Summary Diagnoses |
| 37491/293(37784) | 0.17 | -0.06 | 0.40 | 1.51E-01 | Operative procedures - OPCS4: T72.3 Release of constriction of sheath of tendon | Summary Operations |
| 37520/264(37784) | 0.17 | -0.07 | 0.42 | 1.73E-01 | Diagnoses - ICD10: K62.8 Other specified diseases of anus and rectum | Summary Diagnoses |
| 37522/262(37784) | 0.17 | -0.08 | 0.42 | 1.79E-01 | Operative procedures - OPCS4: P23.3 Posterior colporrhaphy NEC | Summary Operations |
| 37103/529(37632) | 0.17 | 0.00 | 0.34 | 5.59E-02 | Main speciality of consultant (recoded): Medical oncology | Summary Administration |
| 36839/945(37784) | 0.17 | 0.04 | 0.30 | 1.13E-02 | Diagnoses - ICD10: K20 Oesophagitis | Summary Diagnoses |
| 31786/5658(37444) | 0.17 | 0.11 | 0.23 | 7.89E-09 | Chest pain or discomfort | Health and medical history |
| 37427/357(37784) | 0.17 | -0.04 | 0.38 | 1.20E-01 | Operative procedures - OPCS4: K45.3 Anastomosis of mammary artery to left anterior descending coronary artery | Summary Operations |
| 36710/1074(37784) | 0.17 | 0.05 | 0.29 | 7.40E-03 | Diagnoses - ICD10: Z51.1 Chemotherapy session for neoplasm | Summary Diagnoses |
| 37059/725(37784) | 0.17 | 0.02 | 0.32 | 2.75E-02 | Diagnoses - ICD10: K57.9 Diverticular disease of intestine, part unspecified, without perforation or abscess | Summary Diagnoses |
| 36927/857(37784) | 0.17 | 0.03 | 0.31 | 1.69E-02 | Illnesses of siblings: Chronic bronchitis/emphysema | Family history |
| 37482/302(37784) | 0.17 | -0.06 | 0.40 | 1.53E-01 | Diagnoses - ICD10: B96.2 Escherichia coli [E. coli] as the cause of diseases classified to other chapters | Summary Diagnoses |
| 37580/200(37780) | 0.17 | -0.11 | 0.45 | 2.44E-01 | Operation code: upper limb surgery | Operations |
| 1290/1631(2921) | 0.17 | 0.02 | 0.32 | 2.85E-02 | Stomach/abdominal pain for 3+ months | Health and medical history |
| 37537/247(37784) | 0.17 | -0.09 | 0.42 | 1.98E-01 | Diagnoses - ICD10: Z98.0 Intestinal bypass and anastomosis status | Summary Diagnoses |
| 36685/1099(37784) | 0.17 | 0.05 | 0.29 | 7.14E-03 | Diagnoses - ICD10: Z51.2 Other chemotherapy | Summary Diagnoses |
| 36084/1700(37784) | 0.17 | 0.07 | 0.26 | 9.37E-04 | Operative procedures - OPCS4: Y97.3 Radiology with post contrast | Summary Operations |
| 27965/9649(37614) | 0.17 | 0.12 | 0.21 | 3.44E-12 | Illness, injury, bereavement, stress in last 2 years: Death of a close relative | Psychosocial factors |
| 35194/2445(37639) | 0.17 | 0.08 | 0.25 | 9.66E-05 | Main speciality of consultant (recoded): Accident and emergency (A&E) | Summary Administration |
| 335/298(633) | 0.16 | -0.16 | 0.49 | 3.13E-01 | Facial pains for 3+ months | Health and medical history |
| 37420/364(37784) | 0.16 | -0.04 | 0.37 | 1.22E-01 | Diagnoses - ICD10: J34.2 Deviated nasal septum | Summary Diagnoses |
| 37542/242(37784) | 0.16 | -0.09 | 0.42 | 2.09E-01 | Operative procedures - OPCS4: H28.9 Unspecified diagnostic endoscopic examination of sigmoid colon using rigid sigmoidoscope | Summary Operations |
| 36461/1323(37784) | 0.16 | 0.05 | 0.28 | 3.85E-03 | Diagnoses - ICD10: Z82.4 Family history of ischaemic heart disease and other diseases of the circulatory system | Summary Diagnoses |
| 35878/1906(37784) | 0.16 | 0.07 | 0.26 | 7.05E-04 | Diagnoses - ICD10: E11.9 Without complications | Summary Diagnoses |
| 37567/217(37784) | 0.16 | -0.11 | 0.43 | 2.38E-01 | Diagnoses - ICD10: M47.82 Other spondylosis (Cervical region) | Summary Diagnoses |
| 37531/253(37784) | 0.16 | -0.09 | 0.41 | 2.02E-01 | Operative procedures - OPCS4: Z66.3 Cervical vertebra | Summary Operations |
| 24517/13052(37569) | 0.16 | 0.12 | 0.21 | 2.95E-13 | Seen doctor (GP) for nerves, anxiety, tension or depression | Psychosocial factors |
| 36182/1598(37780) | 0.16 | 0.06 | 0.26 | 1.79E-03 | Treatment/medication code: omega-3/fish oil supplement | Medications |
| 37459/321(37780) | 0.16 | -0.06 | 0.39 | 1.55E-01 | Treatment/medication code: folic acid product | Medications |
| 36594/1186(37780) | 0.16 | 0.04 | 0.28 | 6.82E-03 | Treatment/medication code: multivitamins | Medications |
| 36789/995(37784) | 0.16 | 0.03 | 0.29 | 1.32E-02 | Diagnoses - ICD10: G56.0 Carpal tunnel syndrome | Summary Diagnoses |
| 37480/304(37784) | 0.16 | -0.07 | 0.39 | 1.67E-01 | Diagnoses - ICD10: D12.3 Transverse colon | Summary Diagnoses |
| 34901/1404(36305) | 0.16 | 0.05 | 0.27 | 3.70E-03 | Treatment speciality of consultant (recoded): Thoracic medicine | Summary Administration |
| 37487/297(37784) | 0.16 | -0.07 | 0.39 | 1.74E-01 | Diagnoses - ICD10: Z90.1 Acquired absence of breast(s) | Summary Diagnoses |
| 37505/279(37784) | 0.16 | -0.08 | 0.40 | 1.87E-01 | Operative procedures - OPCS4: A52.2 Therapeutic sacral epidural injection | Summary Operations |
| 246/500(746) | 0.16 | -0.15 | 0.47 | 3.15E-01 | MCV seropositivity for Merkel Cell Polyomavirus | Blood assays |
| 37557/227(37784) | 0.16 | -0.10 | 0.42 | 2.35E-01 | Diagnoses - ICD10: I44.7 Left bundle-branch block, unspecified | Summary Diagnoses |
| 37546/234(37780) | 0.16 | -0.10 | 0.42 | 2.29E-01 | Operation code: throat/larynx surgery (incl tracheostomy) | Operations |
| 35770/460(36230) | 0.16 | -0.03 | 0.35 | 9.49E-02 | Treatment speciality of consultant (recoded): Cardiothoracic surgery | Summary Administration |
| 37379/401(37780) | 0.16 | -0.04 | 0.36 | 1.20E-01 | Treatment/medication code: allopurinol | Medications |
| 29212/8572(37784) | 0.16 | 0.11 | 0.21 | 6.15E-10 | Diagnoses - ICD10: I10 Essential (primary) hypertension | Summary Diagnoses |
| 37491/293(37784) | 0.16 | -0.07 | 0.39 | 1.81E-01 | Diagnoses - ICD10: C78.7 Secondary malignant neoplasm of liver | Summary Diagnoses |
| 37446/338(37784) | 0.16 | -0.06 | 0.38 | 1.52E-01 | Diagnoses - ICD10: N18.9 Chronic renal failure, unspecified | Summary Diagnoses |
| 37407/377(37784) | 0.16 | -0.05 | 0.36 | 1.30E-01 | Diagnoses - ICD10: D70 Agranulocytosis | Summary Diagnoses |
| 37556/228(37784) | 0.16 | -0.10 | 0.42 | 2.39E-01 | Operative procedures - OPCS4: A57.7 Injection of therapeutic substance around spinal nerve root | Summary Operations |
| 37389/395(37784) | 0.16 | -0.04 | 0.36 | 1.22E-01 | Manic/hyper symptoms: I was more creative or had more ideas than usual | Psychosocial factors |
| 37295/489(37784) | 0.16 | -0.02 | 0.34 | 8.98E-02 | Diagnoses - ICD10: B96.8 Other specified bacterial agents as the cause of diseases classified to other chapters | Summary Diagnoses |
| 35887/1747(37634) | 0.16 | 0.06 | 0.25 | 1.62E-03 | Vitamin and mineral supplements: Vitamin B | Health and medical history |
| 29062/8342(37404) | 0.16 | 0.11 | 0.21 | 6.64E-10 | Medication for pain relief, constipation, heartburn: Paracetamol | Health and medical history |
| 36319/1461(37780) | 0.16 | 0.05 | 0.26 | 4.08E-03 | Non-cancer illness code, self-reported: diabetes | Medical conditions |
| 31075/6705(37780) | 0.16 | 0.10 | 0.21 | 1.26E-08 | Treatment/medication code: paracetamol | Medications |
| 37564/220(37784) | 0.16 | -0.11 | 0.43 | 2.56E-01 | Operative procedures - OPCS4: U08.1 Computed tomography of abdomen NEC | Summary Operations |
| 37294/490(37784) | 0.15 | -0.02 | 0.34 | 9.19E-02 | Diagnoses - ICD10: I95.9 Hypotension, unspecified | Summary Diagnoses |
| 37524/260(37784) | 0.15 | -0.09 | 0.40 | 2.20E-01 | Operative procedures - OPCS4: T86.2 Sampling of axillary lymph nodes | Summary Operations |
| 37129/655(37784) | 0.15 | 0.00 | 0.31 | 5.32E-02 | Diagnoses - ICD10: Z90.7 Acquired absence of genital organ(s) | Summary Diagnoses |
| 24764/12211(36975) | 0.15 | 0.11 | 0.20 | 8.43E-12 | Long-standing illness, disability or infirmity | Health and medical history |
| 37530/254(37784) | 0.15 | -0.09 | 0.40 | 2.24E-01 | Operative procedures - OPCS4: A52.1 Therapeutic lumbar epidural injection | Summary Operations |
| 37559/225(37784) | 0.15 | -0.11 | 0.42 | 2.54E-01 | Operative procedures - OPCS4: V55.3 Greater than two levels of spine | Summary Operations |
| 16395/1572(17967) | 0.15 | 0.05 | 0.26 | 5.08E-03 | Ever had hysterectomy (womb removed) | Sex-specific factors |
| 37490/290(37780) | 0.15 | -0.08 | 0.39 | 2.01E-01 | Methods of self-harm used: Ingesting a medication in excess of the normal dose | Self-harm behaviours |
| 34703/2690(37393) | 0.15 | 0.07 | 0.23 | 1.78E-04 | Medication for pain relief, constipation, heartburn: Omeprazole (e.g. Zanprol) | Health and medical history |
| 34354/3430(37784) | 0.15 | 0.08 | 0.22 | 3.51E-05 | Diagnoses - ICD10: Z86.4 Personal history of psychoactive substance abuse | Summary Diagnoses |
| 37296/338(37634) | 0.15 | -0.06 | 0.37 | 1.70E-01 | Main speciality of consultant (recoded): Infectious diseases | Summary Administration |
| 33822/3787(37609) | 0.15 | 0.08 | 0.22 | 1.23E-05 | Illness, injury, bereavement, stress in last 2 years: Serious illness, injury or assault to yourself | Psychosocial factors |
| 33957/3743(37700) | 0.15 | 0.08 | 0.22 | 4.96E-05 | Medication for cholesterol, blood pressure, diabetes, or take exogenous hormones: Blood pressure medication | Health and medical history |
| 35954/1682(37636) | 0.15 | 0.05 | 0.25 | 2.82E-03 | Sources of admission to hospital (recoded): Transfer to other NHS provider: General ward, young physically disabled, A&E | Summary Administration |
| 37323/457(37780) | 0.15 | -0.04 | 0.34 | 1.13E-01 | Non-cancer illness code, self-reported: diverticular disease/diverticulitis | Medical conditions |
| 37211/573(37784) | 0.15 | -0.02 | 0.32 | 7.79E-02 | Diagnoses - ICD10: E78.5 Hyperlipidaemia, unspecified | Summary Diagnoses |
| 27650/10055(37705) | 0.15 | 0.10 | 0.20 | 2.42E-10 | Main speciality of consultant (recoded): General medicine | Summary Administration |
| 37315/469(37784) | 0.15 | -0.03 | 0.34 | 1.10E-01 | Diagnoses - ICD10: Y83.6 Removal of other organ (partial) (total) | Summary Diagnoses |
| 37002/632(37634) | 0.15 | -0.02 | 0.32 | 8.74E-02 | Methods of discharge from hospital (recoded): Discharged on clinical advice/consent: Transfer within provider | Summary Administration |
| 33939/2412(36351) | 0.15 | 0.06 | 0.23 | 5.40E-04 | Treatment speciality of consultant (recoded): Accident & emergency (A&E) | Summary Administration |
| 35939/1773(37712) | 0.15 | 0.05 | 0.24 | 2.86E-03 | Diabetes diagnosed by doctor | Health and medical history |
| 915/3333(4248) | 0.15 | 0.00 | 0.30 | 5.25E-02 | Hip pain for 3+ months | Health and medical history |
| 37441/343(37784) | 0.15 | -0.07 | 0.36 | 1.78E-01 | PCT responsible for patient data: NORTH STAFFORDSHIRE PCT | Summary Administration |
| 32676/4984(37660) | 0.15 | 0.08 | 0.21 | 1.04E-05 | Main speciality of consultant (recoded): Gynaecology | Summary Administration |
| 30913/6352(37265) | 0.15 | 0.09 | 0.20 | 2.11E-07 | Loneliness, isolation | Psychosocial factors |
| 36153/1631(37784) | 0.15 | 0.04 | 0.25 | 4.66E-03 | Diagnoses - ICD10: K29.7 Gastritis, unspecified | Summary Diagnoses |
| 37555/225(37780) | 0.14 | -0.12 | 0.41 | 2.85E-01 | Non-cancer illness code, self-reported: vaginal prolapse/uterine prolapse | Medical conditions |
| 37566/218(37784) | 0.14 | -0.12 | 0.41 | 2.92E-01 | Operative procedures - OPCS4: Z86.4 Metatarsophalangeal joint of great toe | Summary Operations |
| 36605/788(37393) | 0.14 | 0.00 | 0.29 | 4.78E-02 | Medication for pain relief, constipation, heartburn: Ranitidine (e.g. Zantac) | Health and medical history |
| 37489/295(37784) | 0.14 | -0.09 | 0.38 | 2.24E-01 | Diagnoses - ICD10: R05 Cough | Summary Diagnoses |
| 37018/692(37710) | 0.14 | -0.01 | 0.30 | 6.45E-02 | Pain type(s) experienced in last month: Facial pain | Health and medical history |
| 37407/377(37784) | 0.14 | -0.06 | 0.35 | 1.76E-01 | PCT responsible for patient data: NORTH TYNESIDE PCT | Summary Administration |
| 35561/672(36233) | 0.14 | -0.01 | 0.30 | 7.02E-02 | Treatment speciality of consultant (recoded): Medical oncology | Summary Administration |
| 37480/304(37784) | 0.14 | -0.09 | 0.37 | 2.21E-01 | Diagnoses - ICD10: R53 Malaise and fatigue | Summary Diagnoses |
| 37032/745(37777) | 0.14 | 0.00 | 0.29 | 5.78E-02 | Activities undertaken to treat anxiety: Other therapeutic activities such as mindfulness, yoga or art classes | Anxiety |
| 37578/206(37784) | 0.14 | -0.13 | 0.42 | 3.12E-01 | Operative procedures - OPCS4: Z75.3 Wing of ilium | Summary Operations |
| 12292/1326(13618) | 0.14 | 0.03 | 0.26 | 1.56E-02 | Shortness of breath walking on level ground | Health and medical history |
| 29635/7549(37184) | 0.14 | 0.09 | 0.19 | 5.46E-08 | Wheeze or whistling in the chest in last year | Health and medical history |
| 36946/838(37784) | 0.14 | 0.00 | 0.28 | 4.82E-02 | Operative procedures - OPCS4: Q07.4 Total abdominal hysterectomy NEC | Summary Operations |
| 37541/239(37780) | 0.14 | -0.11 | 0.40 | 2.81E-01 | Operation code: anal surgery | Operations |
| 37559/219(37778) | 0.14 | -0.13 | 0.41 | 3.04E-01 | Actions taken following self-harm: Need hospital treatment (eg A&E) | Self-harm behaviours |
| 37332/448(37780) | 0.14 | -0.05 | 0.33 | 1.42E-01 | Non-cancer illness code, self-reported: glaucoma | Medical conditions |
| 37326/454(37780) | 0.14 | -0.05 | 0.33 | 1.39E-01 | Operation code: spine or back surgery | Operations |
| 31890/5717(37607) | 0.14 | 0.08 | 0.20 | 1.23E-06 | Reason for glasses/contact lenses: For just reading/near work as you are getting older (called 'presbyopia') | Health and medical history |
| 37166/614(37780) | 0.14 | -0.02 | 0.30 | 8.85E-02 | Non-cancer illness code, self-reported: cholelithiasis/gall stones | Medical conditions |
| 37204/580(37784) | 0.14 | -0.03 | 0.31 | 9.79E-02 | Operative procedures - OPCS4: Z92.4 Chest NEC | Summary Operations |
| 36591/1193(37784) | 0.14 | 0.02 | 0.26 | 1.85E-02 | Operative procedures - OPCS4: Y82.9 Unspecified local anaesthetic | Summary Operations |
| 37013/771(37784) | 0.14 | 0.00 | 0.29 | 5.85E-02 | Diagnoses - ICD10: R79.8 Other specified abnormal findings of blood chemistry | Summary Diagnoses |
| 34548/3164(37712) | 0.14 | 0.07 | 0.21 | 2.08E-04 | Pain type(s) experienced in last month: Stomach or abdominal pain | Health and medical history |
| 36763/1021(37784) | 0.14 | 0.01 | 0.27 | 3.00E-02 | Diagnoses - ICD10: R55 Syncope and collapse | Summary Diagnoses |
| 37133/651(37784) | 0.14 | -0.02 | 0.30 | 8.05E-02 | Diagnoses - ICD10: Z88.8 Personal history of allergy to other drugs, medicaments and biological substances | Summary Diagnoses |
| 35901/1879(37780) | 0.14 | 0.04 | 0.23 | 4.15E-03 | Non-cancer illness code, self-reported: hypothyroidism/myxoedema | Medical conditions |
| 37237/547(37784) | 0.14 | -0.03 | 0.31 | 1.14E-01 | Diagnoses - ICD10: N39.3 Stress incontinence | Summary Diagnoses |
| 35716/602(36318) | 0.14 | -0.02 | 0.30 | 9.54E-02 | Treatment speciality of consultant (recoded): Pain management | Summary Administration |
| 37364/416(37780) | 0.14 | -0.06 | 0.33 | 1.66E-01 | Fractured bone site(s): Arm | Health and medical history |
| 37498/282(37780) | 0.14 | -0.10 | 0.38 | 2.52E-01 | Non-cancer illness code, self-reported: headaches (not migraine) | Medical conditions |
| 32976/4734(37710) | 0.14 | 0.08 | 0.20 | 1.32E-05 | Pain type(s) experienced in last month: Hip pain | Health and medical history |
| 37199/585(37784) | 0.14 | -0.03 | 0.30 | 1.04E-01 | Diagnoses - ICD10: F41.9 Anxiety disorder, unspecified | Summary Diagnoses |
| 34357/3427(37784) | 0.14 | 0.07 | 0.21 | 1.65E-04 | Operative procedures - OPCS4: Z27.4 Duodenum | Summary Operations |
| 10793/2935(13728) | 0.14 | 0.05 | 0.22 | 1.29E-03 | Leg pain on walking | Health and medical history |
| 428/19932(20360) | 0.14 | -0.06 | 0.33 | 1.68E-01 | Ever had cervical smear test | Sex-specific factors |
| 37560/220(37780) | 0.14 | -0.13 | 0.41 | 3.21E-01 | Treatment/medication code: codeine | Medications |
| 36588/1196(37784) | 0.14 | 0.02 | 0.25 | 2.23E-02 | Operative procedures - OPCS4: Y98.2 Radiology of two body areas | Summary Operations |
| 37486/298(37784) | 0.14 | -0.10 | 0.37 | 2.52E-01 | Diagnoses - ICD10: F10.1 Harmful use | Summary Diagnoses |
| 37163/466(37629) | 0.14 | -0.05 | 0.32 | 1.51E-01 | Main speciality of consultant (recoded): Endocrinology | Summary Administration |
| 27182/10528(37710) | 0.13 | 0.09 | 0.18 | 1.14E-08 | Vascular/heart problems diagnosed by doctor: High blood pressure | Health and medical history |
| 32196/5499(37695) | 0.13 | 0.08 | 0.19 | 5.27E-06 | Other eye problems | Health and medical history |
| 37427/353(37780) | 0.13 | -0.08 | 0.35 | 2.13E-01 | Non-cancer illness code, self-reported: appendicitis | Medical conditions |
| 37147/637(37784) | 0.13 | -0.02 | 0.29 | 9.82E-02 | Diagnoses - ICD10: Z95.5 Presence of coronary angioplasty implant and graft | Summary Diagnoses |
| 37504/280(37784) | 0.13 | -0.10 | 0.37 | 2.69E-01 | Diagnoses - ICD10: I63.9 Cerebral infarction, unspecified | Summary Diagnoses |
| 35978/1722(37700) | 0.13 | 0.03 | 0.23 | 8.58E-03 | Medication for cholesterol, blood pressure, diabetes, or take exogenous hormones: Hormone replacement therapy | Health and medical history |
| 37384/396(37780) | 0.13 | -0.07 | 0.33 | 1.89E-01 | Treatment/medication code: candesartan cilexetil | Medications |
| 37332/452(37784) | 0.13 | -0.05 | 0.32 | 1.62E-01 | Operative procedures - OPCS4: X72.1 Delivery of complex chemotherapy for neoplasm including prolonged infusional treatment at first attendance | Summary Operations |
| 32465/5145(37610) | 0.13 | 0.07 | 0.19 | 1.25E-05 | Illness, injury, bereavement, stress in last 2 years: Serious illness, injury or assault of a close relative | Psychosocial factors |
| 36297/1487(37784) | 0.13 | 0.03 | 0.24 | 1.28E-02 | Diagnoses - ICD10: R69 Unknown and unspecified causes of morbidity | Summary Diagnoses |
| 32987/4671(37658) | 0.13 | 0.06 | 0.20 | 2.02E-04 | Medication for cholesterol, blood pressure or diabetes: Blood pressure medication | Health and medical history |
| 37173/611(37784) | 0.13 | -0.03 | 0.29 | 1.10E-01 | Diagnoses - ICD10: Y83.8 Other surgical procedures | Summary Diagnoses |
| 36894/890(37784) | 0.13 | 0.00 | 0.27 | 5.48E-02 | Operative procedures - OPCS4: Z84.6 Knee joint | Summary Operations |
| 37447/337(37784) | 0.13 | -0.09 | 0.35 | 2.35E-01 | Diagnoses - ICD10: I84.8 Unspecified haemorrhoids with other complications | Summary Diagnoses |
| 37435/345(37780) | 0.13 | -0.08 | 0.35 | 2.32E-01 | Operation code: male circumcision | Operations |
| 24293/13193(37486) | 0.13 | 0.09 | 0.17 | 2.50E-09 | Eye problems/disorders | Health and medical history |
| 37084/700(37784) | 0.13 | -0.02 | 0.28 | 8.99E-02 | Diagnoses - ICD10: N32.8 Other specified disorders of bladder | Summary Diagnoses |
| 27880/9900(37780) | 0.13 | 0.08 | 0.18 | 6.13E-08 | Non-cancer illness code, self-reported: hypertension | Medical conditions |
| 36792/927(37719) | 0.13 | 0.00 | 0.26 | 5.30E-02 | Methods of admission to hospital (recoded): Transfer | Summary Administration |
| 37191/593(37784) | 0.13 | -0.03 | 0.30 | 1.21E-01 | Operative procedures - OPCS4: Y82.3 Application of local anaesthetic NEC | Summary Operations |
| 37568/216(37784) | 0.13 | -0.14 | 0.40 | 3.45E-01 | Operative procedures - OPCS4: D02.1 Excision of lesion of external ear | Summary Operations |
| 34604/3180(37784) | 0.13 | 0.06 | 0.20 | 5.69E-04 | Illnesses of siblings: Heart disease | Family history |
| 34791/1477(36268) | 0.13 | 0.02 | 0.23 | 1.58E-02 | Treatment speciality of consultant (recoded): Oral surgery | Summary Administration |
| 37565/219(37784) | 0.13 | -0.14 | 0.40 | 3.47E-01 | Diagnoses - ICD10: R56.8 Other and unspecified convulsions | Summary Diagnoses |
| 36397/1383(37780) | 0.13 | 0.02 | 0.24 | 2.05E-02 | Treatment/medication code: lansoprazole | Medications |
| 37470/314(37784) | 0.13 | -0.09 | 0.35 | 2.60E-01 | PCT responsible for patient data: BIRMINGHAM EAST AND NORTH PCT | Summary Administration |
| 36661/1119(37780) | 0.13 | 0.01 | 0.25 | 3.66E-02 | Treatment/medication code: lisinopril | Medications |
| 37172/608(37780) | 0.13 | -0.03 | 0.29 | 1.22E-01 | Non-cancer illness code, self-reported: uterine fibroids | Medical conditions |
| 37305/479(37784) | 0.13 | -0.05 | 0.31 | 1.68E-01 | Diagnoses - ICD10: J18.9 Pneumonia, unspecified | Summary Diagnoses |
| 35375/880(36255) | 0.13 | -0.01 | 0.26 | 6.33E-02 | Treatment speciality of consultant (recoded): Neurosurgery | Summary Administration |
| 31158/6626(37784) | 0.13 | 0.07 | 0.18 | 3.00E-06 | Illnesses of siblings: High blood pressure | Family history |
| 37481/303(37784) | 0.13 | -0.10 | 0.36 | 2.73E-01 | Manic/hyper symptoms: All of the above | Psychosocial factors |
| 37553/231(37784) | 0.13 | -0.13 | 0.39 | 3.39E-01 | Operative procedures - OPCS4: Y02.2 Insertion of prosthesis into organ NOC | Summary Operations |
| 37025/759(37784) | 0.13 | -0.02 | 0.27 | 8.56E-02 | PCT responsible for patient data: DERBYSHIRE COUNTY PCT | Summary Administration |
| 36997/783(37780) | 0.13 | -0.02 | 0.27 | 8.07E-02 | Operation code: colonoscopy/sigmoidoscopy | Operations |
| 37017/767(37784) | 0.13 | -0.02 | 0.27 | 8.52E-02 | Diagnoses - ICD10: J18.1 Lobar pneumonia, unspecified | Summary Diagnoses |
| 36425/1359(37784) | 0.13 | 0.01 | 0.24 | 2.81E-02 | Diagnoses - ICD10: N40 Hyperplasia of prostate | Summary Diagnoses |
| 1230/1638(2868) | 0.13 | -0.02 | 0.28 | 9.75E-02 | Leg pain when walking normally | Health and medical history |
| 36188/1592(37780) | 0.13 | 0.03 | 0.23 | 1.44E-02 | Non-cancer illness code, self-reported: gastro-oesophageal reflux (gord) / gastric reflux | Medical conditions |
| 36685/1036(37721) | 0.13 | 0.00 | 0.25 | 4.68E-02 | Mineral and other dietary supplements: Selenium | Health and medical history |
| 37490/294(37784) | 0.13 | -0.11 | 0.36 | 2.87E-01 | Diagnoses - ICD10: Z95.0 Presence of cardiac pacemaker | Summary Diagnoses |
| 36449/1335(37784) | 0.13 | 0.02 | 0.24 | 2.52E-02 | Operative procedures - OPCS4: Z92.6 Abdomen NEC | Summary Operations |
| 36189/1591(37780) | 0.13 | 0.02 | 0.23 | 1.60E-02 | Treatment/medication code: amlodipine | Medications |
| 36507/1100(37607) | 0.13 | 0.00 | 0.25 | 4.39E-02 | Illness, injury, bereavement, stress in last 2 years: Marital separation/divorce | Psychosocial factors |
| 36877/907(37784) | 0.13 | -0.01 | 0.26 | 6.80E-02 | Operative procedures - OPCS4: Q22.1 Bilateral salpingoophorectomy | Summary Operations |
| 36221/1170(37391) | 0.13 | 0.01 | 0.24 | 3.76E-02 | Medication for pain relief, constipation, heartburn: Laxatives (e.g. Dulcolax, Senokot) | Health and medical history |
| 1031/1802(2833) | 0.12 | -0.03 | 0.28 | 1.15E-01 | Leg pain when standing still or sitting | Health and medical history |
| 33388/4337(37725) | 0.12 | 0.06 | 0.19 | 1.27E-04 | Blood clot, DVT, bronchitis, emphysema, asthma, rhinitis, eczema, allergy diagnosed by doctor: Asthma | Health and medical history |
| 37281/499(37780) | 0.12 | -0.05 | 0.30 | 1.70E-01 | Treatment/medication code: garlic product | Medications |
| 37323/461(37784) | 0.12 | -0.06 | 0.31 | 1.89E-01 | Operative procedures - OPCS4: U35.4 Computed tomography of pulmonary arteries | Summary Operations |
| 348/398(746) | 0.12 | -0.17 | 0.42 | 4.08E-01 | JCV seropositivity for Human Polyomavirus JCV | Blood assays |
| 37378/406(37784) | 0.12 | -0.07 | 0.32 | 2.20E-01 | Diagnoses - ICD10: I50.1 Left ventricular failure | Summary Diagnoses |
| 33689/3919(37608) | 0.12 | 0.06 | 0.19 | 4.07E-04 | Illness, injury, bereavement, stress in last 2 years: Financial difficulties | Psychosocial factors |
| 37238/546(37784) | 0.12 | -0.05 | 0.29 | 1.56E-01 | Diagnoses - ICD10: T81.0 Haemorrhage and haematoma complicating a procedure, not elsewhere classified | Summary Diagnoses |
| 19800/17904(37704) | 0.12 | 0.08 | 0.16 | 6.65E-09 | Taking other prescription medications | Health and medical history |
| 33527/4253(37780) | 0.12 | 0.06 | 0.19 | 1.80E-04 | Non-cancer illness code, self-reported: asthma | Medical conditions |
| 37383/401(37784) | 0.12 | -0.08 | 0.33 | 2.31E-01 | PCT responsible for patient data: NEWCASTLE PCT | Summary Administration |
| 36565/1219(37784) | 0.12 | 0.01 | 0.24 | 3.64E-02 | Operative procedures - OPCS4: Z28.5 Descending colon | Summary Operations |
| 36730/1054(37784) | 0.12 | 0.00 | 0.25 | 5.20E-02 | Operative procedures - OPCS4: W90.3 Injection of therapeutic substance into joint | Summary Operations |
| 37511/273(37784) | 0.12 | -0.12 | 0.36 | 3.17E-01 | Diagnoses - ICD10: I84.1 Internal haemorrhoids with other complications | Summary Diagnoses |
| 35573/2207(37780) | 0.12 | 0.04 | 0.21 | 5.88E-03 | Treatment/medication code: omeprazole | Medications |
| 20779/16132(36911) | 0.12 | 0.08 | 0.16 | 9.49E-09 | Mood swings | Psychosocial factors |
| 33431/4231(37662) | 0.12 | 0.06 | 0.19 | 2.10E-04 | Seen a psychiatrist for nerves, anxiety, tension or depression | Psychosocial factors |
| 36806/978(37784) | 0.12 | -0.01 | 0.25 | 6.26E-02 | Operative procedures - OPCS4: A65.1 Carpal tunnel release | Summary Operations |
| 37491/289(37780) | 0.12 | -0.11 | 0.36 | 3.06E-01 | Operation code: maxillo-facial surgery | Operations |
| 37195/445(37640) | 0.12 | -0.07 | 0.31 | 2.07E-01 | Methods of discharge from hospital (recoded): Discharged without clinical advice/consent: Self, relative or advocate | Summary Administration |
| 36005/1779(37784) | 0.12 | 0.03 | 0.22 | 1.34E-02 | Diagnoses - ICD10: K21.9 Gastro-oesophageal reflux disease without oesophagitis | Summary Diagnoses |
| 33865/3915(37780) | 0.12 | 0.05 | 0.19 | 9.07E-04 | Operation code: hysterectomy | Operations |
| 37266/518(37784) | 0.12 | -0.05 | 0.30 | 1.77E-01 | Operative procedures - OPCS4: Y98.3 Radiology of three body areas (or 20-40 minutes) | Summary Operations |
| 37458/326(37784) | 0.12 | -0.10 | 0.34 | 2.85E-01 | Diagnoses - ICD10: K66.0 Peritoneal adhesions | Summary Diagnoses |
| 37282/351(37633) | 0.12 | -0.09 | 0.33 | 2.65E-01 | Main speciality of consultant (recoded): Radiology | Summary Administration |
| 37356/424(37780) | 0.12 | -0.07 | 0.31 | 2.24E-01 | Treatment/medication code: seretide 50 evohaler | Medications |
| 36481/1303(37784) | 0.12 | 0.01 | 0.23 | 3.54E-02 | Operative procedures - OPCS4: J18.3 Total cholecystectomy NEC | Summary Operations |
| 37391/393(37784) | 0.12 | -0.08 | 0.32 | 2.42E-01 | Diagnoses - ICD10: K42.9 Umbilical hernia without obstruction or gangrene | Summary Diagnoses |
| 37491/293(37784) | 0.12 | -0.11 | 0.35 | 3.12E-01 | Diagnoses - ICD10: J98.1 Pulmonary collapse | Summary Diagnoses |
| 36162/1618(37780) | 0.12 | 0.02 | 0.22 | 2.29E-02 | Operation code: bilateral oophorectomy | Operations |
| 37339/445(37784) | 0.12 | -0.07 | 0.31 | 2.14E-01 | PCT responsible for patient data: SURREY PCT | Summary Administration |
| 36019/1765(37784) | 0.12 | 0.02 | 0.22 | 1.69E-02 | Diagnoses - ICD10: I48 Atrial fibrillation and flutter | Summary Diagnoses |
| 37046/738(37784) | 0.12 | -0.03 | 0.27 | 1.13E-01 | Diagnoses - ICD10: Z96.1 Presence of intraocular lens | Summary Diagnoses |
| 37203/581(37784) | 0.12 | -0.05 | 0.29 | 1.58E-01 | Diagnoses - ICD10: L03.1 Cellulitis of other parts of limb | Summary Diagnoses |
| 37547/237(37784) | 0.12 | -0.14 | 0.38 | 3.65E-01 | Diagnoses - ICD10: Z86.1 Personal history of infectious and parasitic diseases | Summary Diagnoses |
| 37551/233(37784) | 0.12 | -0.14 | 0.38 | 3.69E-01 | Operative procedures - OPCS4: Z40.1 Pulmonary artery | Summary Operations |
| 36092/1607(37699) | 0.12 | 0.02 | 0.22 | 2.11E-02 | Mouth/teeth dental problems: Loose teeth | Health and medical history |
| 37531/253(37784) | 0.12 | -0.13 | 0.37 | 3.53E-01 | Operative procedures - OPCS4: M45.1 Diagnostic endoscopic examination of bladder and biopsy of lesion of bladder NEC | Summary Operations |
| 28690/9024(37714) | 0.12 | 0.07 | 0.17 | 1.24E-06 | Pain type(s) experienced in last month: Neck or shoulder pain | Health and medical history |
| 37356/428(37784) | 0.12 | -0.07 | 0.31 | 2.31E-01 | Operative procedures - OPCS4: X72.3 Delivery of simple parenteral chemotherapy for neoplasm at first attendance | Summary Operations |
| 37000/784(37784) | 0.12 | -0.03 | 0.26 | 1.13E-01 | Diagnoses - ICD10: Z85.3 Personal history of malignant neoplasm of breast | Summary Diagnoses |
| 36223/1557(37780) | 0.12 | 0.01 | 0.22 | 2.70E-02 | Treatment/medication code: levothyroxine sodium | Medications |
| 37338/291(37629) | 0.12 | -0.12 | 0.35 | 3.26E-01 | Main speciality of consultant (recoded): Oral and maxillo facial surgery | Summary Administration |
| 27696/10017(37713) | 0.12 | 0.07 | 0.16 | 7.72E-07 | Pain type(s) experienced in last month: Back pain | Health and medical history |
| 36431/1353(37784) | 0.12 | -0.01 | 0.24 | 6.08E-02 | Patient classification on admission (recoded): Day case: In day bed unit | Summary Administration |
| 37293/339(37632) | 0.12 | -0.10 | 0.33 | 2.92E-01 | Main speciality of consultant (recoded): Nephrology | Summary Administration |
| 33931/3724(37655) | 0.11 | 0.05 | 0.18 | 1.18E-03 | Main speciality of consultant (recoded): Cardiology | Summary Administration |
| 37488/296(37784) | 0.11 | -0.12 | 0.35 | 3.32E-01 | Type of cancer: ICD10: C50.4 Upper-outer quadrant of breast | Cancer register |
| 37430/350(37780) | 0.11 | -0.10 | 0.33 | 2.93E-01 | Non-cancer illness code, self-reported: spine arthritis/spondylitis | Medical conditions |
| 37469/315(37784) | 0.11 | -0.11 | 0.34 | 3.18E-01 | Operative procedures - OPCS4: L91.2 Insertion of central venous catheter NEC | Summary Operations |
| 36733/1051(37784) | 0.11 | -0.01 | 0.24 | 7.15E-02 | Operative procedures - OPCS4: O16.1 Pelvis NEC | Summary Operations |
| 36641/1143(37784) | 0.11 | -0.01 | 0.23 | 6.09E-02 | Operative procedures - OPCS4: Z27.6 Ileum | Summary Operations |
| 36906/878(37784) | 0.11 | -0.02 | 0.25 | 1.07E-01 | Diagnoses - ICD10: J44.9 Chronic obstructive pulmonary disease, unspecified | Summary Diagnoses |
| 37429/355(37784) | 0.11 | -0.10 | 0.33 | 3.03E-01 | Operative procedures - OPCS4: T43.9 Unspecified diagnostic endoscopic examination of peritoneum | Summary Operations |
| 37583/201(37784) | 0.11 | -0.17 | 0.39 | 4.31E-01 | Operative procedures - OPCS4: Z67.6 Lumbosacral joint | Summary Operations |
| 37346/438(37784) | 0.11 | -0.08 | 0.30 | 2.47E-01 | Operative procedures - OPCS4: Z48.2 Skin of neck | Summary Operations |
| 33462/4238(37700) | 0.11 | 0.05 | 0.18 | 6.57E-04 | Mouth/teeth dental problems: Mouth ulcers | Health and medical history |
| 36886/898(37784) | 0.11 | -0.02 | 0.25 | 1.02E-01 | Diagnoses - ICD10: K80.2 Calculus of gallbladder without cholecystitis | Summary Diagnoses |
| 36189/1595(37784) | 0.11 | 0.01 | 0.21 | 3.05E-02 | Diagnoses - ICD10: Z53.8 Procedure not carried out for other reasons | Summary Diagnoses |
| 37426/354(37780) | 0.11 | -0.10 | 0.32 | 3.00E-01 | Operation code: spinal laminectomy | Operations |
| 37485/299(37784) | 0.11 | -0.12 | 0.34 | 3.43E-01 | Diagnoses - ICD10: R91 Abnormal findings on diagnostic imaging of lung | Summary Diagnoses |
| 35993/234(36227) | 0.11 | -0.15 | 0.37 | 4.01E-01 | Treatment speciality of consultant (recoded): Nephrology | Summary Administration |
| 22697/1233(23930) | 0.11 | -0.01 | 0.23 | 6.26E-02 | Hearing aid user | Health and medical history |
| 36617/819(37436) | 0.11 | -0.03 | 0.25 | 1.21E-01 | Eye problems/disorders: Other serious eye condition | Health and medical history |
| 18398/1678(20076) | 0.11 | 0.01 | 0.21 | 3.34E-02 | Bilateral oophorectomy (both ovaries removed) | Sex-specific factors |
| 36496/1288(37784) | 0.11 | 0.00 | 0.22 | 5.57E-02 | Diagnoses - ICD10: K59.0 Constipation | Summary Diagnoses |
| 37569/215(37784) | 0.11 | -0.16 | 0.38 | 4.26E-01 | Operative procedures - OPCS4: Y13.1 Cauterisation of lesion of organ NOC | Summary Operations |
| 36273/1511(37784) | 0.11 | 0.01 | 0.21 | 3.88E-02 | Diagnoses - ICD10: K52.9 Non-infective gastro-enteritis and colitis, unspecified | Summary Diagnoses |
| 28916/8797(37713) | 0.11 | 0.06 | 0.16 | 8.67E-06 | Pain type(s) experienced in last month: Knee pain | Health and medical history |
| 36252/1528(37780) | 0.11 | 0.01 | 0.21 | 3.93E-02 | Non-cancer illness code, self-reported: unclassifiable | Medical conditions |
| 30525/6985(37510) | 0.11 | 0.06 | 0.16 | 6.28E-05 | Qualifications: NVQ or HND or HNC or equivalent | Sociodemographics |
| 37479/305(37784) | 0.11 | -0.12 | 0.34 | 3.48E-01 | Operative procedures - OPCS4: X70.2 Procurement of drugs for chemotherapy for neoplasm for regimens in Band 2 | Summary Operations |
| 37486/294(37780) | 0.11 | -0.12 | 0.34 | 3.61E-01 | Non-cancer illness code, self-reported: endometriosis | Medical conditions |
| 37075/705(37780) | 0.11 | -0.04 | 0.26 | 1.60E-01 | Treatment/medication code: amitriptyline | Medications |
| 37543/241(37784) | 0.11 | -0.15 | 0.36 | 4.08E-01 | Diagnoses - ICD10: M51.2 Other specified intervertebral disk displacement | Summary Diagnoses |
| 37313/471(37784) | 0.11 | -0.08 | 0.29 | 2.50E-01 | Diagnoses - ICD10: M15.9 Polyarthrosis, unspecified | Summary Diagnoses |
| 34878/2906(37784) | 0.11 | 0.03 | 0.18 | 5.83E-03 | Illnesses of father: Lung cancer | Family history |
| 36884/896(37780) | 0.11 | -0.03 | 0.24 | 1.18E-01 | Treatment/medication code: metformin | Medications |
| 37482/302(37784) | 0.11 | -0.12 | 0.34 | 3.56E-01 | Diagnoses - ICD10: M23.22 Derangement of meniscus due to old tear or injury (Posterior cruciate ligament or Posterior horn of medial meniscus) | Summary Diagnoses |
| 37417/363(37780) | 0.11 | -0.10 | 0.32 | 3.16E-01 | Operation code: breast cyst/abscess removal | Operations |
| 36617/1167(37784) | 0.11 | -0.01 | 0.23 | 7.76E-02 | Operative procedures - OPCS4: K63.4 Coronary arteriography using two catheters | Summary Operations |
| 545/201(746) | 0.11 | -0.23 | 0.44 | 5.29E-01 | T. gondii seropositivity for Toxoplasma gondii | Blood assays |
| 28799/7906(36705) | 0.11 | 0.06 | 0.16 | 3.52E-05 | Treatment speciality of consultant (recoded): Gastroenterology | Summary Administration |
| 37050/734(37784) | 0.11 | -0.04 | 0.25 | 1.58E-01 | Diagnoses - ICD10: R10.3 Pain localised to other parts of lower abdomen | Summary Diagnoses |
| 37465/315(37780) | 0.11 | -0.12 | 0.33 | 3.52E-01 | Treatment/medication code: losartan | Medications |
| 37278/506(37784) | 0.11 | -0.07 | 0.28 | 2.43E-01 | Diagnoses - ICD10: M17.1 Other primary gonarthrosis | Summary Diagnoses |
| 923/1916(2839) | 0.11 | -0.06 | 0.27 | 1.97E-01 | Leg pain when walking uphill or hurrying | Health and medical history |
| 7111/30629(37740) | 0.11 | 0.05 | 0.16 | 8.29E-05 | Destinations on discharge from hospital (recoded): Usual Place of residence | Summary Administration |
| 37571/209(37780) | 0.11 | -0.17 | 0.38 | 4.52E-01 | Treatment/medication code: gaviscon liquid | Medications |
| 22543/14574(37117) | 0.11 | 0.06 | 0.15 | 1.30E-06 | Fed-up feelings | Psychosocial factors |
| 13782/6340(20122) | 0.10 | 0.04 | 0.17 | 6.18E-04 | Ever had stillbirth, spontaneous miscarriage or termination | Sex-specific factors |
| 1666/6417(8083) | 0.10 | 0.00 | 0.21 | 6.03E-02 | Knee pain for 3+ months | Health and medical history |
| 29241/7930(37171) | 0.10 | 0.05 | 0.16 | 4.41E-05 | Other serious medical condition/disability diagnosed by doctor | Health and medical history |
| 37255/525(37780) | 0.10 | -0.07 | 0.28 | 2.38E-01 | Treatment/medication code: perindopril | Medications |
| 37513/271(37784) | 0.10 | -0.14 | 0.35 | 3.95E-01 | Diagnoses - ICD10: M06.9 Rheumatoid arthritis, unspecified | Summary Diagnoses |
| 36947/833(37780) | 0.10 | -0.03 | 0.24 | 1.40E-01 | Fractured bone site(s): Wrist | Health and medical history |
| 33430/4350(37780) | 0.10 | 0.04 | 0.17 | 1.74E-03 | Treatment/medication code: simvastatin | Medications |
| 32665/5115(37780) | 0.10 | 0.04 | 0.17 | 7.92E-04 | Treatment/medication code: aspirin | Medications |
| 37581/203(37784) | 0.10 | -0.17 | 0.38 | 4.62E-01 | Diagnoses - ICD10: W01.0 Home | Summary Diagnoses |
| 746 | 0.10 | -0.04 | 0.25 | 1.60E-01 | HBe antigen for Hepatitis B Virus | Blood assays |
| 30389/7323(37712) | 0.10 | 0.05 | 0.16 | 9.48E-05 | Pain type(s) experienced in last month: Headache | Health and medical history |
| 34784/2940(37724) | 0.10 | 0.03 | 0.18 | 7.96E-03 | Methods of admission to hospital (recoded): Emergency admission | Summary Administration |
| 35805/1791(37596) | 0.10 | 0.01 | 0.20 | 3.38E-02 | Reason for glasses/contact lenses: For 'astigmatism' | Health and medical history |
| 26751/11000(37751) | 0.10 | 0.06 | 0.15 | 7.90E-06 | Methods of admission to hospital (recoded): Emergency admission: A&E | Summary Administration |
| 35827/1957(37784) | 0.10 | 0.01 | 0.20 | 3.08E-02 | Diagnoses - ICD10: I25.1 Atherosclerotic heart disease | Summary Diagnoses |
| 36843/937(37780) | 0.10 | -0.03 | 0.24 | 1.30E-01 | Cancer code, self-reported: breast cancer | Medical conditions |
| 36730/897(37627) | 0.10 | -0.03 | 0.24 | 1.33E-01 | Main speciality of consultant (recoded): Clinical oncology/ Radiotherapy | Summary Administration |
| 37298/486(37784) | 0.10 | -0.08 | 0.28 | 2.70E-01 | Operative procedures - OPCS4: K75.1 Percutaneous transluminal balloon angioplasty and insertion of 1-2 drug-eluting stents into coronary artery | Summary Operations |
| 37397/387(37784) | 0.10 | -0.10 | 0.30 | 3.24E-01 | Diagnoses - ICD10: D12.8 Rectum | Summary Diagnoses |
| 37554/230(37784) | 0.10 | -0.16 | 0.36 | 4.46E-01 | Operative procedures - OPCS4: W15.3 Osteotomy of first metatarsal bone NEC | Summary Operations |
| 37532/252(37784) | 0.10 | -0.15 | 0.35 | 4.27E-01 | Operative procedures - OPCS4: E36.9 Unspecified diagnostic endoscopic examination of larynx | Summary Operations |
| 746 | 0.10 | -0.04 | 0.25 | 1.70E-01 | L1 antigen for Human Papillomavirus type-18 | Blood assays |
| 34772/3012(37784) | 0.10 | 0.03 | 0.18 | 8.75E-03 | Operative procedures - OPCS4: M45.9 Unspecified diagnostic endoscopic examination of bladder | Summary Operations |
| 15792/21063(36855) | 0.10 | 0.06 | 0.14 | 2.75E-06 | Worrier / anxious feelings | Psychosocial factors |
| 36827/957(37784) | 0.10 | -0.03 | 0.23 | 1.31E-01 | Diagnoses - ICD10: Z72.0 Tobacco use | Summary Diagnoses |
| 36865/801(37666) | 0.10 | -0.04 | 0.24 | 1.64E-01 | Main speciality of consultant (recoded): Anaesthetics | Summary Administration |
| 36785/999(37784) | 0.10 | -0.03 | 0.23 | 1.21E-01 | Operative procedures - OPCS4: H25.1 Diagnostic endoscopic examination of lower bowel and biopsy of lesion of lower bowel using fibreoptic sigmoidoscope | Summary Operations |

**Table S5-D. PheWAS results, Beta <= -0.1 using PRS calculated from Jansen et *al*. excluding *APOE* locus.**

| **n** | **Beta** | **Lower CI 95** | **Upper CI 95** | ***p*-value** | **Trait/Description** | **Category** |
| --- | --- | --- | --- | --- | --- | --- |
| **29762/7587(37349)** | **-0.52** | **-0.57** | **-0.46** | **4.24E-66** | **Father still alive** | **Family history** |
| **23643/13868(37511)** | **-0.48** | **-0.53** | **-0.43** | **4.82E-86** | **Mother still alive** | **Family history** |
| 37105/679(37784) | -0.33 | -0.49 | -0.18 | 3.18E-05 | PCT responsible for patient data: BRISTOL PCT | Summary Administration |
| 37556/228(37784) | -0.30 | -0.56 | -0.03 | 2.71E-02 | PCT responsible for patient data: HEYWOOD, MIDDLETON AND ROCHDALE PCT | Summary Administration |
| 37465/319(37784) | -0.28 | -0.50 | -0.05 | 1.63E-02 | PCT responsible for patient data: HAMPSHIRE PCT | Summary Administration |
| 36126/1658(37784) | -0.27 | -0.37 | -0.17 | 7.84E-08 | Illnesses of mother: Bowel cancer | Family history |
| 37157/251(37408) | -0.26 | -0.53 | 0.01 | 5.92E-02 | Delivery places: NHS hospital: consultant ward | Summary Maternity |
| 37503/281(37784) | -0.25 | -0.49 | -0.01 | 3.93E-02 | PCT responsible for patient data: RICHMOND AND TWICKENHAM PCT | Summary Administration |
| 37500/284(37784) | -0.25 | -0.49 | -0.01 | 3.88E-02 | Diagnoses - ICD10: L57.0 Actinic keratosis | Summary Diagnoses |
| 27033/10751(37784) | -0.24 | -0.29 | -0.20 | 2.88E-25 | Illnesses of mother: High blood pressure | Family history |
| 29487 | -0.24 | -0.26 | -0.22 | 1.33E-95 | Father's age at death | Family history |
| 37391/393(37784) | -0.23 | -0.44 | -0.03 | 2.33E-02 | Operative procedures - OPCS4: W20.1 Primary open reduction of fracture of long bone and extramedullary fixation using plate NEC | Summary Operations |
| 37290/490(37780) | -0.23 | -0.41 | -0.05 | 1.27E-02 | Operation code: removal of mole/skin lesion | Operations |
| 37161/250(37411) | -0.23 | -0.50 | 0.05 | 1.04E-01 | Intended delivery places: NHS hospital: consultant ward | Summary Maternity |
| 37316/434(37750) | -0.22 | -0.41 | -0.03 | 2.53E-02 | Manifestations of mania or irritability: I needed less sleep than usual | Mania |
| 37353/431(37784) | -0.22 | -0.41 | -0.02 | 2.90E-02 | PCT responsible for patient data: LIVERPOOL PCT | Summary Administration |
| 37030/754(37784) | -0.20 | -0.35 | -0.06 | 7.03E-03 | Diagnoses - ICD10: C44.3 Skin of other and unspecified parts of face | Summary Diagnoses |
| 37107/673(37780) | -0.20 | -0.35 | -0.04 | 1.17E-02 | Operation code: hip replacement/revision | Operations |
| 1548/633(2181) | -0.19 | -0.38 | 0.00 | 4.67E-02 | Errors before selecting correct item in numeric path (trail #1) | Trail making |
| 36981/803(37784) | -0.19 | -0.33 | -0.05 | 8.93E-03 | PCT responsible for patient data: OXFORDSHIRE PCT | Summary Administration |
| 37531/253(37784) | -0.19 | -0.44 | 0.06 | 1.40E-01 | Diagnoses - ICD10: C44.5 Skin of trunk | Summary Diagnoses |
| 37567/217(37784) | -0.19 | -0.46 | 0.08 | 1.74E-01 | Diagnoses - ICD10: K26.9 Unspecified as acute or chronic, without haemorrhage or perforation | Summary Diagnoses |
| 37093/691(37784) | -0.18 | -0.33 | -0.03 | 2.01E-02 | Diagnoses - ICD10: Z80.0 Family history of malignant neoplasm of digestive organs | Summary Diagnoses |
| 37574/206(37780) | -0.18 | -0.46 | 0.10 | 2.02E-01 | Non-cancer illness code, self-reported: heart arrhythmia | Medical conditions |
| 34995/2789(37784) | -0.18 | -0.25 | -0.10 | 1.04E-05 | Illnesses of father: Prostate cancer | Family history |
| 37507/277(37784) | -0.17 | -0.41 | 0.06 | 1.53E-01 | Operative procedures - OPCS4: W28.1 Application of internal fixation to bone NEC | Summary Operations |
| 24752/12756(37508) | -0.17 | -0.22 | -0.13 | 5.13E-15 | Qualifications: College or University degree | Sociodemographics |
| 37582/202(37784) | -0.17 | -0.45 | 0.11 | 2.30E-01 | Diagnoses - ICD10: R59.0 Localised enlarged lymph nodes | Summary Diagnoses |
| 37503/281(37784) | -0.17 | -0.41 | 0.07 | 1.58E-01 | Diagnoses - ICD10: Z09.8 Follow-up examination after other treatment for other conditions | Summary Diagnoses |
| 37476/304(37780) | -0.17 | -0.40 | 0.06 | 1.43E-01 | Operation code: bone surgery/joint surgery | Operations |
| 36679/1105(37784) | -0.17 | -0.29 | -0.05 | 6.34E-03 | Illnesses of mother: Lung cancer | Family history |
| 37573/211(37784) | -0.17 | -0.44 | 0.11 | 2.39E-01 | Operative procedures - OPCS4: Y80.4 Intravenous anaesthetic NEC | Summary Operations |
| 37021/460(37481) | -0.16 | -0.37 | 0.05 | 1.37E-01 | Status of baby at birth: Live | Summary Maternity |
| 26225/11448(37673) | -0.16 | -0.22 | -0.09 | 3.44E-06 | Medication for cholesterol, blood pressure or diabetes | Health and medical history |
| 37218/566(37784) | -0.15 | -0.32 | 0.01 | 7.42E-02 | Operative procedures - OPCS4: Z50.1 Skin of arm | Summary Operations |
| 37533/247(37780) | -0.15 | -0.41 | 0.10 | 2.39E-01 | Treatment/medication code: calcium salts | Medications |
| 37484/300(37784) | -0.15 | -0.38 | 0.08 | 1.97E-01 | Diagnoses - ICD10: M19.97 Arthrosis, unspecified (Ankle and foot) | Summary Diagnoses |
| 30669/7115(37784) | -0.15 | -0.20 | -0.10 | 1.43E-08 | Illnesses of mother: Heart disease | Family history |
| 37192/592(37784) | -0.15 | -0.31 | 0.01 | 7.29E-02 | Operative procedures - OPCS4: W37.1 Primary total prosthetic replacement of hip joint using cement | Summary Operations |
| 37091/339(37430) | -0.15 | -0.39 | 0.09 | 2.21E-01 | Resuscitation methods: Positive pressure nil, drugs nil | Summary Maternity |
| 746 | -0.15 | -0.29 | -0.01 | 4.23E-02 | U14 antigen for Human Herpesvirus-7 | Blood assays |
| 37407/373(37780) | -0.14 | -0.35 | 0.06 | 1.78E-01 | Non-cancer illness code, self-reported: sciatica | Medical conditions |
| 37563/221(37784) | -0.14 | -0.41 | 0.13 | 3.03E-01 | Operative procedures - OPCS4: H01.2 Emergency excision of abnormal appendix NEC | Summary Operations |
| 37520/260(37780) | -0.14 | -0.39 | 0.11 | 2.65E-01 | Operation code: mouth/salivary gland surgery | Operations |
| 37316/439(37755) | -0.14 | -0.33 | 0.05 | 1.55E-01 | Substances taken for depression: Unprescribed medication (more than once) | Depression |
| 37437/343(37780) | -0.14 | -0.36 | 0.08 | 2.12E-01 | Treatment/medication code: tamsulosin | Medications |
| 37416/368(37784) | -0.14 | -0.34 | 0.07 | 1.97E-01 | Operative procedures - OPCS4: E09.1 Excision of lesion of external nose | Summary Operations |
| 37264/520(37784) | -0.14 | -0.31 | 0.04 | 1.34E-01 | PCT responsible for patient data: SOUTH GLOUCESTERSHIRE PCT | Summary Administration |
| 37494/290(37784) | -0.14 | -0.37 | 0.10 | 2.57E-01 | PCT responsible for patient data: NOTTINGHAM CITY PCT | Summary Administration |
| 1638 | -0.13 | -0.23 | -0.04 | 4.33E-03 | Age at bilateral oophorectomy (both ovaries removed) | Sex-specific factors |
| 746 | -0.13 | -0.28 | 0.01 | 7.06E-02 | BK VP1 antigen for Human Polyomavirus BKV | Blood assays |
| 37529/255(37784) | -0.13 | -0.38 | 0.12 | 3.14E-01 | Operative procedures - OPCS4: Y80.1 Inhalation anaesthetic using muscle relaxant | Summary Operations |
| 37147/277(37424) | -0.13 | -0.39 | 0.13 | 3.39E-01 | Statuses of person conducting delivery: Midwife | Summary Maternity |
| 37538/246(37784) | -0.13 | -0.38 | 0.13 | 3.25E-01 | Diagnoses - ICD10: H25.8 Other senile cataract | Summary Diagnoses |
| 11241/26472(37713) | -0.13 | -0.17 | -0.08 | 8.76E-08 | Vascular/heart problems diagnosed by doctor | Health and medical history |
| 37436/348(37784) | -0.12 | -0.34 | 0.09 | 2.54E-01 | Operative procedures - OPCS4: S47.2 Drainage of lesion of skin NEC | Summary Operations |
| 37387/397(37784) | -0.12 | -0.34 | 0.10 | 2.78E-01 | Operative procedures - OPCS4: R24.9 All normal delivery | Summary Operations |
| 37324/460(37784) | -0.12 | -0.31 | 0.06 | 1.97E-01 | Operative procedures - OPCS4: O30.2 Splenic flexure | Summary Operations |
| 37460/324(37784) | -0.12 | -0.34 | 0.10 | 2.82E-01 | Diagnoses - ICD10: W19.9 Unspecified place | Summary Diagnoses |
| 37581/203(37784) | -0.12 | -0.40 | 0.16 | 4.07E-01 | Diagnoses - ICD10: S09.9 Unspecified injury of head | Summary Diagnoses |
| 37337/447(37784) | -0.12 | -0.30 | 0.07 | 2.27E-01 | Operative procedures - OPCS4: Z70.5 Lower end of radius NEC | Summary Operations |
| 23524/14192(37716) | -0.12 | -0.18 | -0.05 | 2.15E-04 | Medication for cholesterol, blood pressure, diabetes, or take exogenous hormones | Health and medical history |
| 7546 | -0.11 | -0.14 | -0.08 | 1.59E-13 | Father's age | Family history |
| 37497/287(37784) | -0.11 | -0.37 | 0.14 | 3.77E-01 | Delivery methods: Spontaneous vertex | Summary Maternity |
| 37579/205(37784) | -0.11 | -0.39 | 0.16 | 4.20E-01 | Histology of cancer tumour: Superficial spreading melanoma | Cancer register |
| 37520/260(37780) | -0.11 | -0.36 | 0.13 | 3.69E-01 | Operation code: adenoid surgery / adenoidectomy | Operations |
| 26457/11052(37509) | -0.11 | -0.16 | -0.07 | 1.16E-06 | Qualifications: A levels/AS levels or equivalent | Sociodemographics |
| 37575/209(37784) | -0.11 | -0.39 | 0.16 | 4.22E-01 | Diagnoses - ICD10: Z87.3 Personal history of diseases of the musculoskeletal system and connective tissue | Summary Diagnoses |
| 37320/464(37784) | -0.11 | -0.30 | 0.07 | 2.42E-01 | Operative procedures - OPCS4: W28.3 Removal of internal fixation from bone NEC | Summary Operations |
| 37149/635(37784) | -0.11 | -0.29 | 0.08 | 2.47E-01 | Hospital episode type: Delivery episode | Summary Administration |
| 36139/1641(37780) | -0.11 | -0.21 | -0.01 | 3.24E-02 | Operation code: reduction or fixation of bone fracture | Operations |
| 37536/248(37784) | -0.11 | -0.36 | 0.15 | 4.11E-01 | Type of cancer: ICD10: C44.5 Skin of trunk | Cancer register |
| 37158/622(37780) | -0.11 | -0.27 | 0.06 | 2.02E-01 | Operation code: laparoscopy | Operations |
| 36113/1667(37780) | -0.10 | -0.20 | 0.00 | 4.05E-02 | Operation code: varicose vein surgery | Operations |
| 37321/459(37780) | -0.10 | -0.29 | 0.08 | 2.80E-01 | Operation code: ovarian cyst removal/surgery | Operations |
| 37507/273(37780) | -0.10 | -0.34 | 0.14 | 4.03E-01 | Treatment/medication code: beconase 50micrograms nasal spray | Medications |
| 37297/487(37784) | -0.10 | -0.28 | 0.08 | 2.73E-01 | Operative procedures - OPCS4: Z47.3 Skin of cheek | Summary Operations |
| 35801/1983(37784) | -0.10 | -0.19 | -0.01 | 3.08E-02 | Illnesses of father: Bowel cancer | Family history |

N indicates the number of individuals considered for the PheWAS model being used for each respective UK Biobank trait. Traits with |Beta| >= 0.45 are highlighted.

**Supplementary Figure 1**

**
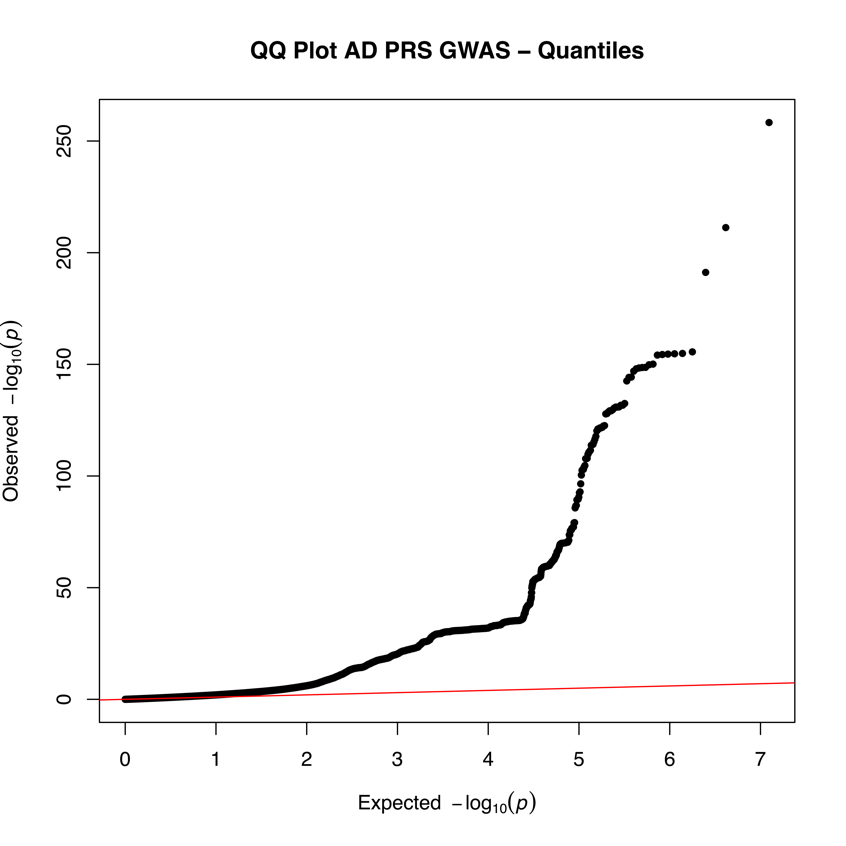
**
